# Genomic and Functional Dissections of Dickeya zeae Shed Light on the Role of Type III Secretion System and Cell Wall-Degrading Enzymes to Host Range and Virulence

**DOI:** 10.1128/spectrum.01590-21

**Published:** 2022-02-02

**Authors:** Ming Hu, Yang Xue, Chuhao Li, Mingfa Lv, Lianhui Zhang, Matthew R. Parsek, Guangtao Lu, Xiaofan Zhou, Jianuan Zhou

**Affiliations:** a Guangdong Laboratory for Lingnan Modern Agriculture, Guangdong Province Key Laboratory of Microbial Signals and Disease Control, Integrative Microbiology Research Center, South China Agricultural University, Guangzhou, China; b Department of Microbiology, University of Washington School of Medicine, Seattle, Washington, United States; c State Key Laboratory for Conservation and Utilization of Subtropical Agro-Bioresources, College of Life Science and Technology, Guangxi University, Nanning, China; South China Sea Institute of Oceanology, Chinese Academy of Sciences

**Keywords:** *Dickeya zeae*, host range, comparative genomics, virulence, T3SS, T3SEs, CWDEs

## Abstract

Dickeya zeae is a worldwide destructive pathogen that causes soft rot diseases on various hosts such as rice, maize, banana, and potato. The strain JZL7 we recently isolated from clivia represents the first monocot-specific D. zeae and also has reduced pathogenicity compared to that of other *D*. *zeae* strains (e.g., EC1 and MS2). To elucidate the molecular mechanisms underlying its more restricted host range and weakened pathogenicity, we sequenced the complete genome of JZL7 and performed comparative genomic and functional analyses of JZL7 and other *D*. *zeae* strains. We found that, while having the largest genome among *D*. *zeae* strains, JZL7 lost almost the entire type III secretion system (T3SS), which is a key component of the virulence suite of many bacterial pathogens. Importantly, the deletion of T3SS in MS2 substantially diminished the expression of most type III secreted effectors (T3SEs) and MS2’s pathogenicity on both dicots and monocots. Moreover, although JZL7 and MS2 share almost the same repertoire of cell wall-degrading enzymes (CWDEs), we found broad reduction in the production of CWDEs and expression levels of CWDE genes in JZL7. The lower expression of CWDEs, pectin lyases in particular, would probably make it difficult for JZL7 to break down the cell wall of dicots, which is rich in pectin. Together, our results suggest that the loss of T3SS and reduced CWDE activity together might have contributed to the host specificity and virulence of JZL7. Our findings also shed light on the pathogenic mechanism of *Dickeya* and other soft rot *Pectobacteriaceae* species in general.

**IMPORTANCE** Dickeya zeae is an important, aggressive bacterial phytopathogen that can cause severe diseases in many crops and ornamental plants, thus leading to substantial economic losses. Strains from different sources showed significant diversity in their natural hosts, suggesting complicated evolution history and pathogenic mechanisms. However, molecular mechanisms that cause the differences in the host range of *D*. *zeae* strains remain poorly understood. This study carried out genomic and functional dissections of JZL7, a *D*. *zeae* strain with restricted host range, and revealed type III secretion system (T3SS) and cell wall-degrading enzymes (CWDEs) as two major factors contributing to the host range and virulence of *D*. *zeae*, which will provide a valuable reference for the exploration of pathogenic mechanisms in other bacteria and present new insights for the control of bacterial soft rot diseases on crops.

## INTRODUCTION

*Dickeya* bacteria usually cause soft rot disease on many economically important crops and ornamental plants all over the world. As a highly diverse group, the genus contains 12 species according to current classification criteria, namely, D. chrysanthemi, D. dadantii, D. dianthicola, D. paradisiaca, D. zeae, D. solani, D. aquatica, D. fangzhongdai, D. poaceaephila, D. lacustris, D. undicola, and D. oryzae (1–8). Among them, *D*. *zeae* was isolated mostly from plants in tropical and subtropical regions, especially in Southeast Asian countries (9), suggesting its preference to high temperature and humidity. On the other hand, *D*. *solani* was usually isolated from potato in temperate and frigid zones, especially across Europe (10, 11), with a wider temperature range for growth and disease development (12).

Although isolated mainly from monocotyledonous hosts in nature, *D*. *zeae* is so far known to infect 24 types of dicots and 23 types of monocots, indicating a broad host range (9). In recent years, *D*. *zeae* has caused considerable losses in crop yields in China, especially on rice and banana (13–16). At the same time, *D*. *zeae* strains from diverse sources also display considerable variations in their pathogenesis. For instance, the strains EC1 (isolated from rice) and MS2/MS3 (isolated from banana) showed different virulence on various monocot and dicots plants and exhibited 2-fold difference in their ability to inhibit rice seed germination (9, 17). Furthermore, consistent with these phenotypic variations, there are also key genomic differences between EC1 and MS2/MS3, including the *zms* gene cluster encoding the phytotoxic zeamines, one of the most important virulence factors unique to and shared by rice strains isolated from geographically distant regions (9, 18, 19), the biosynthetic gene cluster (C1O30_RS04995 to C1O30_RS05185) encoding a novel phytotoxin in MS2, and the *pipR* and *pipA* that are present only in MS1 and MS2 (17).

Importantly, we have recently isolated from the ornamental plant Clivia miniata three *D*. *zeae* strains which are, for the first time, found to specifically infect monocots (9). These strains were pathogenic on nearly all tested monocots (e.g., rice, banana, clivia), although their virulence was usually lower than that of other *D*. *zeae* strains, and could not infect any of the nine dicots tested (e.g., potato, tomato, radish, cabbage) (9). A distinctive characteristic of these clivia-isolated strains is the remarkably lower activity of cell wall-degrading enzymes (CWDEs) compared with that of other *D*. *zeae* (9). However, the molecular basis of the dramatic alteration in host range remains largely unknown.

To investigate determinants of the host range of *D*. *zeae*, we sequenced the genome of JZL7, one of the monocot-specific strains isolated from C. miniata, and performed comparative genomic as well as functional analyses of JZL7 and other *D*. *zeae* strains that infect both monocots and dicots. The findings of this study will facilitate our understanding of the nature and the evolution of the pathogenicity mechanisms of *D*. *zeae*.

## RESULTS AND DISCUSSION

### General genomic features of *D*. *zeae* strain JZL7.

**FIG 1:**
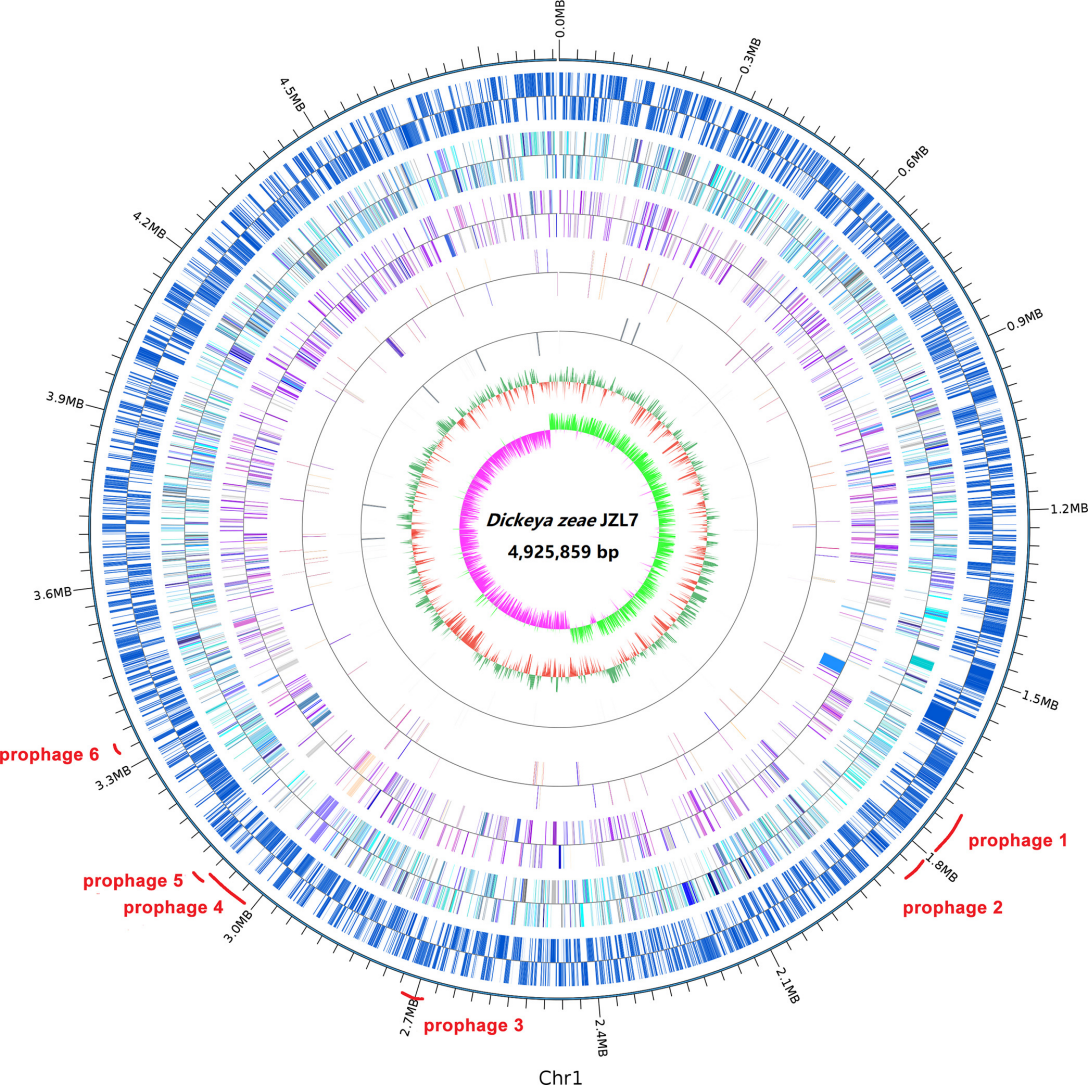
Circular visualization of genome characteristics of *D*. *zeae* strain JZL7. The circles from outermost to innermost indicate the distributions of protein-coding genes, COG annotations, KEGG annotations, GO annotations, rRNAs, GC ratio, and GC skew, respectively.

The genome of *D*. *zeae* strain JZL7 was sequenced on both the PacBio RSII and the Illumina HiSeq X Ten platforms, producing 196,305 long sequencing reads (*N*_50_: 8,071 bp; mean read length: 5.356 Kb; total size: 1.051 Gb) and 5.44 million short sequencing reads (PE150; total size: 1.789 Gb), respectively. A hybrid *de novo* assembly using both the short and long sequencing reads resulted in a high-quality and gapless assembly consisting of a single circular chromosome of 4,925,859 bp in size; no plasmids were detected (Fig. 1). BUSCO evaluation of the JZL7 genome detected 438 of the 440 genes (99.5%) in the enterobacterales_odb10 data set, suggesting that the assembly is highly complete. The JZL7 genome contains 4,268 protein-coding genes, 75 tRNA genes, and 22 rRNA genes which belong to seven complete rRNA loci, including the unusual 16S-23S-5S-5S operon previously observed in other *Dickeya* genomes (Table 1) (18). JZL7 has both the largest genome and the largest repertoire of protein-coding genes among all sequenced *D*. *zeae* strains; it contains 167 more genes than Ech586, which has the second largest genome, and 443 more genes than EC1, which has the smallest genome (Table 1). On the other hand, the G+C content of JZL7 genome (53.68%) is highly similar to that of the other *D*. *zeae* genomes (53.34% to 53.65%) (Table 1).

**TABLE 1:** Genomic features of the six *D*. *zeae* strains with complete genome

Features	JZL7	MS2	EC1	EC2	CE1	Ech586
BUSCO (complete)	99.5%	99.5%	99.8%	99.8%	99.5%	99.5%
No. of replicons	1	1	1	1	1	1
Size (bp)	4,925,859	4,740,052	4,532,364	4,575,125	4,714,731	4,818,394
G+C content (%)	53.68	53.45	53.43	53.34	53.65	53.64
Genes	4,372	4,171	3,947	3,985	4,143	4,205
Protein-coding genes	4,268	4,068	3,825	3,879	4,037	4,101
rRNAs	22	22	22	22	22	22
tRNA	75	75	88	75	75	76
ncRNA	3	2	8	5	5	2
Pseudo genes	112	50	65	74	71	64
Transposases	47	48	38	59	25	22
Prophage						
Region	9	3	4	4	4	3
Gene	250	68	160	63	108	87
Genomic island						
Region	35	24	21	25	19	30
Gene	657	355	364	357	337	346

### Genomic differences between JZL7 and other *D*. *zeae* strains.

**FIG 2:**
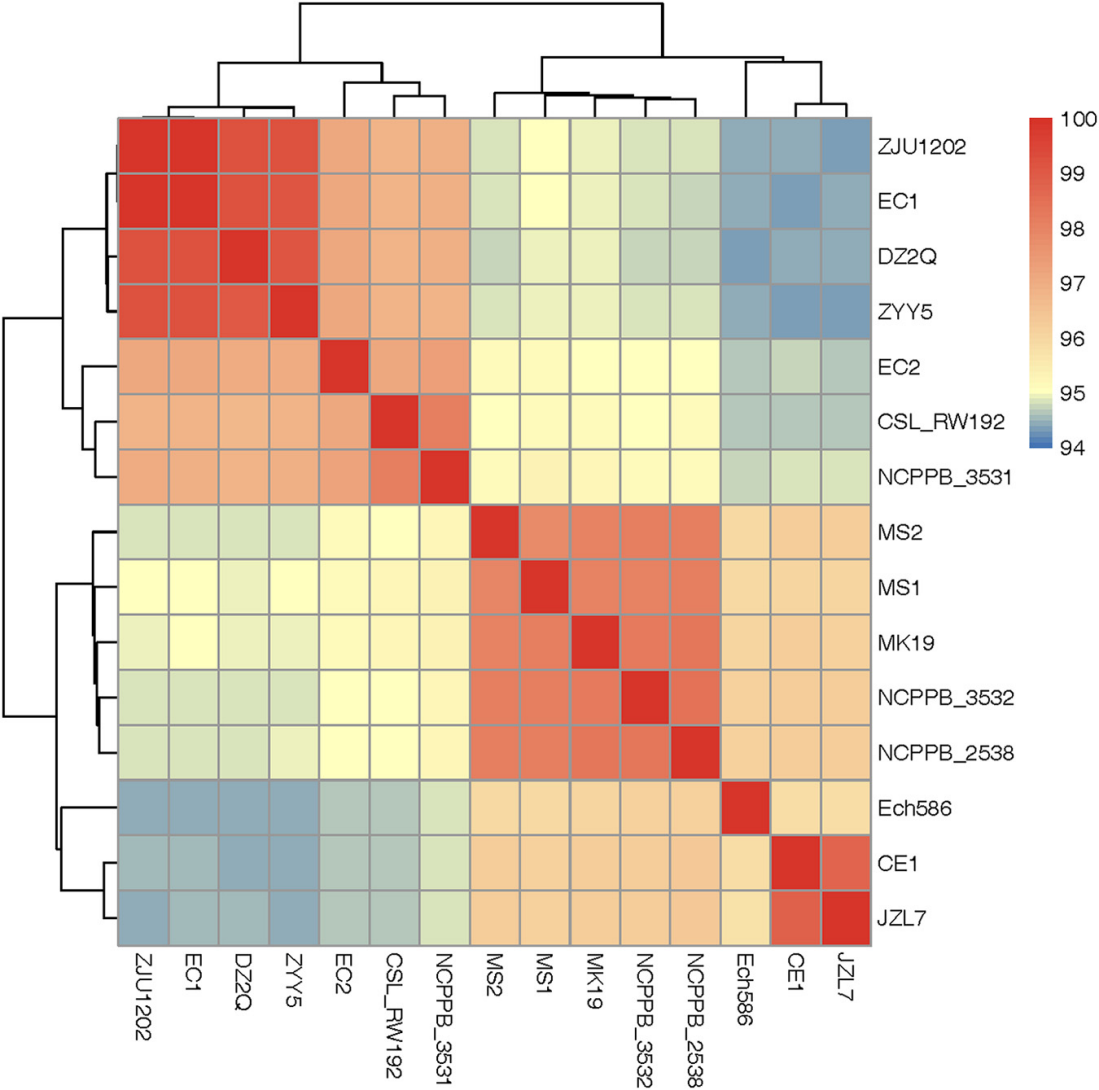
ANI analyses of 15 *D*. *zeae* and *D*. *oryzae* strains. Based on their pairwise ANI values, all strains were clustered into two well-separated groups corresponding to *D*. *zeae* (type strain: NCPPB 2538) and *D*. *oryzae* (type strain: ZYY5), respectively. JZL7 belongs to the *D*. *zeae* group and the ANI values between JZL7 and all other *D*. *zeae* strains are greater than 95%.

To determine the evolution relationship between JZL7 and other *D*. *zeae* strains, all sequenced *D*. *zeae* and the closely related *D*. *oryzae* ZYY5 (8) genomes were used for phylogenetic analysis based on the 3,110 single-copy core genes (present in all strains). Results showed that the 15 *D*. *zeae* strains can be divided into two clusters (Fig. 2); the average nucleotide identity (ANI) values are above 95% for all pairwise comparisons within each cluster but below 95% for nearly all comparisons between clusters (Fig. 2). The first cluster consists of seven strains, including DZ2Q, EC1, EC2, ZJU1202, and ZYY5, the five rice-pathogenic strains, as well as CSL_RW192 and NCPPB_3531. Our results thus corroborate the recent proposal by Wang et al. (8) to classify this clade as a novel species, *Dickeya oryzae*.

The second clade contains JZL7, CE1, and Ech586, as well as five other strains that are highly similar to each other (ANI values of >97.85%), namely, MS1, MS2, MK19, NCPPB_2538, and NCPPB_3532 (Fig. 2). We have recently shown that EC1 (first clade) and MS2 (second clade) are pathogenic toward both monocot and dicot hosts, whereas JZL7 (second clade) can infect only monocots (9). Taken together, our results suggest that the broad host range may have been the ancestral state to both *D*. *zeae* and *D*. *oryzae*, while JZL7 became specialized on monocots during recent evolution.

**FIG 3:**
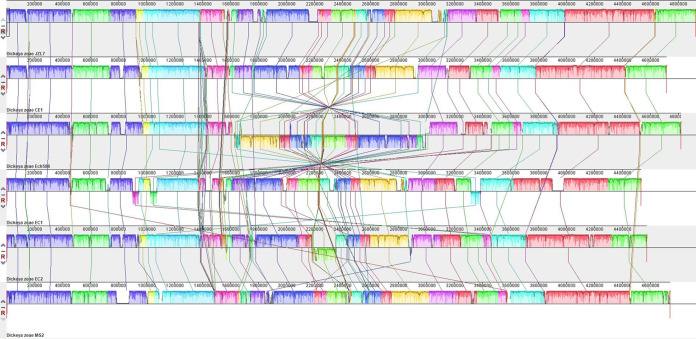
Whole-genome comparison between *Dickeya* strains JZL7, CE1, Ech586, EC1, EC2, and MS2. The genome structures are largely conserved among the strains, with only a few large-scale rearrangements and much more frequent small-scale insertions and deletions.

To characterize the genomic differences between JZL7 and other *D*. *zeae* and *D*. *oryzae* strains that might underlie their divergent host specificities (Table S1), we first constructed whole-genome alignment between JZL7, CE1, Ech586, EC1, EC2, and MS2, all of which have completely sequenced genomes. Results showed that the six strains are largely conserved in their overall genome structures, with the only exceptions being a large inversion of ∼1.4 Mb in the genome of Ech586 and a few smaller translocations in the genomes of EC1 and EC2 (Fig. 3). At the same time, insertions/deletions up to several tens of kilobases are frequently observed and distributed throughout the six genomes (Fig. 3).

**FIG 4:**
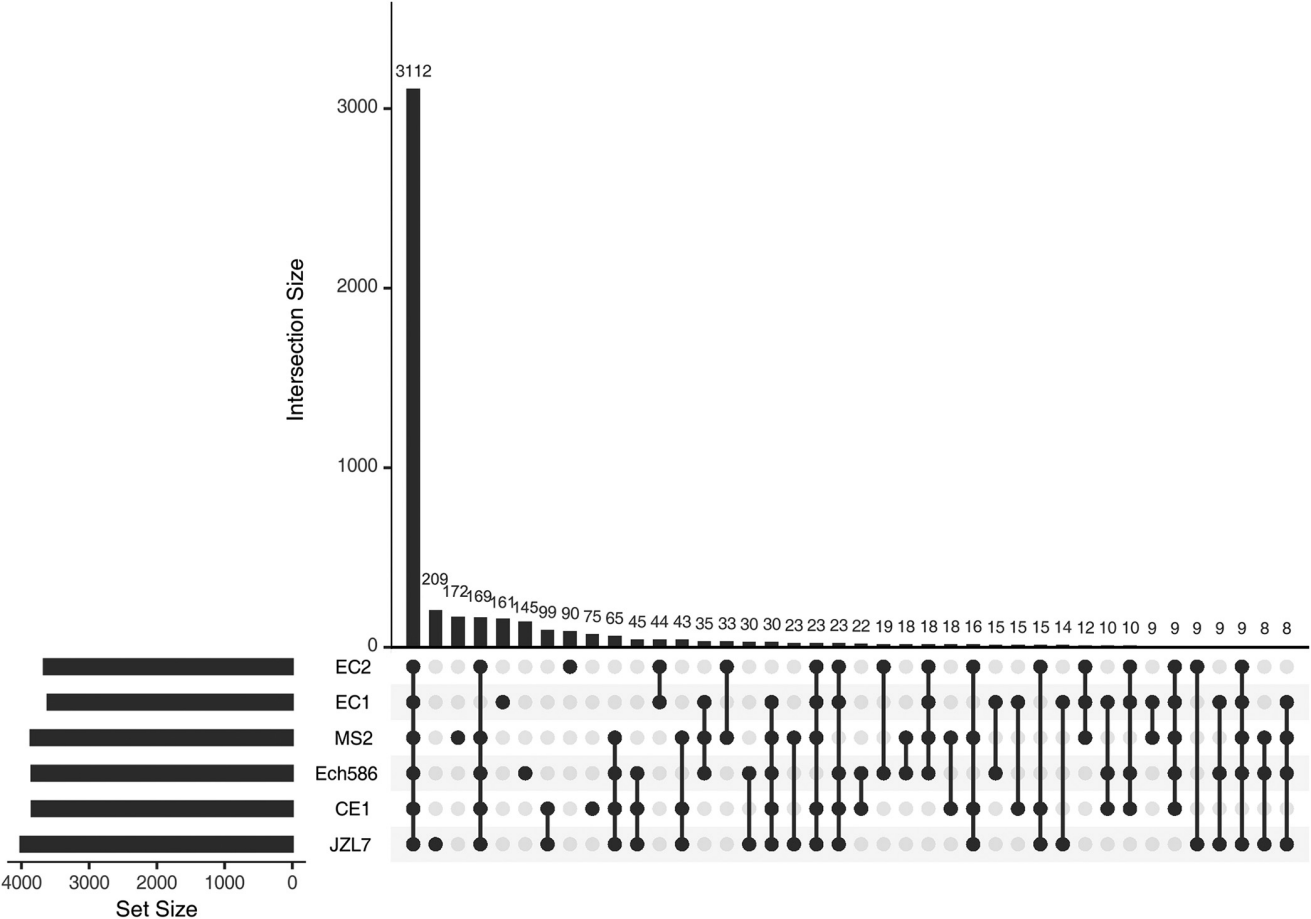
A diagram UpSet plot showing the results of pangenome analysis of JZL7, CE1, Ech586, EC1, EC2, and MS2.

We then performed pangenome analyses on the six strains to further examine differences in their gene contents. In total, 4,966 distinct gene families were identified, the majority of which (3,112 families; 62.67%) are shared by all six strains (Fig. 4), indicating a relatively stable core genome. On the other hand, 209 ortholog group family genes are JZL7 specific (Table S4), whereas 9 gene families are absent in JZL7 but shared by all the other five strains, which may contain genes that are critical for the pathogenicity to dicots.

Among the unique genes in the JZL7 genome, an additional set of type IV secretion system (T4SS) that consists of 14 *trb* genes was detected (Table S5). This extra T4SS in JZL7 shares high levels of nucleotide sequence similarity to the T4SS-encoding gene sets in Ralstonia solanacearum strains FQY_4 and GMI1000, Pseudomonas aeruginosa F30658, and Pectobacterium carotovorum subsp. *brasiliense* strain SX309. T4SS usually functions in the translocation of nucleic acids or proteins from donor to recipient by conjugation and DNA release/uptake (20). The two sets of T4SSs encoded in the JZL7 genome (Table S5) may have contributed to the abundant strain-specific genes of JZL7 (Fig. 1 and 3, Tables S6 and S7). However, given that strain JZL7 has more restricted host range than the strains EC1 and MS2 (9), we conjectured that this extra T4SS is not important for the pathogenesis of *D*. *zeae*.

### The loss of T3SS in JZL7 partially explains its restricted host range.

**FIG 5:**
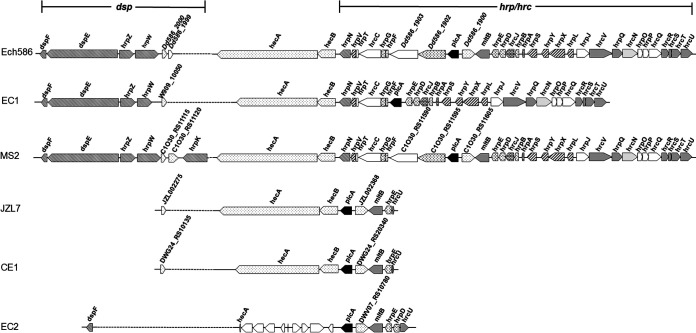
Gene arrangements of T3SS in genomes of strains Ech586, EC1, MS2, JZL7, CE1, and EC2.

The more restricted host range of JZL7 (Table S1) might be due to the loss of some vital determinants of virulence or host specificity during its evolution. Accordingly, we found in our pangenome analysis 85 gene families that are shared by both EC1 and MS2 but absent in JZL7 (Fig. 4, Table S8), including a set of 30 genes that are colocalized in the genome; they comprise the majority of the *dsp* gene cluster, which encodes multiple T3SS secreted proteins (e.g., DspE, HrpZ, and HrpW) as well as their chaperone (DspF), and the *hrp/hrc* gene cluster, which encodes a T3SS (Fig. 5, Table S8). The *hecA*-*hecB* genes between the *dsp* and *hrp/hrc* clusters, however, were conserved in JZL7; they encode the type V secretion system (T5SS), which contributes to the adherence to hosts (21). Exactly the same organization in this genomic region was found in CE1, the closest relative of JZL7 (Fig. 5). Interestingly, the *D*. *oryzae* strain EC2 also lost its *dsp* and *hrp/hrc* clusters, but it has a substantially different set of remaining genes.

T3SS forms a syringe-like structure to directly translocate type III secreted effectors (T3SEs) from bacterial cells into host cells and is considered a key determinant for virulence in many bacteria. It has been reported in a number of plant bacterial pathogens (e.g., Erwinia pyrifoliae, Ps. syringae, and Xanthomonas campestris pv. *campestris*) that the loss of the T3SS would severely reduce the elicitation of host defenses and thus lead to attenuated disease symptoms (22–24). Moreover, T3SS was demonstrated to be required for the full virulence of *D*. *dadantii* 3937 (25, 26).

**FIG 6:**
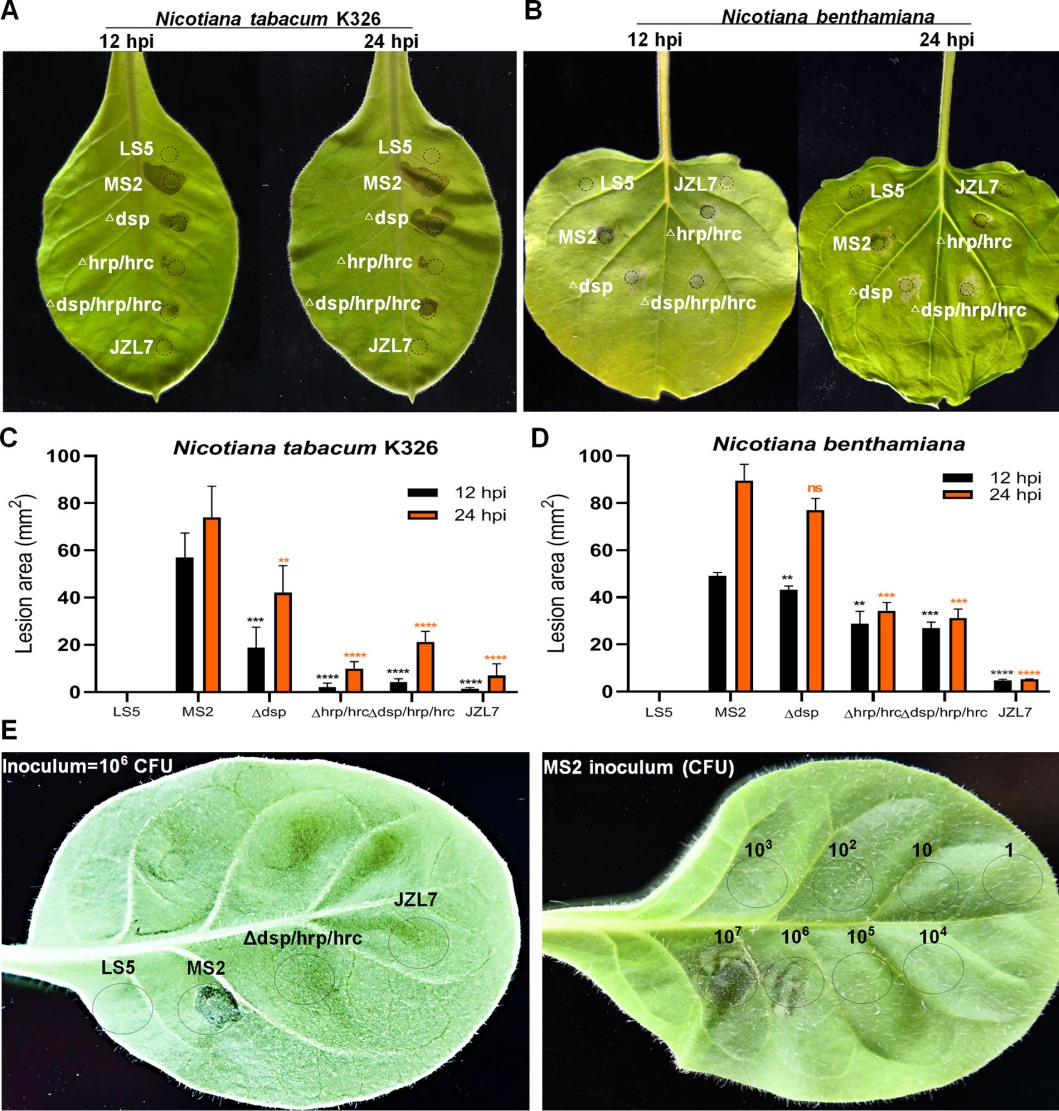
The HR of the T3SS mutants of MS2 and strain JZL7 on Nicotiana tabacum K326 (A, C, and E) and N. benthamiana (B and D) after 12 and 24 hpi. Bacterial cultures (OD_600_ of 1.0 in LS5 medium) were dipped by a puncher (5 mm), pressed on the back of tobacco leaves, and incubated at 28°C. The area of HR lesions was measured using Image J 1.52a after 12 and 24 h. **, *P < *0.001; ***, *P < *0.0001; ****, *P < *0.0001; ns, not significant (Student’s *t* test, *n* = 3 independent experiments). (E) Quantitative determination of the T3SS functions in hypersensitive response indicated that the threshold of 10^6^ CFU of MS2 elicits HR at 12 h.

To evaluate whether strain JZL7 could elicit hypersensitive response (HR) as strain MS2 harboring T3SS, we performed HR assay on Nicotiana tabacum K326 (27) and Nicotiana benthamiana (28). Results indicated that strain JZL7 could not cause HR on either of the tobacco leaves, the same as the negative-control LS5 medium, whereas strain MS2 elicited a typical HR quickly in the early stage of inoculation (12 hpi) (Fig. 6). To further verify the importance of T3SS, we respectively deleted the *dsp* cluster, the *hrp/hrc* cluster, and both in MS2. HR induction of Δ*dsp* was substantially reduced in the early stage of inoculation compared with that of wild-type MS2, but little difference was found between them after 24 hpi (Fig. 6), suggesting that the T3SEs DspE, HrpZ, HrpW, and HrpK function in the initial infection of plant hosts. The T3SS structure deletion mutant Δ*hrp*/*hrc* almost could not induce HR both in the early and later stages of infection, while the whole T3SS deletion mutant Δ*dsp*/*hrp*/*hrc* significantly attenuated the HR reaction at 12 hpi but recovered part of it at 24 hpi (Fig. 6). HR is a form of programmed cell death (PCD) at the site of pathogen infection commonly controlled by interactions between pathogen avirulence gene products and plant resistance genes. Although the T3SS has been deleted from strain MS2, some avirulence genes, like the known *avrL* (*C1O30_RS14255*), are still present in the genome; thus, reduced HR could be observed by mutant infiltration. Furthermore, the components of the T3SS and the T3SEs are not the only pathogen-associated molecular patterns (PAMPs) that plant defenses are known to recognize. Flagellin, peptidoglycan, and lipopolysaccharides also contribute to triggering host innate immune response through recognizing by plant pattern recognition receptors (PRRs). To quantify the function of the T3SS, we infiltrated tobacco leaves with controlled inoculum size. Results showed that the threshold of 10^6^ MS2 CFU elicited visible HR at 12 h (Fig. 6E), which may help distinguish between true HR and other types of PCD.

**FIG 7:**
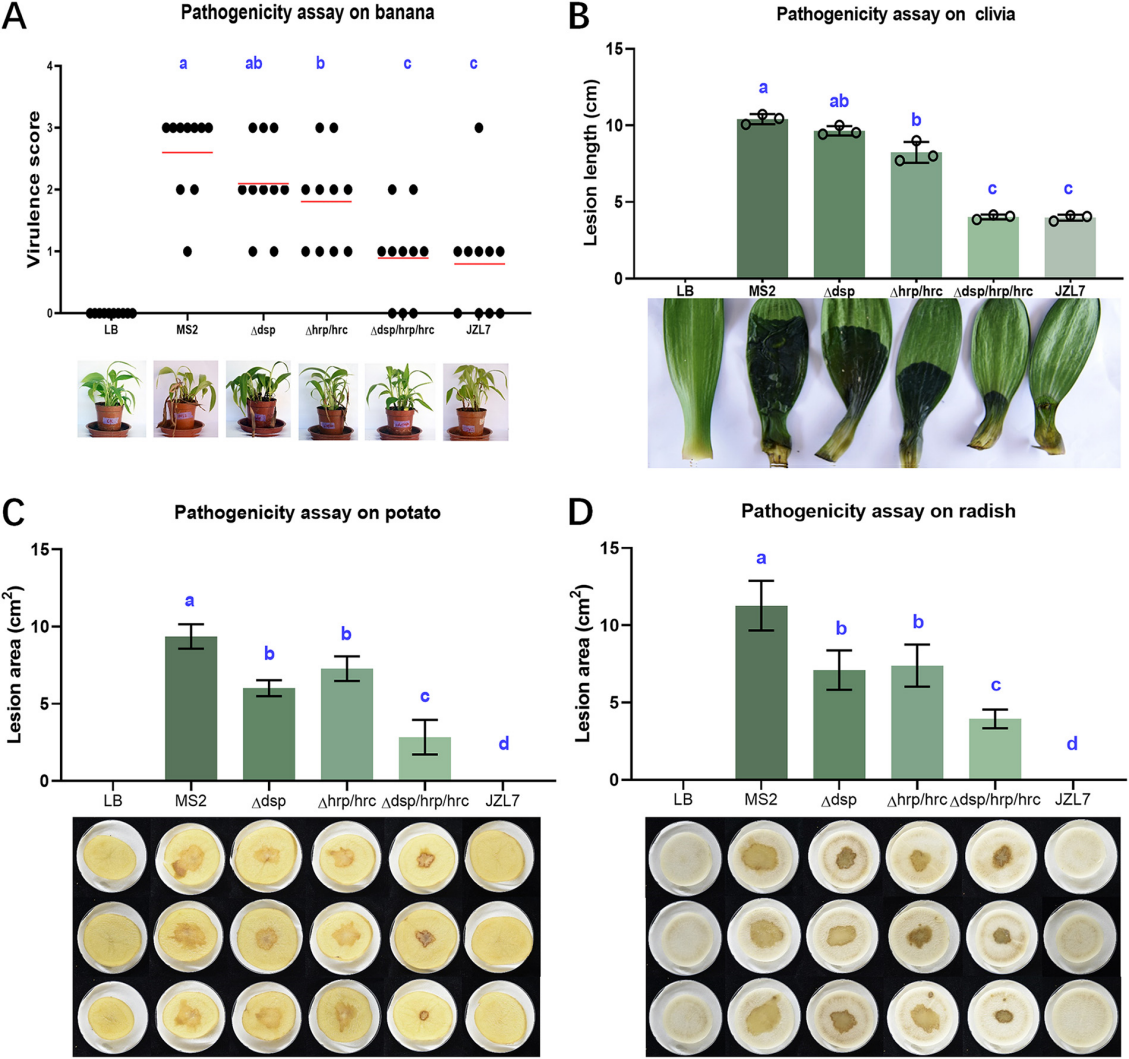
The virulence of the T3SS mutants of MS2 and strain JZL7 on monocotyledonous and dicotyledonous plants. (A) Strain inoculation on banana seedlings. Every 100 μL of bacterial culture (OD_600_ of 1.2 in LB medium) was injected into the basal stem and incubated at 30°C with 12-h alternating light-dark cycles for 7 days. The disease was assessed using the virulence scoring method described previously (17, 80). Red lines indicate the mean virulence score of the strains. (B) Strain inoculation on clivia leaves. Every 100 μL of bacterial culture was injected into the base of clivia leaf. (C and D) Strain inoculation on potato and radish slices. Bacterial cells of 2 μL were applied to the center of the tuber slices. Same volume of LB medium was inoculated as a negative control. All plants were kept in a growth chamber with controlled conditions of 28 ± 2°C, 75% ± 15% relative humidity and 24-h white light (7,350 lx) illumination until symptoms appeared. Visible macerate areas on clivia and potato and radish slices were measured using Image J 1.52a. Statistical analysis was performed on each group of data, and significantly different values (ANOVA *P* < 0.05) are indicated by different letters.

To further investigate the role of T3SS in the pathogenicity and host specificity of *D*. *zeae* pathogens, we tested the pathogenicity of Δ*dsp*, Δ*hrp*/*hrc*, and Δ*dsp*/*hrp*/*hrc* mutants in strain MS2 on monocotyledonous banana stems and clivia leaves, as well as dicotyledonous potato and radish slices. We found that the Δ*hrp*/*hrc* showed 30% reduced virulence on banana and clivia seedlings (Fig. 7A and B) and 22% and 24% reduced virulence on potato and radish slices, respectively, compared with wild-type MS2 (Fig. 7C and D). The Δ*dsp* mutant similarly showed lower (35% on potato and 36% on radish) virulence on dicots, whereas its virulence on monocots was 20% reduced on banana seedlings and 7% reduced on clivia seedlings, not significantly different from that of MS2. Notably, the deletion of the whole T3SS (Δ*dsp*/*hrp*/*hrc*) dramatically attenuated the virulence of MS2 by more than 60% on monocots (Fig. 7A and B), to a level that is comparable to that of JZL7, and by more than 65% on dicots (Fig. 7C and D). Altogether, our results indicate that T3SS has a major role in the virulence of *D*. *zeae*. Previous studies have revealed that tissue maceration requires type II secretion (T2SS) of CWDEs and does not involve T3SS (18). Our study revealed the contribution of T3SS to the development of soft rot symptoms, which probably results from the action by the T3SEs regulated by HrpL, as shown in Fig. S1. Unlike JZL7, however, the Δ*dsp*/*hrp*/*hrc* mutant of MS2 still exhibited considerable virulence on dicots, suggesting that there are likely other factors contributing to the host specificity of *D*. *zeae* pathogens in addition to T3SS.

### HrpL regulates most predicted T3SEs in MS2.

In addition to the syringe structure of T3SS, genes in the *dsp* and *hrp/hrc* clusters also encode multiple transcription regulators and effectors. We generated single-gene knockout mutants for selected regulator (HrpL HrpS, HrpX, and HrpY) or effector (DspE, HrpK, HrpN, HrpW, and HrpZ) coding genes in MS2 and tested their pathogenicity on potato slices. Results showed that three regulator gene mutants (Δ*hrpL*, Δ*hrpX*, and Δ*hrpY*) and three effector gene mutants (Δ*hrpK*, Δ*hrpN*, and Δ*hrpZ*) exhibited significantly reduced virulence compared with that of MS2 wild type (Fig. S1). In particular, the Δ*hrpL* mutant showed the most dramatic reduction in virulence, which is comparable to the knockout of the entire T3SS (Δ*dsp*/*hrp*/*hrc*) (Fig. S1).

**FIG 8:**
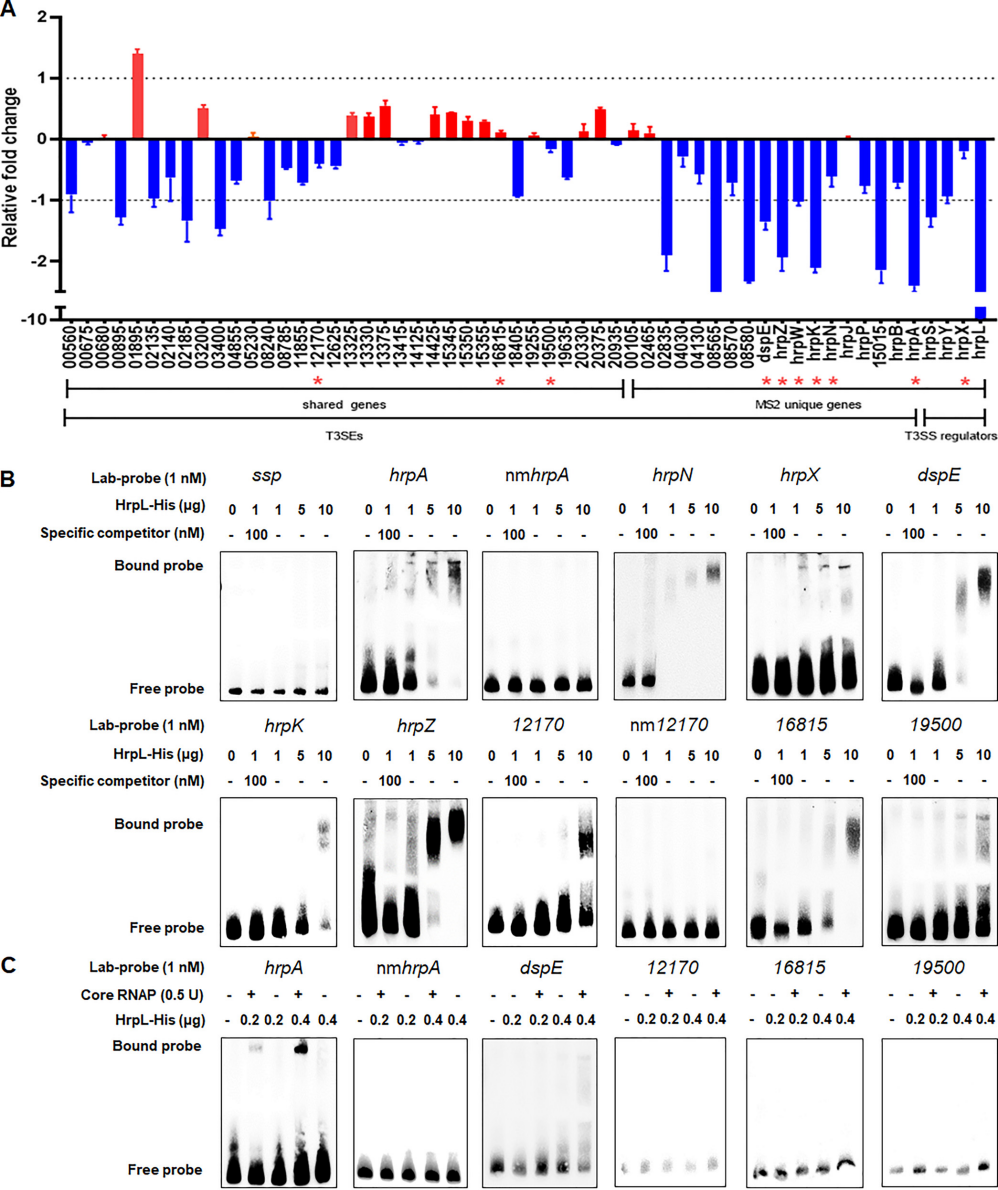
The T3SEs regulated by the HrpL regulator. (A) qRT-PCR of the predicted T3SEs in the genome of MS2. The expression of the predicted T3SE encoding genes (listed in Table 2), including 34 genes shared in both MS2 and JZL7 genomes and 15 genes present uniquely in the MS2 genome, as well as the regulatory genes *hrpX*, *hrpY*, *hrpS*, and *hrpL*, were measured by qRT-PCR. Expression of the housekeeping gene *atpD* was used as a reference. The *y* axis indicates the values log_2_(fold change of Δ*hrpL* mutant relative to wild-type MS2). Red bar indicates expression levels higher in the mutant, while blue indicates those lower in the mutant. Red stars indicate the target genes whose promoters were verified to be interacted by HrpL protein in the panel below. (B) EMSA of the T3SEs with predicted *hrp* box. The promoter DNA fragments of the genes *C1O30_RS12170*, *C1O30_RS16815*, *C1O30_RS19500*, *dspE*, *hrpZ*, *hrpK*, and *hrpN* and the known HrpL-regulated gene *hrpA*, which contain the predicted *hrp* box in Table 2, were amplified and labeled by biotin and then performed for EMSA with different concentrations of the expressed and purified HrpL protein. Fragments without *hrp* box (non GGAACC-Nx-CCACNNA motif) in the *hrpA* and *C1O30_RS12170* promoters, designated nm*hrpA* and nm*12170*, respectively, were amplified and used for EMSA reaction to confirm the importance of the presence of *hrp* box. For specific competition, a 100 nM unlabeled DNA fragment was incubated with 1 μg HrpL protein for 15 min before addition of a 25 nM labeled DNA fragments. (C) E. coli core RNAP (0.5 U) was incubated on ice for 20 min with 0.2 or 0.4 μg of purified HrpL protein. The remaining steps were as described above.

HrpL is a sigma factor activating T3SS at different levels, including gene transcription, mRNA stability, and enzymatic activity in many plant-pathogenic bacteria, such as *Erwinia*, *Pseudomonas*, *Ralstonia*, and *Xanthomonas* (25, 29–34). In *Dickeya* bacteria, however, only one effector, DspE, has been characterized alongside two harpins, HrpN and HrpW (35, 36). To investigate the regulatory role of HrpL, we first used a combination of four state-of-the-art bioinformatic tools (i.e., Bastion3, BEAN2, DeepT3, and pEffect) to identify candidate T3SEs in the genomes of MS2 and JZL7. As a result, we predicted 34 T3SEs that are present in both strains, as well as 16 and 10 T3SEs that are unique to MS2 and JZL7, respectively (Table 2). Given that *hrpL* is absent in JZL7, we measured the expression of all T3SE genes in MS2 wild type and Δ*hrpL* mutant using quantitative reverse transcriptase PCR (qRT-PCR). Results showed that the expression of 5 shared T3SE and 8 MS2-unique T3SE genes was significantly downregulated in the Δ*hrpL* mutant, including *dspE*, *hrpA*, *hrpK*, *hrpS*, *hrpW*, and *hrpZ*, which are all members of the *dsp* and *hrp/hrc* clusters (Fig. 8A). Chi-square tests on the fold change values of shared versus unique T3SEs showed that HrpL regulates the expression of the MS2-unique T3SE genes more strongly than the shared ones.

**TABLE 2:** T3SE candidates predicted in the genomes of Dickeya zeae MS2 and JZL7

Accession no. of:		NCBI accession no.	Product	Predicted *hrp* box (GGAACC-Nx-CCACNNA)
MS2	JZL7			
C1O30_RS00560	JZL000100	WP_013319992.1	Hypothetical protein VfmS	
C1O30_RS00675	JZL000138	WP_102800815.1	Hypothetical protein	
C1O30_RS00680	JZL000139	WP_102800816.1	Hypothetical protein	
C1O30_RS00895	JZL000183	WP_102800840.1	Murein hydrolase activator EnvC	
C1O30_RS01895	JZL000383	WP_102800904.1	Type I-E CRISPR-associated protein Cse2/CasB	
C1O30_RS02135	JZL000448	WP_102800932.1	Hypothetical protein	
C1O30_RS02140	JZL000451	WP_102800933.1	Hypothetical protein	
C1O30_RS02185	JZL000460	WP_102800940.1	Hypothetical protein	
C1O30_RS03200	JZL000666	WP_102801017.1	Glycine dehydrogenase GcvP	
C1O30_RS03400	JZL000710	WP_019846222.1	Hypothetical protein	
C1O30_RS04855	JZL000906	WP_019844323.1	LOG family protein	
C1O30_RS05230	JZL000962	WP_023639126.1	Hypothetical protein	
C1O30_RS08240	JZL001538	WP_102801528.1	Alginate lyase family protein	
C1O30_RS08785	JZL001698	WP_023639967.1	Hypothetical protein	
C1O30_RS11855	JZL002390	WP_012884524.1	DUF1852 domain-containing protein	
C1O30_RS12170	JZL002462	WP_102801891.1	Hypothetical protein	−241 **GGAAC**T-N69-**CCACCGA** −133
C1O30_RS12625	JZL002555	WP_102801925.1	Anhydro-N-acetylmuramic acid kinase AnmK	
C1O30_RS13325	JZL002697	WP_019843821.1	Flagellin FliC	
C1O30_RS13330	JZL002698	WP_102801989.1	Flagellar filament capping protein FliD	
C1O30_RS13375	JZL002707	WP_102801991.1	Flagellar hook-length control protein FliK	
C1O30_RS13415	JZL002715	WP_102801993.1	Flagellar hook-filament junction protein FlgL	
C1O30_RS14125	JZL002985	WP_102802039.1	Flagella biosynthesis regulator Flk	
C1O30_RS14425	JZL003042	WP_023640291.1	General secretion pathway protein GspB	
C1O30_RS15345	JZL003254	WP_028085677.1	Pectate lyase PelA	
C1O30_RS15350	JZL003255	WP_102802153.1	Pectate lyase PelE	
C1O30_RS15355	JZL003256	WP_102802154.1	Pectate lyase PelD	
C1O30_RS16815	JZL003523	WP_102802268.1	Hypothetical protein	−590 **GGAAC**A-N56-G**CACCAA** −522
C1O30_RS18405	JZL003848	WP_102802410.1	Hypothetical protein	
C1O30_RS19255	JZL004042	WP_102802473.1	SPOR domain-containing protein	
C1O30_RS19500	JZL004096	WP_023640946.1	Hypothetical protein	+22 **GGAAC**G-N37-T**CACCAA** +71
C1O30_RS19635	JZL004123	WP_102802502.1	Uroporphyrinogen-III C-methyltransferase HemX	
C1O30_RS20330	JZL004262	WP_102802565.1	Four-carbon acid sugar kinase family protein	
C1O30_RS20375	JZL004272	WP_102802574.1	Cell envelope biogenesis protein TolA	
C1O30_RS20935	JZL004396	WP_038906084.1	Der GTPase-activating protein YihI	
C1O30_RS00105		WP_102800757.1	Hypothetical protein	
C1O30_RS02465		WP_102800962.1	Hypothetical protein	
C1O30_RS02835		WP_038915541.1	Pectate lyase Pnl	
C1O30_RS04030		WP_102801114.1	Hypothetical protein	
C1O30_RS04130		WP_102801119.1	TonB family protein	
C1O30_RS08565		WP_102801570.1	Hypothetical protein	
C1O30_RS08570		WP_102801571.1	Hypothetical protein	
C1O30_RS08580		WP_102801572.1	Hypothetical protein	
C1O30_RS11100		WP_102801796.1	Type III secretion system effector DspE	−82 **GGAACC**-N15-**CCACTCA** −55
C1O30_RS11105		WP_102801797.1	Type III effector protein HrpZ	−110 **GGAACC**-N16-T**CACTCA** −82
C1O30_RS11110		WP_102801798.1	Pectate lyase HrpW	−98 **GGAAC**T-N15-GT**ACTCA** −71
C1O30_RS11125		WP_102801801.1	Type III effector HrpK	−70 **GGAAC**T-N15-**CCACTCA** −43
C1O30_RS11560		WP_023639571.1	Harpin HrpN	−125 **GGAACC**-N15-T**CACTCA** −98
C1O30_RS11660		WP_102801841.1	Type III secretion system gatekeeper HrpJ	−73 **GGAACC**-N15-**CCACATA** −46
C1O30_RS11685		WP_102801846.1	Type III secretion protein HrpP	
C1O30_RS15015		WP_158653989.1	Hypothetical protein	
	JZL000137		Hypothetical protein	
	JZL000140		Hypothetical protein	
	JZL000450		Hypothetical protein	
	JZL001636		Hypothetical protein	
	JZL001650		Hypothetical protein	
	JZL001967		Hypothetical protein	
	JZL002766		Hypothetical protein	
	JZL002871		Hypothetical protein	
	JZL002874		Hypothetical protein	
	JZL002884		Hypothetical protein	

Previous studies in Er. amylovora, Pantoea stewartii, and Ps. syringae have characterized a conserved binding motif for HrpL, which is called the “*hrp* box” (GGAACC/T-N15/16-C/T/GCACNNA) (32, 33, 36). We analyzed the promoter sequences of all predicted T3SE genes and found the typical *hrp* box in six of them, including the known effector DspE (36), two harpins, HrpN and HrpW, secreted through the T3SS (31), two putative effectors, HrpZ and HrpK, and a T3SS gatekeeper, HrpJ (Table 2). Three other candidate T3SE genes, *C1O30_RS12170*, *C1O30_RS16815*, and *C1O30_RS19500*, encoding hypothetical proteins, also contain the *hrp* box in their promoters, except the distances between the two conserved modules are abnormally long (69, 56, and 37 nucleotides, respectively) (Table 2). Additionally, 400 ng of HrpL was demonstrated to bind to the promoter of *hrpA* encoding a T3SS substrate in *D*. *dadantii* 3937 with the help of core RNA polymerase (RNAP) (34). In MS2, HrpA was not predicted as a T3SE but contains a conserved *hrp* box in its promoter (GGAACC-N15-CTACTTA). To verify the affinity of the HrpL to the above-mentioned *hrp* box-containing promoters, we carried out electrophoretic mobility shift assays (EMSAs) and found that a high concentration (5 μg) of HrpL could directly bind to the promoters of *hrpA*, *hrpN*, *hrpZ*, *hrpK*, *dspE*, *hrpX*, *C1O30_RS12170*, *C1O30_RS16815*, and *C1O30_RS19500* in an *hrp* box-dependent manner without adding core RNAP *in vitro* (Fig. 8B). This is similar to the phenomenon observed in Bacillus subtilis primary sigma σ^A^, which by itself is able to interact with promoter DNA at the concentration of 10 μM without the assistance from core RNAP (37). The observed binding of HrpL in high concentrations to the promoters of *C1O30_RS12170*, *C1O30_RS16815*, and *C1O30_RS19500* containing unusual *hrp*-boxes in the absence of core RNAP is not likely to be biologically relevant or evidence of function, since these atypical *hrp*-boxes are not functional HrpL-dependent promoters, and the expression of these three genes is not significantly affected by HrpL (Fig. 8A). To verify this, low concentrations of HrpL in addition to RNAP were also used to test the affinity to these three promoters with atypical *hrp* boxes. No bound band was observed for the atypical promoters, while bound bands were formed by incubation of 0.2 μg HrpL protein with *hrpA* promoter, and 0.4 μg HrpL protein with *dspE* promoter, with RNAP (Fig. 8C).

### The expression of CWDE genes was broadly reduced in JZL7.

Plant cell wall is a major barrier for the invasion of pathogenic bacteria and is composed of mainly cellulose, hemicellulose, and pectin polymers. To breach this barrier, *D*. *zeae* bacteria produce a full set of cellulases (Cels), pectinases (Pels), polygalacturonases (Pehs), and proteases (Prts), along with a glucuronoxylanase (XynA) and two rhamnogalacturonanate lyases (RhiE1/2) (Table 3). Our previous study showed that JZL strains produced significantly smaller amounts of all four types of CWDEs compared to EC1 and MS2 when they were all cultivated at the same, relatively high cell density (optical density at 600 nm [OD_600_] = 1.8) (9).

**TABLE 3:** Genes encoding the CWDEs in genomes of Dickeya zeae

Gene	Accession no. of:				Identity between MS2 and JZL7
	EC1	Ech586	MS2	JZL7	
*celZ*	W909_12595	Dd586_1489	C1O30_RS13580	JZL002780	96.94
*celY*	W909_19635	Dd586_4057	C1O30_ RS20545	JZL004313	97.07
*bglA*	W909_01965	Dd586_0376	C1O30_ RS02015	JZL000410	97.56
*bgxA*	W909_07850	Dd586_2493	C1O30_ RS08765	JZL001697	95.72
*bglB*	W909_11710	Dd586_1660	C1O30_ RS12705	JZL002602	95.14
*nagZ*	W909_12020	Dd586_1600	C1O30_ RS13010	JZL002663	94.38
*bglC*	W909_16350			JZL003611	
*bglD*	W909_16355			JZL003612	
*celH*	W909_16395	Dd586_3407	C1O30_ RS17155	JZL003621	97.89
*lfaA*	W909_11990	Dd586_1606	C1O30_ RS12980	JZL002657	95.71
*pnl*	W909_02890		C1O30_ RS02835		
*pelN*	W909_08805	Dd586_2245	C1O30_ RS09885	JZL001947	97.72
*pelL*	W909_12600	Dd586_1488	C1O30_ RS13585	JZL002781	96.36
*pelI*	W909_14095	Dd586_2937	C1O30_ RS14580	JZL003127	84.96
*pelA*	W909_14860	Dd586_3083	C1O30_ RS15345	JZL003289	96.11
*pelE*	W909_14870	Dd586_3084	C1O30_ RS15350	JZL003291	96.50
*pelD*	W909_14875	Dd586_3085	C1O30_ RS15355	JZL003292	97.84
*pelC*	W909_18430	Dd586_3788	C1O30_ RS19205	JZL004051	96.63
*pelB*	W909_18435	Dd586_3789	C1O30_ RS19210	JZL004052	97.61
*pelZ*	W909_18440	Dd586_3790	C1O30_ RS19215	JZL004053	92.89
*pelW*	W909_10230	Dd586_1962	C1O30_ RS11330	JZL002317	98.16
*pelX*	W909_20195	Dd586_4161	C1O30_ RS21105	JZL004439	97.03
*paeX*	W909_10260	Dd586_1956	C1O30_ RS11360	JZL002324	95.61
*paeY*	W909_14880	Dd586_3086	C1O30_ RS15360	JZL003293	91.08
*pemA*	W909_14885	Dd586_3087	C1O30_ RS15365	JZL003294	97.07
*pehN*			C1O30_ RS05710	JZL001060	98.69
*pehK*	W909_15935	Dd586_3319	C1O30_ RS16710	JZL003524	96.93
*pehX*	W909_19025	Dd586_3904	C1O30_ RS19785	JZL004171	96.91
*rhiE1*			C1O30_ RS04480	JZL000839	98.00
*rhiE2*	W909_09610	Dd586_2097	C1O30_ RS10600	JZL002175	97.29
*xynA*	W909_10005	Dd586_2011	C1O30_ RS11050	JZL002266	94.12
*prtX*	W909_09760	Dd586_2059	C1O30_ RS10785	JZL002216	95.49
*prtC*	W909_09765	Dd586_2058	C1O30_ RS10790	JZL002217	97.71
*prtB*	W909_09770	Dd586_2057	C1O30_ RS10795	JZL002218	96.53
*prtG*	W909_09795	Dd586_2052	C1O30_ RS10820	JZL002223	96.30
*prtF*	W909_09775	Dd586_2056	C1O30_ RS10800	JZL002219	97.51
*prtE*	W909_09780	Dd586_2055	C1O30_ RS10805	JZL002220	97.09
*prtD*	W909_09785	Dd586_2054	C1O30_ RS10810	JZL002221	96.89

**FIG 9:**
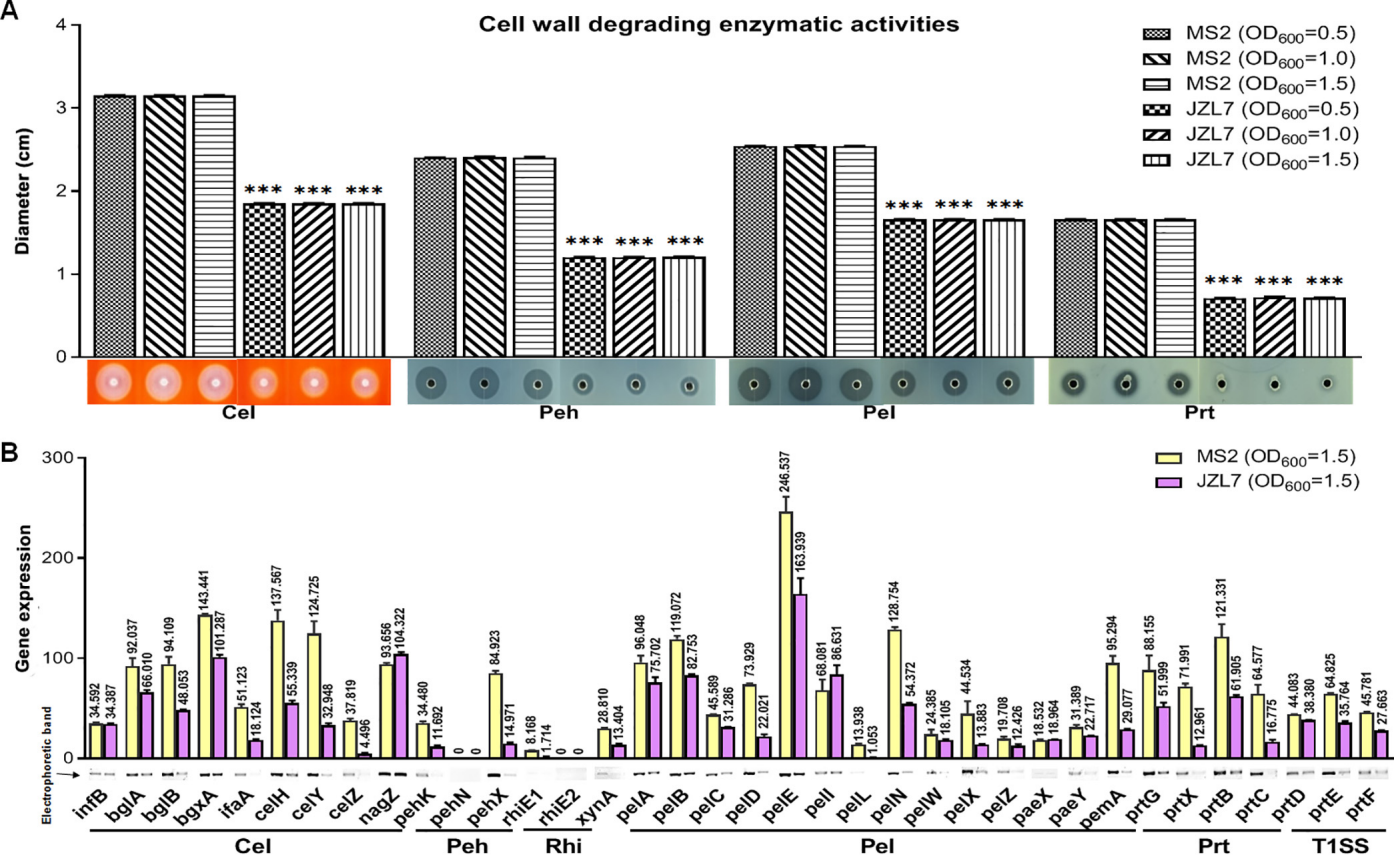
Extracellular cell wall-degrading enzymes (CWDEs) produced by *D*. *zeae* strains MS2 and JZL7. (A) CWDE activities of strains MS2 and JZL at different cell concentrations. Samples of 20 μL bacterial cells (OD_600_ of 0.5, 1.0 and 1.5) were added to the assay plate wells (5 mm in diameter) and incubated at 28°C. Pel and Peh assay plates were treated with 1 M HCl after 14 h, respectively. Cel assay plate was stained with 0.1% Congo red for 15 min after 14 h and decolored twice with 1 M NaCl. Photos were taken of Prt assay plate after 24 h without any further treatment. (B) RT-PCR of CWDE genes of strains MS2 and JZL (OD_600_ of 1.5). The reference gene *infB* (coding transfer initiation factor 2) was used to equilibrate the concentration of cDNA samples. The expression of genes was determined by measuring the signal intensity of the bands (under the *x* axis) using Image Lab software (Bio-Rad, USA). Experiments were repeated three times in triplicate, and the mean data above the bars indicate the signal intensity of RT-PCR bands.

In this study, we further compared the CWDE activities of strains JZL7 and MS2 at three different cell densities (i.e., OD_600_ of 0.5, 1.0, and 1.5) and observed a similar pattern that JZL7 produced a considerably smaller amount of CWDEs (Cels: 41.3%; Pehs: 50%; Pels: 34.6%; Prts: 57.2%) than MS2 at all densities (Fig. 9A). The lower CWDE activities of JZL7 might be due to either a much smaller repertoire of CWDE encoding genes or a greatly reduced expression of the same set of genes. Our genome comparison revealed a highly conserved set of 34 CWDE genes shared by both JZL7 and MS2 (84.96% to 98.69% DNA sequence identity) and very few strain-specific genes, including two beta-glucosidase-encoding genes (*bglC* and *bglD*) in JZL7 and one pectin lyase-encoding gene (*pnl*) in MS2 (Table 3). Interestingly, recent studies showed that *pnl* was drastically upregulated during early disease development in Pectobacterium carotovorum but not so in *D*. *dadantii* (38). Here, in *D*. *zeae*, we found that neither the complementation of *pnl_MS2_* in JZL7 nor the deletion of *pnl* in MS2 altered the virulence of respective strains on dicotyledonous tissues (i.e., potato and radish slices) (Fig. S2). We then measured the expression of the shared CWDE genes in the two strains using semiquantitative PCR. Results showed that the ratios (JZL7 versus MS2) of cumulative expression levels of genes encoding Cels, Pehs, RhiEs, XynA, Pels, and Prts are 0.56 (430.579:774.477), 0.22 (26.663:119.403), 0.21 (1.714:8.168), 0.47 (13.404:28.810), 0.62 (632.929:1,023.790), and 0.11 (143.640:1,346.054), respectively. In addition, *Dickeya* exports Prts via T1SS encoded by the *prtD*, *prtE*, and *prtF* genes after recognizing a C-terminal signal sequence in their substrates (18, 39, 40). The expression level of T1SS genes in strain JZL7 is 0.66 of those in strain MS2 (Fig. 9B). Furthermore, 15 of the 34 shared CWDE genes had significantly lower expression levels in JZL7 than in MS2, including *lfaA*, *celH*, *celY*, *celZ*, *pehK*, *pehX*, *rhiE1*, *xynA*, *pelD*, *pelL*, *pelN*, *pelX*, *pemA*, *prtX*, and *prtC* (Fig. 9B).

The broadly reduced expression of CWDE genes observed here was consistent with the aforementioned lower CWDE activities in JZL7 and may provide a basis for the strain’s lack of virulence on dicots. Specifically, monocots and dicots differ in the composition and structure of their primary cell walls; the former contain mainly cellulose and rarely pectin, while the latter consist of both (41). In this study, we showed that several pectin lyase-encoding genes were either lost (e.g., *pnl*) or expressed at remarkably lower levels (e.g., *pelD*, *pelL*, *pelN*, *pelX*, and *pemA*) in JZL7 (Fig. 9B). The resulting reduction in Pel activity might considerably compromise the ability of JZL7 to degrade cell wall and invade dicot hosts, which, together with the loss of T3SS, might abolish the pathogenicity of JZL7 on dicots. To verify whether the significantly lower expression of CWDEs is due to the lower expression of some important regulators controlling CWDE activity, we measured the expression of the genes encoding CWDE regulators, such as Fis, SlyA, VfmE, PecS, PecT, and KdgR, in both MS2 and JZL7 by RT-PCR. Results showed that none of these genes was differentially expressed between MS2 and JZL7 (Fig. S3).

### Other 13 genes were newly found to encode pathogenicity-related proteins in *D*. *zeae* MS2.

**FIG 10:**
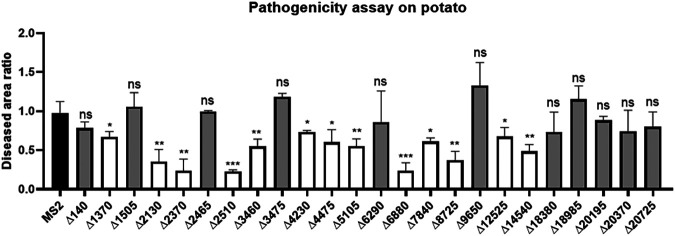
Maceration tests of other *D*. *zeae* MS2-unique gene mutants on potato slices. The data present the means of three replicates, and error bars represent the standard deviation. ns, not significant; *, *P* < 0.05; **, *P* < 0.01; ***, *P* < 0.001; ****, *P* < 0.0001 (Student’s *t* test).

In order to identify additional factors related to the virulence of *D*. *zeae* pathogens on dicots, we obtained knockout mutants in MS2 for most of the genes absent in JZL7 but shared by MS2 and EC1 (apart from the T3SS and T3SE genes, which have already been examined above; Table S8). Pathogenicity tests showed that deletion of *C1O30_RS01370-01390*, *C1O30_RS02130*, *C1O30_RS02370*, *C1O30_RS02510*, *C1O30_RS03460*, *C1O30_RS04230*, *C1O30_RS04475*, *C1O30_RS05105*, *C1O30_RS06880*, *C1O30_RS07840*, *C1O30_RS08725*, *C1O30_RS12525*, and *C1O30_RS14540* significantly reduced the virulence of MS2 on potato slices (Fig. 10). Among them, *C1O30_RS01370-1390*, *C1O30_RS02510*, *C1O30_RS03460*, *C1O30_RS04230*, *C1O30_RS04475*, *C1O30_RS05105*, *C1O30_RS07840*, *C1O30_RS08725*, *C1O30_RS12525*, and *C1O30_RS14540*, respectively, carry a dimethyl sulfoxide reductase anchor subunit gene cluster (*dmsC*, *dmsB*, *dmsA*, *dmsD*, and a SDR family oxidoreductase) and encode a GNAT family N-acetyltransferase, LysE family transporter, a PAS domain-containing protein (helix-turn-helix transcriptional regulator), an SDR family oxidoreductase, an HAMP domain-containing protein, an FMN-binding negative transcriptional regulator, a glycosyltransferase, DUF2335 domain-containing protein, and a diguanylate cyclase, while all the others encode hypothetical proteins (Table S8). All these genes were first reported to be involved in *Dickeya* pathogenesis and, thus, represent prominent targets of future studies.

Recently, an *saxA* gene encoding isothiocyanate hydrolase was found to enable a potato-specific Pectobacterium parmentieri strain to increase the ability to macerate *Arabidopsis*, suggesting its significant role in defining the host range (42). We searched the homologs of this interesting gene in the genomes of *D*. *zeae* and found 4 and 6 copies of *saxA*, respectively, present in the genomes of MS2 and JZL7. Thus, we ruled out the possibility of SaxA as a host range determinant.

### Pathogenic mechanism of *Dickeya* and other soft rot *Pectobacteriaceae*.

In this study, we sequenced the genome of *D*. *zeae* JZL7 and carried out in-depth comparative and functional analyses with other *D*. *zeae* strains. The results not only provide insights into the unique pathogenicity and host specificity of JZL7 but also help to better understand the pathogenic mechanism of soft rot *Pectobacteriaceae* pathogens (e.g, *Dickeya*, *Erwinia*, and *Pectobacterium*) in general.

*Xanthomonas*, *Ps*. *syringae*, and many other bacterial phytopathogens adopt the “stealth” model of pathogenicity whereby they make use of an extensive battery of type III secreted effector proteins and phytotoxins for successful infection. These effectors act to suppress or manipulate host defenses as the bacterial population grows to numbers that are sufficient to induce disease symptoms (43–47). Additionally, many studies have demonstrated that T3SEs could alter the physiology of host cells in a way that is beneficial for the pathogens. In *Xanthomonas*, a mutation in the T3SS impairs the ability to inject T3SEs in the host plant and, as a consequence, abolishes pathogenicity and multiplication *in planta* (48). Among pathogenicity determinants shown to display heterogeneous distribution between strains are T3SEs. Furthermore, in *Xanthomonas* and *Ps*. *syringae*, diversity in T3SE repertoires from different hosts revealed determinants of host specificity (45, 49, 50).

Soft rot *Pectobacteriaceae* pathogens also possess in their genomes the molecular machineries required for the stealth mode of infection, such as T3SS, T4SS, phytotoxins, and so on. Functional studies have also demonstrated the importance of T3SS in soft rot *Pectobacteriaceae* (SRP) pathogens. For instance, T3SS is important for the full virulence of *D*. *dadantii* 3937 (25, 26) and required for pellicle formation and cell aggregation (51, 52). However, T3SS or T3SE has not been reported in SRP as a determinant of host range, and the role of T3SS in the virulence of necrotrophic SRP and other bacterial pathogens appears quite complex.

In our study, we knocked out the whole T3SS gene clusters *dsp/hrp/hrc* in *D*. *zeae* MS2, which resulted in significantly reduced virulence on both monocotyledonous and dicotyledonous hosts (Fig. 7), demonstrating its role as an important virulence factor in the course of disease. However, Δ*dsp*/*hrp*/*hrc* was not virulence free on either monocots or dicots, suggesting that T3SS alone does not determine host range in *D*. *zeae*. In addition, *Er*. *pyrifoliae*, *Pe*. *carotovorum*, Pectobacterium wasabiae, and *Ps*. *syringae* strains lacking a functional T3SS have been reported to infect plants (22, 23, 53, 54). In *Ps*. *syringae*, nonpathogenic isolates are separate from the pathogenic ones due to the deficiency of T3SS and T3SEs (23). In contrast, T3SS-deficient *Pe*. *wasabiae* strains are still virulent (53). More strikingly, human-pathogenic *Ps*. *aeruginosa* isolates from patients with chronic lung infections are typically T3SS deficient, even though 90% of environmental isolates carry a T3SS (55). These T3SS-deficient pathogens may have alternative ways of modifying the host plant at the initiation of infection.

It has often been assumed that the mechanisms of infection used by SRP are distinctly different from those used by other bacteria like *Xanthomonas* and *Ps*. *syringae* (56). In addition to the above-mentioned stealth model, SRP pathogens also share a “brute-force” model of pathogenicity; they produce many CWDEs to physically attack the plant cell walls and their surrounding apoplast, which thus promotes soft rotting (45, 57). It is unquestionable that brute force has made the SRP highly successful pathogens, and the CWDEs are critical factors in both the pathogenicity and host range of SRP. We compared the concentrated enzymatic activities of soft rot *D*. *zeae* MS2 and JZL7 and found that broadly reduced expression of CWDE genes might be a major cause for the significantly lower virulence of JZL7 on dicots (Fig. 9). Importantly, the brute-force and stealth models seem to operate in parallel in SRP pathogens, as the production of CWDEs remained unaffected in the T3SS mutants of MS2 (Fig. S4), which might explain their remaining soft rot symptoms on potato and radish slices (Fig. 7C and D).

Bacterial phytopathogens employ various approaches to infect and kill their host(s). Plant-pathogen interaction is a multifaceted process, mediated by the pathogen- and plant-derived molecules. Secreted as well as translocated molecules, derived from the pathogens, are the key factors, which determine their pathogenicity and allow successful colonization of pathogens on the host. On the other hand, plant-derived molecules are involved in the recognition of pathogen and triggering of the defense response. In the future, to learn more about the latent stage of *Dickeya* infection and host range, we should consider factors including both CWDEs and T3SEs.

## MATERIALS AND METHODS

### Bacterial strains and growth conditions.

The bacterial strains and plasmids used in this study are listed in Table S1. Escherichia coli strains were grown at 37°C in Luria-Bertani (LB) medium. *D*. *zeae* and its derivatives strains were cultivated at 28°C in LB medium, minimal medium (MM), or LS5 medium (58, 59). Antibiotics were added to the media at the following final concentrations when required: streptomycin 50 µg/mL, kanamycin 50 µg/mL, tetracycline 15 μg/mL, and ampicillin 100 μg/mL.

### Genome sequencing and assembly.

Genomic DNA was extracted from *D*. *zeae* strain JZL7 in LB medium culture using MasterPure DNA purification kit (EPICENTRE Biotechnologies, Madison), which was then subjected to quality control by agarose gel electrophoresis and quantified by Qubit. The genome sequencing was performed at Health Time Gene (Shenzhen, China) using both the PacBio RS II and the Illumina Hiseq X Ten platforms.

For the PacBio sequencing, genomic DNA was treated into fragments in 10 kb by g-TUBE first. The fragments were damage-repaired and end-repaired. Both sides of the DNA fragments were, respectively, connected with hairpin adapter to get a dumbbell structure, which is known as SMRTbell. After annealing, the SMRTbell was fixed at the bottom of the ZWM polymerase and was sequenced last. Adapter trimming and quality filtering were performed by using fastp (version 0.20.0) (60) with default parameters.

For the Illumina sequencing, genomic DNA was randomly broken, and 350 bp fragments were purified and end-repaired using T4 DNA polymerase, Klenow DNA polymerase, and T4 PNK. The fragments were added with an “A” base at the 3′ terminal and ligated with the adaptor with a “T” base at its terminal. The library was sequenced on the Hiseq X Ten platform to generate PE150 reads. A hybrid genome assembly with both the PacBio long-reads and Illumina short-reads was carried out using Unicycler v0.4.7 (default parameters) (61).

### Genome annotation.

The genome annotation of JZL7 was carried out using the NCBI Prokaryotic Genome Annotation Pipeline (PGAP) v2019-11-25 (standalone version) (62). Functional annotations of all proteins predicted in JZL7 were conducted using eggNOG-mapper v1.0.3 (for GO) (63), KofamKOALA v2020-08-04 (for KEGG) (64), and COGsoft v201204 (for COG) (65). The genome and annotated protein sequences of other *Dickeya* strains analyzed in this study were all obtained from the NCBI RefSeq and GenBank databases (Table S2). Genomic islands and prophages were predicted using the webservers of IslandViewer 4 (http://www.pathogenomics.sfu.ca/islandviewer/) (66) and PHASTER (http://phaster.ca/) (67), respectively. Genes encoding type I to VI secretion system-related proteins were identified using TXSScan v1.0.2 (68). Type III secreted effectors (T3SEs) were predicted by using a combination of four bioinformatics approaches, including the webserver of Bastion3 (https://bastion3.erc.monash.edu/) (69) and standalone versions of BEAN2 v2.0 (70), DeepT3 (71), and pEffect (72); a protein is classified as T3SE only if it is predicted by at least three of the four approaches. The circular visualization of JZL7 genome characteristics was created by Circos (73). Prediction of *hrp* box was performed by searching the promoter sequences (2,000 bp upstream the start codon) of the candidate T3SE-coding genes in MS2 genome using the following regular expressions: GGAAC[CT][ATCG]{1,100}[CGT][CT]AC[ATG][CT]A (forward) and T[AG][ATC]GT[AG][AG][AGCT]{1,100}[AG]GTTCC (reverse).

### Comparative genomic analyses.

Pairwise average nucleotide identity (ANI) values between all *D*. *zeae* and *D*. *oryzae* strains were calculated using pyani v0.2.10 (with the “-m ANIb” option) (74). The whole-genome alignment of CE1, EC1, EC2, Ech586, JZL7, and MS2 was constructed using Mauve v2.4.0 (75). The pangenome analysis of EC1, JZL7, and MS2 was carried out using OrthoFinder v2.4.0 (76). To build the phylogenetic tree, we generated multiple DNA sequence alignment for each of the 3,110 single-copy core genes (present in all strains) using MAFFT v7.453 (77). All single-gene alignments were then concatenated, and a phylogenetic inference was conducted using IQ-TREE v1.6.12 (78). Reliability of the inferred topology was assessed by ultrafast bootstrap with 1,000 replicates.

### Generation of in-frame deletion mutants.

For generation of in-frame deletion mutants of *D*. *zeae* MS2, triparental mating was performed using the methods described previously (15). For instance, to knock out the *hrp*/*hrc* gene cluster of T3SS in strain MS2, the upstream and downstream fragments of *hrp*/*hrc* gene cluster were amplified using the primer pairs of hrp/hrc-1 accompanied with hrp/hrc-2, and hrp/hrc-3 accompanied with hrp/hrc-4, respectively (Table S3), and purified with AxyPrep DNA gel extraction kit (Axygen Biotech Co., Hangzhou, China). The fragments were ligated with the *Bam*HI and Spe digested suicide plasmid pKNG101 using ClonExpress MultiS kit (Vazyme Biotech Co., Nanjing, China) and transformed into E. coli CC118 competent cells. In triparental mating, the donor and receptor cells were mixed with the helper strain E. coli RK2013 (grown in LB medium containing kanamycin) in a ratio of 2:1:1 on LB plate and incubated at 28°C overnight. The transformants were grown on minimal medium (MM) agar plates (13) containing streptomycin sulfate. Single colony culture was then spread on MM agar plate containing 5% sucrose to exclude the suicide plasmid. The resultant deletion mutants were confirmed by PCR using the detection primer pair of hrp/hrc-F and hrp/hrc-R and DNA sequencing. In the same way, we correspondingly deleted the important T3SS transcriptional regulation genes *hrpX*, *hrpY*, *hrpS*, and *hrpL* and the candidate T3SE genes *dspE*, *hrpZ*, *hrpW*, *hrpK*, and *hrpN*, the pectin lyase-encoding gene *pnl*, and, compared to JZL7, some genes specific to MS2. In addition, the Δ*dsp*/*hrp*/*hrc* mutant was also generated by deleting the *dsp* gene cluster based on the obtained Δ*hrp*/*hrc* mutant. The primers are listed in Table S3.

### Measurement of bacterial growth curves.

Bacterial strains were grown in LB medium overnight at 28°C, adjusted to an OD_600_ of 1.5, and diluted into fresh LB and LS5 medium in a 1:100 ratio, respectively (9). Aliquots of 500-μL dilutions were transferred into 2.0-mL tubes. Bacteria were grown with shaking at 200 rpm under 28°C, and cell density was measured every 2 h. The experiment was repeated three times in triplicate.

### HR assay.

Bacteria were grown in LS5 medium (pH 5.5) (59) until they reached an OD_600_ of 1.0. Leaves of Nicotiana tabacum variant K326 (27) and N. benthamiana (28) were, respectively, inoculated on the back by pressing a sterilized puncher (5 mm) dipped with bacterial culture and incubated at 28°C. The area of HR lesions on leaves was measured using Image J 1.52a 12 and 24 h postinoculation (hpi) (79). To quantify the function of the T3SS, we infiltrated leaves of N. tabacum K326 with 100 μL of MS2 bacterial dilutions in LS5 medium (OD_600_ of 0.5, containing approximately 10^8^ CFU/mL), and 100 μL each of LS5 medium, Δ*dsp*/*hrp*/*hrc*, and JZL7 (10^7^ CFU/mL) was also infiltrated as a negative control. Each assay was repeated three times in triplicate.

### Pathogenicity tests on monocotyledonous and dicotyledonous plants.

Strains were cultured to the logarithmic phase until an OD_600_ of 1.2 was reached in LB medium, and pathogenicity tests were carried out on monocotyledonous and dicotyledonous hosts. For monocots, every 100 μL of bacterial culture was injected into the basal stem of banana and the base of clivia leaf. For dicotyledonous plants, potato and radish tubers were surface-sterilized with 70% ethanol, cut evenly about 5 mm in thickness, and then placed onto moistened filter paper in a tray. Bacterial cells of 2 μL were applied to the center of the tuber slices. All plants were kept in a growth chamber with controlled conditions of 28 ± 2°C, 75% ± 15% relative humidity, and 24 h white light (7,350 lx) illumination until symptoms appeared, except that the bananas were incubated at 30°C with 12-h alternating light-dark cycles for 7 days and then disease was assessed using a modified virulence scoring (17, 80). Same volume of LB medium was inoculated as a negative control. Visible macerate areas on clivia leaves and potato and radish slices were measured using Image J 1.52a. Each assay was repeated three times in triplicate.

### RNA purification.

The bacterial cultures used for RNA extraction were the ones described below for CWDE activity measurement. Since the same strain shared basically the same enzymatic activity in different cell densities (OD_600_ of 0.5, 1.0, and 1.5), the bacterial cultures of strains MS2 and JZL7 at an OD_600_ of 1.5 were selected for RNA extraction. On the other hand, to determine whether the predicted T3SE expression is induced by the HrpL regulator, we grew strains MS2 and Δ*hrpL* in LS5 medium (pH 5.5) (59) for RNA extraction until an OD_600_ of 1.0 was reached. The RNA was extracted using the SV total RNA isolated system kit (Promega, Madison, WI, USA), further purified using the RNA clean kit (Qiagen, Hilden, Germany), and treated with DNase I to degrade any possible DNA contamination. Quantity of RNA was first measured using a NanoDrop 2000c (Thermo Fisher Scientific, MA, USA), and the integrity of RNA was detected by agarose gel electrophoresis.

### RT-PCR analysis.

For each RNA sample for CWDE activity measurement, two dilutions (5 and 50 ng) were reverse transcribed into cDNAs using FastKing gDNA dispelling RT mix (Tiangen Biotech, Co., Ltd., Beijing, China), and the concentration of each resultant cDNA was quantitatively equilibrated according to the expression quantities of reference gene *infB* of strains MS2 and JZL7. PCR was performed using the primers listed in Table S3, and gene expression (signal intensity) of each gene was determined using the software Image Lab (Bio-Rad, USA). The experiment was repeated three times in triplicate.

### qRT-PCR analysis.

In qRT-PCR analysis, an aliquot of 300 ng RNA sample was used for genomic DNA elimination and cDNA synthesis by a FastKing RT kit (with gDNase) (Tiangen Biotech, Co., Ltd., Beijing, China) following the manufacturer’s protocol. The housekeeping gene *atpD* was used as a reference. The PCR efficiency of each gene was determined using five DNA standards at different concentrations (10, 1, 0.1, 0.01, and 0.001 g/mL). The qRT-PCR was conducted on a Quantstudio 6 Flex system using ChamQ Universal SYBR qPCR master mix (Vazyme Biotech Co., Nanjing, China) with the following cycle profile: 1 cycle at 95°C for 30 s, followed by 40 cycles at 95°C for 10 s, 60°C for 30 s, and then 1 cycle at 95°C for 15 s, 60°C for 60 s, and 95°C for 15 s. The experiment was repeated three times, each time in triplicate. Data were analyzed using the 2^−ΔΔCT^ method as described previously (81).

### Protein expression and purification.

The open reading frame (ORF) that encodes HrpL protein was amplified from MS2 genomic DNA using primers pET32a-hrpL-F and pET32a-hrpL-R containing *Bam*HI and *Hind*III restriction enzyme sites (Table S3). The PCR product was purified and ligated to the *Bam*HI/*Hind*III-digested pET32a vector harboring a thioredoxin (TRX)-His_6_ tag at its N terminus. pET32a-hrpL was then transformed into the E. coli strain BL21(DE3) competent cells (TransGen Biotech Co., Beijing, China) and confirmed by sequencing. To express the HrpL protein, a single colony of BL21(pET32a-hrpL) was grown in LB medium containing 100 μg/μL of ampicillin overnight and transferred into 1 L of fresh LB medium in a 1:100 ratio to grow until reaching an OD_600_ of 0.6. Then, 1 mM IPTG (isopropyl β-d-1-thiogalactopyranoside) was added to the culture to induce protein expression at 18°C for 16 h. Bacterial cells were harvested by centrifuging at 5,000 rpm at 4°C for 30 min and suspended in 20 mL of phosphate-buffered saline (PBS). The cells were lysed with sonication and centrifuged at 12,000 rpm at 4°C for 30 min. The supernatant was filtered with a 0.45-μm-pore filter (MilliporeSigma, St. Louis, MO, USA), and the filtrate was added into a Ni-nitrilotriacetic acid (NTA) column (Changzhou Smart-Life Sciences Biotechnology Co., Changzhou, China) that had been equilibrated with lysis buffer. After washing five times with lysis buffer, the column was eluted with 30 mL gradients of 10 to 500 mM imidazole prepared in wash buffer, respectively. Fractions were collected, and sodium dodecyl sulfate-polyacrylamide gel electrophoresis (SDS-PAGE) was used to verify the molecular weight of the target protein. The protein was concentrated by centrifugation (MilliporeSigma, St. Louis, MO, USA) at 4°C and then stored at −80°C.

### Electrophoretic mobility shift assay.

Electrophoretic mobility shift assay (EMSA) was performed as described previously (58). DNA probes were PCR amplified using primers listed in Table S3. The purified PCR fragments were labeled by biotin using the biotin 3′ end DNA labeling kit (Thermo Fisher Scientific, MA, USA). Binding reactions were performed in a final volume of 10 μL using LightShift chemiluminescent EMSA kit following the manufacturer’s protocol (Thermo Fisher Scientific, MA, USA). For each reaction, 1 μL of 10× binding buffer, 0.5 μL of 1 μg/μL Poly, 1 nmol of labeled DNA fragments, and 1, 5, or 10 μg of HrpL protein placed in an ice bath were added together and incubated for 30 min at 20°C. The protein-DNA complexes and the unbound free DNA fragments were separated on 6% nondenaturing polyacrylamide (acrylamide/bisacrylamide 29: 1 [vol/vol]) gels using the electrophoresis buffer Tris-borate-EDTA (TBE) and were detected by chemiluminescence (Tanon, China). A 100-fold molar excess of unlabeled DNA fragment was incubated with HrpL protein for 15 min before addition of the labeled DNA fragments to verify specific interaction of the HrpL protein-DNA fragment. In the experiment of adding core RNA polymerase (RNAP), E. coli core RNAP (0.5 U) (New England Biolabs [Beijing] Ltd., Beijing, China) (82) was incubated on ice for 20 min with 200 or 400 ng of purified HrpL protein (34). The remaining steps were as described above.

### Measurement of CWDE activities.

The activities of CWDEs were measured according to the methods described previously (9). Specifically, pectate lyase (Pel) assay medium (10 g/L polygalacturonic acid, 10 g/L yeast extract, 8 g/L agarose, 0.38 μM CaCl_2_, and 100 mM Tris-HCl [pH 8.5]), polygalacturonase (Peh) assay medium (5 g/L polygalacturonic acid, 2 g/L sucrose, 2 g/L (NH4)_2_SO_4_, and 15 g/L agar [pH 5.5]), cellulase (Cel) assay medium (1.0 g/L carboxymethyl ethyl cellulose, 3.8 g/L Na_3_PO_4_, and 8.0 g/L agarose [pH 7.0]), and protease (Prt) assay medium (10 g/L skimmed milk, 5 g/L Bacto tryptone, 2.5 g/L yeast extract, 5 g/L NaCl, and 15 g/L agar) were prepared, and 30 mL of each medium was added into a 10 by 10 cm square plate. Wells in 5 mm diameter were made, and 20 µL of bacterial cells (OD_600_ of 0.5, 1.0, or 1.5) was applied to the wells. Plates were incubated at 28°C until Pel and Peh assay plates were treated with 1 M HCl after 14 h, and Cel assay plate was stained with 0.1% Congo red for 15 min after 14 h and decolored with 1 M NaCl twice. The protease activity was measured without any further treatment after 24 h. The experiment was repeated three times with duplicates.

### Introduction of *pnl* gene in strain JZL7.

To create the complementing plasmid, sequence of *pnI* open reading frame (ORF) with 19 bp before the start codon harboring a ribosome-binding site (RBS) was amplified and cloned from strain MS2 into the *Eco*RI/*Bam*HI-digested pLAFR3 vector using pEASY-Uni seamless cloning and assembly kit (TransGen Biotech Co., Beijing, China) and transformed into E. coli DH5α competent cells. Plasmid construct was confirmed by DNA sequencing and introduced into strain JZL7 by conjugal triparental mating. The primers used here are listed in Table S3.

### Statistical analysis.

All the experiments were repeated three times in duplicate or triplicate. GraphPad Prism 8.4.1 (GraphPad Software, San Diego, California) was used to performed unpaired two-tailed *t* test (83), and the data of *D*. *zeae* strain JZL7 were normalized to those of strain MS2. * indicates *P < *0.05, ** indicates *P < *0.01, *** indicates *P < *0.001, and **** indicates *P < *0.0001. Analysis of variance (ANOVA) was used between *D*. *zeae* MS2 and its mutants. The values are means of three replicates, and the error bars are standard deviations. Statistical analysis was performed on each group of data, and significantly different values (ANOVA *P < *0.05) are indicated by different letters.

To investigate association between the frequency of fold change (>0.5/≤0.5) and that of unique/shared T3SEs of MS2, the Pearson χ^2^ statistic was computed (Pearson’s chi-square test, χ^2^ = 8.77, degrees of freedom [df] = 1, χ^2^_0.995_ = 7.88, χ^2^_0.999_ = 10.83, and χ^2^_0.995_ < χ^2^ < χ^2^_0.999_). The *P* value was less than 0.005.

### Data availability.

Both the original genome sequencing data and the genome assembly were deposited in the NCBI Sequence Read Archive (SRA) database under the accession number PRJNA656647. The JZL7 genome sequence has been deposited in GenBank under accession number CP060263.1.
