# Supplementary material for: Genomic and Functional Dissections of Dickeya zeae Shed Light on the Role of Type III Secretion System and Cell Wall-Degrading Enzymes to Host Range and Virulence
Source: Microbiol Spectr. 2022 Feb 2;10(1):e01590-21. doi: 10.1128/spectrum.01590-21 (PMC8809351; doi:10.1128/spectrum.01590-21)
Supplement: SUPPLEMENTAL FILE 1 — Supplemental material. Download SPECTRUM01590-21_Supp_1_seq13.pdf, PDF file, 1.5 MB [file spectrum01590-21_supp_1_seq13.pdf]

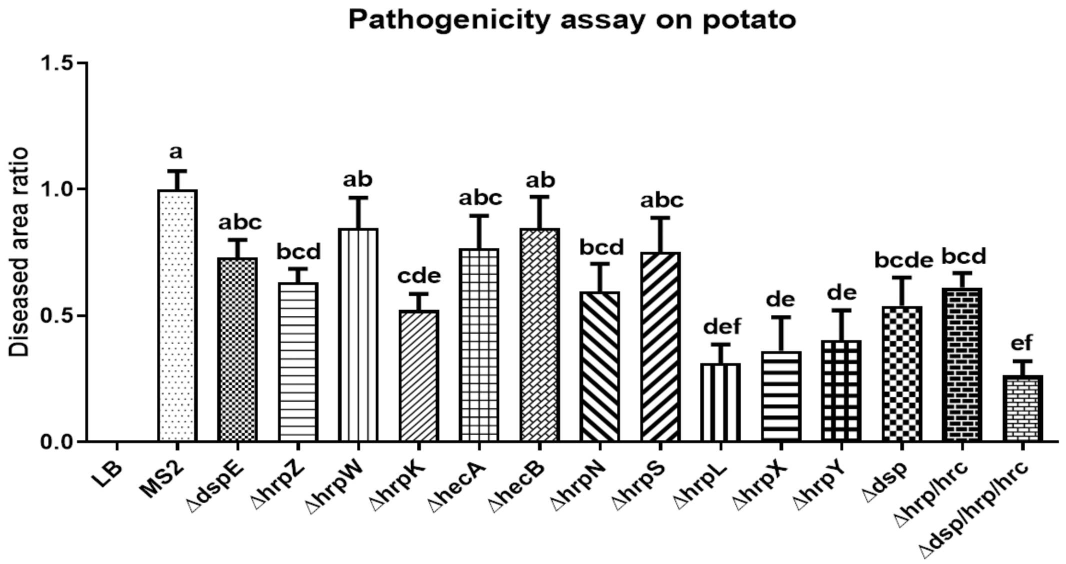

Fig. S1. Maceration tests of *D. zeae* MS2 T3SS mutants on potato slices. Potatos were washed with tap water and dried on tissue, which were sliced uniformly about 5 mm in thickness and washed three times with sterilized water. After drying on sterilized filter paper, the slices were transferred onto dishes containing three wet filter paper, and 2 μL of bacterial culture (OD_{600} = 1.2) was spotted onto the centre of the slices, and then incubated at 28 ^{o}C for 24 h till symptom developed. Visible macerate area was measured using Image J 1.52a. The data present the means of three replicates and error bars represent the standard deviation. Statistical analysis was performed on each group of data and significantly different values (ANOVA, $p<0.05$) are indicated by different letters.

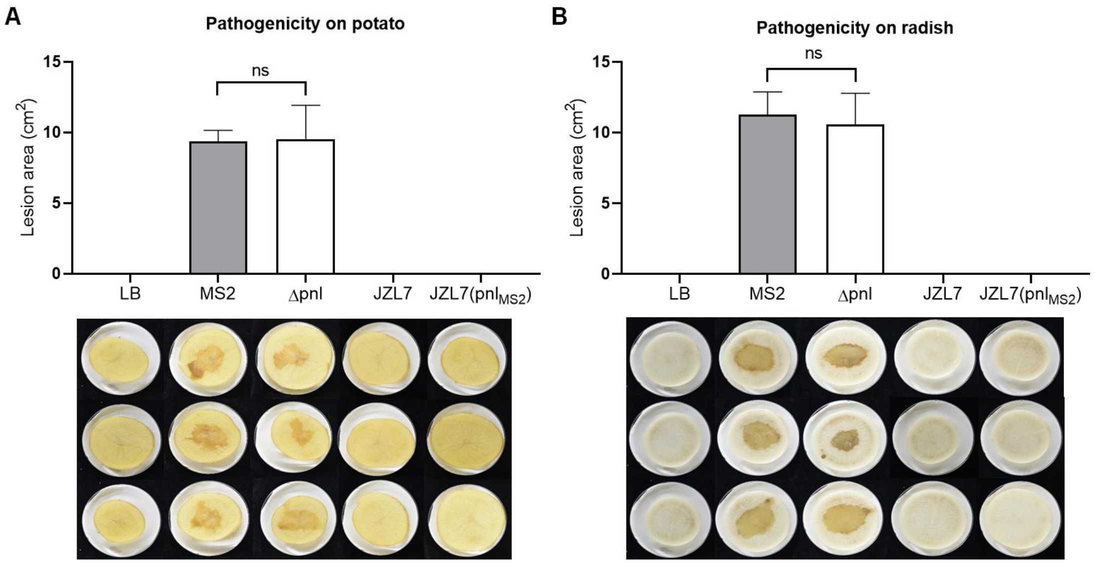

Fig. S2. Maceration tests of *D. zeae* MS2 *pnl* mutant and JZL7(pnl_{MS2}) on potato and radish slices. The data present the means of three replicates and error bars represent the standard deviation. "ns" indicates not significant (Student's *t*-test).

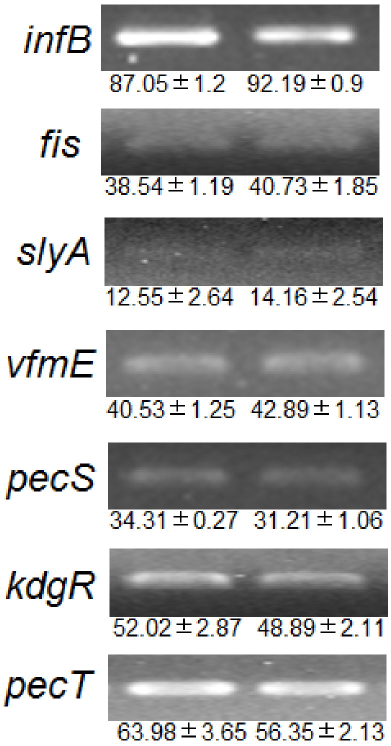

Fig. S3. RT-PCR analysis of the genes regulating CWDE production of strains MS2 and JZL (OD_{600} = 1.5). The reference gene *infB* (coding transfer initiation factor 2) was used to equlibrate the concentration of cDNA samples. The expression of genes was determined by measuring the signal intensity of the bands (under the X-axis) using Image Lab software (Bio-Rad, USA). Experiments were repeated three times in triplicates and the mean data above the bars indicated the signal intensity of RT-PCR bands.

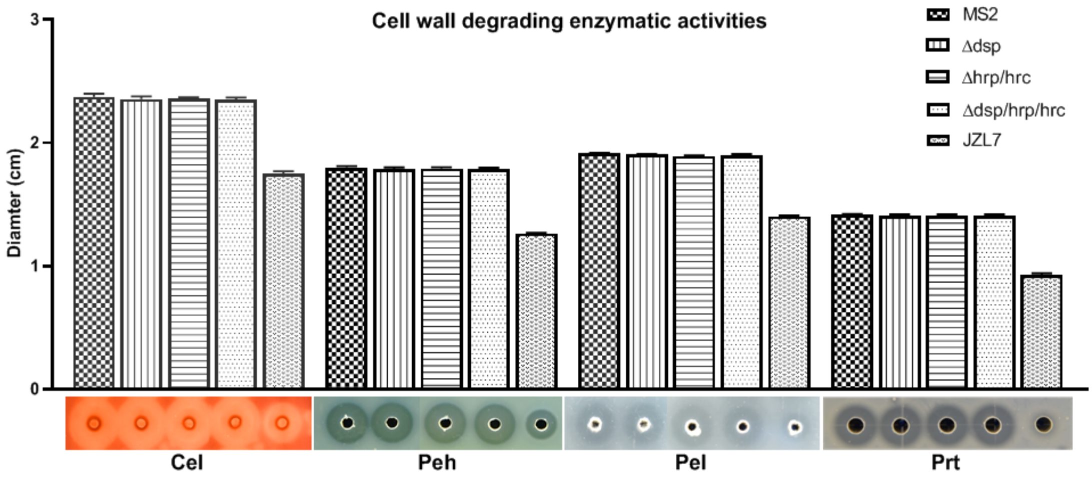

Fig. S4. Extracellular cell wall degrading enzymes (CWDEs) produced by *D. zeae* MS2, T3SS mutants and JZL7. The data present the means of three replicates and error bars represent the standard deviation.

Table S1. Bacterial strains and plasmids used in this study

**Strains or plasmids**	**Description**	**Source or reference**
***Dickeya* spp.**		
Ech586	Harboring T1SS, T2SS, T3SS, T4SS-2, T5SS, and T6SS; isolated from *Philodendron*; HR or host range unknown	Pritchard et al., 2012
EC1	Harboring T1SS, T2SS, T3SS, T4SS-2, T5SS, and T6SS; HR positive; isolated from rice, infective to both monocots and dicots	Hu et al., 2018
MS2	Harboring T1SS, T2SS, T3SS, T4SS-2, T5SS, and T6SS; HR positive; isolated from banana, infective to both monocots and dicots	Hu *et al.*, 2018
JZL7	Harboring T1SS, T2SS, T4SS-1, T4SS-2, T5SS, and T6SS; HR negative; isolated from soft rot *Clivia miniate*, infective to monocots but not dicots	Hu *et al.*, 2018
CE1	Harboring T1SS, T2SS, T5SS, and T6SS; isolated from *Canna edulis*; HR or host range unknown	Zhang et al., 2020
EC2	Harboring T1SS, T2SS, and T6SS; isolated from rice; HR or host range unknown	Zhang et al., 2020
Δdsp/hrp/hrc	*dsp*/*hrp*/*hrc* knock-out mutant of MS2	This study
Δhrp/hrc	*hrp*/*hrc* knock-out mutant of MS2	This study
Δdsp	*dsp* knock-out mutant of MS2	This study
ΔdspE	*dspE* knock-out mutant of MS2	This study
ΔhrpZ	*hrpZ* knock-out mutant of MS2	This study
ΔhrpW	*hrpW* knock-out mutant of MS2	This study
ΔhrpK	*hrpK* knock-out mutant of MS2	This study
ΔhrpN	*hrpN* knock-out mutant of MS2	This study
ΔhrpS	*hrpS* knock-out mutant of MS2	This study
ΔhrpL	*hrpL* knock-out mutant of MS2	This study
ΔhrpX	*hrpX* knock-out mutant of MS2	This study
ΔhrpY	*hrpY* knock-out mutant of MS2	This study
Δpnl	*pnl* knock-out mutant of MS2	This study
Δ140	*C1O30_RS00140 - 00155* knock-out mutant of MS2	This study
Δ1370	*C1O30_RS01370 - 01390* knock-out mutant of MS2	This study
Δ1505	*C1O30_RS01505* knock-out mutant of MS2	This study
Δ2130	*C1O30_RS02130* knock-out mutant of MS2	This study

Δ2370	*C1O30_RS02370* knock-out mutant of MS2	This study
Δ2465	*C1O30_RS02465* knock-out mutant of MS2	This study
Δ2510	*C1O30_RS02510* knock-out mutant of MS2	This study
Δ3460	*C1O30_RS03460* knock-out mutant of MS2	This study
Δ3475	*C1O30_RS03475* knock-out mutant of MS2	This study
Δ4230	*C1O30_RS04230* knock-out mutant of MS2	This study
Δ4475	*C1O30_RS04475* knock-out mutant of MS2	This study
Δ5105	*C1O30_RS05105* knock-out mutant of MS2	This study
Δ6290	*C1O30_RS06290* knock-out mutant of MS2	This study
Δ6880	*C1O30_RS06880* knock-out mutant of MS2	This study
Δ7840	*C1O30_RS07840* knock-out mutant of MS2	This study
Δ8725	*C1O30_RS08725* knock-out mutant of MS2	This study
Δ9650	*C1O30_RS09650* knock-out mutant of MS2	This study
Δ12525	*C1O30_RS12525* knock-out mutant of MS2	This study
Δ13675	*C1O30_RS13675* knock-out mutant of MS2	This study
Δ14540	*C1O30_RS14540* knock-out mutant of MS2	This study
Δ18380	*C1O30_RS18380* knock-out mutant of MS2	This study
Δ18985	*C1O30_RS18965 - 18985* knock-out mutant of MS2	This study
Δ20195	*C1O30_RS20195* knock-out mutant of MS2	This study
Δ20370	*C1O30_RS20370* knock-out mutant of MS2	This study
Δ20725	*C1O30_RS20725* knock-out mutant of MS2	This study
JZL7(pnl_{MS2})	Wild-type JZL7 containing the coding region from *pnl* of MS2 at down-stream of pTAC promoter, Tc^r	This study
Escherichia coli		
CC118λ	Δ*(ara-leu) araD* Δ*lacX74 galE galK phoA20 thi-1 rpsE rpoB argE* (Am) *recA1, λ pir*, *Escherichia coli* strain as host for plasmids constructs derived from pKNG101	Lab collection
HB101(pRK2013)	*Thr leu thi recA hsdR hsdM pro*, Kan^r	Lab collection
BL21 (DE3)	*E. coli str. B F– ompT gal dcm lon hsdSB(rB–mB–) λ(DE3 [lacI lacUV5-T7p07 ind1 sam7 nin5]) [malB+]K-12(λS)*	Lab collection

**Plasmids**		
pKNG101	Knockout vector, Str ^{r}	Lab collection
pRK2013	Tra^{+}, Mob^{-}, ColE1-replicon, Kan^{r}, Spe^{r}	Lab collection
pET-32a (+)	Overexpression and purification vector, Ap^{r}	Lab collection
pET-32a-hprL	HrpL protein expression vector, Ap^{r}	This study
pLAFR3	broad-host-range cosmid cloning vector, Tc^{r}	Lab collection
pLAFR3-pnl	pLAFR3 expressing P_{tac} *pnl*, Tc^{r}	This study
pKNG101-hrp/hrc	pKNG101 carries the in-frame deleted fragment of *hrp/hrc*	This study
pKNG101-dsp/hrp/hrc	pKNG101 carries the in-frame deleted fragment of *dsp/hrp/hrp*	This study
pKNG101-dsp	pKNG101 carries the in-frame deleted fragment of *dsp*	This study
pKNG101-dspE	pKNG101 carries the in-frame deleted fragment of *dspE*	This study
pKNG101-hrpZ	pKNG101 carries the in-frame deleted fragment of *hrpZ*	This study
pKNG101-hrpW	pKNG101 carries the in-frame deleted fragment of *hrpW*	This study
pKNG101-hrpK	pKNG101 carries the in-frame deleted fragment of *hrpK*	This study
pKNG101-hrpN	pKNG101 carries the in-frame deleted fragment of *hrpN*	This study
pKNG101-hrpS	pKNG101 carries the in-frame deleted fragment of *hrpS*	This study
pKNG101-hrpL	pKNG101 carries the in-frame deleted fragment of *hrpL*	This study
pKNG101-hrpX	pKNG101 carries the in-frame deleted fragment of *hrpX*	This study
pKNG101-hrpY	pKNG101 carries the in-frame deleted fragment of *hrpY*	This study
pKNG101-pnl	pKNG101 carries the in-frame deleted fragment of *pnl*	This study
pKNG101-140	pKNG101 carries the in-frame deleted fragment of *C1O30_RS00140 - 00155*	This study
pKNG101-1370	pKNG101 carries the in-frame deleted fragment of *C1O30_RS01370 -01390*	This study
pKNG101-1505	pKNG101 carries the in-frame deleted fragment of *C1O30_RS01505*	This study
pKNG101-2130	pKNG101 carries the in-frame deleted fragment of *C1O30_RS02130*	This study
pKNG101-2370	pKNG101 carries the in-frame deleted fragment of *C1O30_RS02370*	This study
pKNG101-2465	pKNG101 carries the in-frame deleted fragment of *C1O30_RS02465*	This study
pKNG101-2510	pKNG101 carries the in-frame deleted fragment of *C1O30_RS02510*	This study
pKNG101-3460	pKNG101 carries the in-frame deleted fragment of *C1O30_RS03460*	This study

pKNG101-3475	pKNG101 carries the in-frame deleted fragment of *C1O30_RS03475*	This study
pKNG101-4230	pKNG101 carries the in-frame deleted fragment of *C1O30_RS04230*	This study
pKNG101-4475	pKNG101 carries the in-frame deleted fragment of *C1O30_RS04475*	This study
pKNG101-5105	pKNG101 carries the in-frame deleted fragment of *C1O30_RS05105*	This study
pKNG101-6290	pKNG101 carries the in-frame deleted fragment of *C1O30_RS06290*	This study
pKNG101-6880	pKNG101 carries the in-frame deleted fragment of *C1O30_RS06880*	This study
pKNG101-7840	pKNG101 carries the in-frame deleted fragment of *C1O30_RS07840*	This study
pKNG101-8725	pKNG101 carries the in-frame deleted fragment of *C1O30_RS08725*	This study
pKNG101-9650	pKNG101 carries the in-frame deleted fragment of *C1O30_RS09650*	This study
pKNG101-12525	pKNG101 carries the in-frame deleted fragment of *C1O30_RS12525*	This study
pKNG101-13675	pKNG101 carries the in-frame deleted fragment of *C1O30_RS13675*	This study
pKNG101-14540	pKNG101 carries the in-frame deleted fragment of *C1O30_RS14540*	This study
pKNG101-18380	pKNG101 carries the in-frame deleted fragment of *C1O30_RS18380*	This study
pKNG101-18985	pKNG101 carries the in-frame deleted fragment of *C1O30_RS18965 - 18985*	This study
pKNG101-20195	pKNG101 carries the in-frame deleted fragment of *C1O30_RS2019*	This study
pKNG101-20370	pKNG101 carries the in-frame deleted fragment of *C1O30_RS20370*	This study
pKNG101-20725	pKNG101 carries the in-frame deleted fragment of *C1O30_RS20725*	This study

References

Hu M, Li JL, Chen RT, Li WJ, Feng LW, Shi L, Xue Y, Feng XY, Zhang LH, Zhou JN. 2018. *Dickeya zeae* strains isolated from rice, banana and clivia rot plants show great virulence differentials. BMC Microbiol 18:136.

Pritchard LHS, Saddler GS, Parkinson NM, Bertrand V, Elphinstone JG. 2012. Detection of phytopathogens of the genus *Dickeya* using a PCR primer prediction pipeline for draft bacterial genome sequences. Plant Pathol 62:587-596.

Zhang JX, Arif M, Shen HF, Hu J, Sun D, Pu XM, Yang QY, Lin BR. 2020. Genomic divergence between *Dickeya zeae* strain EC2 isolated from rice and previously identified strains, suggests a different rice foot rot strain. PLoS One15:e0240908.

Table S2 The accession numbers of all strains on NCBI

Strains	**Bio Sample**	**Bio Project**	**Assembly**	**GenBank No.**
D.zeae ZJU1202	SAMN02472172	PRJNA159489	GCA_000264075.1	NZ_AJVN00000000.1
D. zeae EC1	SAMN03275446	PRJNA229184	GCA_000816045.1	NZ_CP006929.1
D. zeae DZ2Q	SAMN02469908	PRJNA193475	GCA_000404105.1	NZ_AJVN00000000.1
D. oryzae ZYY5	SAMN11471903	PRJNA533927	GCA_009372235.1	NZ_APMV00000000.1
D. zeae EC2	SAMN09742555	PRJNA483372	GCA_012278405.1	NZ_CP031515.1
D. zeae CSL_RW192	SAMN02470093	PRJNA172883	GCA_000406045.1	NZ_CM001972.1
D. zeae NCPPB_3531	SAMN02470101	PRJNA172950	GCA_000406225.1	NZ_CM001980.1
D. zeae MS1	SAMN01991085	PRJNA194072	GCA_000382585.1	NZ_APWM00000000.1
D. zeae MS2	SAMN08333636	PRJNA429264	GCA_002887555.1	NZ_CP025799.1
D. zeae MK19	SAMN02470105	PRJNA172959	GCA_000406325.1	NZ_CM001985.1
D. zeae NCPPB_3532	SAMN02470083	PRJNA172951	GCA_000400525.1	NZ_CM001858.1
D. zeae NCPPB_2538	SAMN02470089	PRJNA172907	GCA_000406165.1	NZ_CM001977.1
D. zeae Ech586	SAMN02598506	PRJNA33667	GCA_000025065.1	NC_CP001836.1
D. zeae CE1	SAMN09736021	PRJNA483111	GCA_012278555.1	NZ_CP033622.1

Table S3. Primers used in this study

Mutant	Primer	Primer sequence (5'-3')	Amplicon
**Primers of gene deletion**			
Δhrp/hrc	*hrp/hrc-F*	GCGTGTTATCCGCTTTTAGC	*hrp/hrc* mutant detection
	hrp/hrc-R	ATCAGTGAGGTTCCCAGGATAG	
	hrp/hrc-1	ccctgcaggtcgacgATGTTGTTTAGCTGCTGGAG	Upstream fragment of *hrp/hrc* gene cluster
	hrp/hrc-2	CGATGACATGACGCCAGCGAAGCCATGAATATCAGGCAATAAC	
	hrp/hrc-3	GTTATTGCCTGATATTCATGGCTTCGCTGGCGTCATGTCATCG	Downstream fragment of *hrp/hrc* gene cluster
	hrp/hrc-4	ggactatagactataGGATGGGGACGTAATAGCGC	
ΔdspE	*dspE-F*	GTGATGATGTGGATACGATG	*dspE* mutant detection
	dspE-R	GAAATCGGTGCCGCACCAC	
	dspE-1	ccctgcaggtcgacgATCTCTTTAGTACAGACCTC	Upstream fragment of *dspE*
	dspE-2	CAGCACACGGGAGAAAGCCATAAAGCAACGGCATATGTGG	
	dspE-3	CCACATATGCCGTTGCTTTATGGCTTTCTCCCGTGTGCTG	Downstream fragment of *dspE*
	dspE-4	ggactatagactataGCCGAACTGACCTTTCTGGC	
ΔhrpZ	*hrpZ-F*	CGCTCTGCGAACCTTCATC	*hrpZ* mutant detection
	hrpZ-R	GGCGCCTACAGATGGAGAG	
	hrpZ-1	ccctgcaggtcgacgAAGGTTGCTGGCTGACCG	Upstream fragment of *hrpZ*
	hrpZ-2	CCTGATGAAAAGAGGCGAACTCAGGCGCGTGGTTAAACGG	
	hrpZ-3	CCGTTTAACCACGCGCCTGAGTTCGCCTCTTTTCATCAGG	Downstream fragment of *hrpZ*
	hrpZ-4	ggactatagactataGCCGCTATTATTGCCTGCG	
ΔhrpW	*hrpW-F*	TTGCAAAGCAACGGGCTGGA	*hrpW* mutant detection
	hrpW-R	TGGTTTCCACCGTCAGCGGT	
	hrpW-1	ccctgcaggtcgacgCTGGAGGATCTCAGTAAATC	Upstream fragment of *hrpW*
	hrpW-2	ATGGTGCGCTCCCTGTCAGCCAGGTGAGTACGAAATGCGC	
	hrpW-3	GCGCATTTCGTACTCACCTGGCTGACAGGGAGCGCACCAT	Downstream fragment of
	hrpW-4	ggactatagactataGCCGAGATCGGTCTGTACC	

		C	*hrpW*
ΔhrpK	*hrpK-F*	ATGGACCGGAACATCAAGAC	*hrpK* mutant detection
	hrpK-R	CGGATACCTGCAACACGGA	
	hrpK-1	ccctgcaggtcgacgAGAGCAATTGATGCCGCTGT	Upstream fragment of *hrpK*
	hrpK-2	TGGGTATAGACACCGGATAATGCGTCGGCGAAAGTGGAG	
	hrpK-3	CTCCACTTTCGCCGACGCATTATCCGGTGTCTATACCCA	Downstream fragment of *hrpK*
	hrpK-4	ggactatagactataGTAGTTGGAGGATATGGACATC	
ΔhrpN	*hrpN-F*	GCGTGTTATCCGCTTTTAGC	*hrpN* mutant detection
	hrpN-R	GCGCAATAGCAAAACCGATC	
	hrpN-1	ccctgcaggtcgacgATGTTGTTTAGCTGCTGGAG	Upstream fragment of *hrpN*
	hrpN-2	ATGAATGAGGAAACGAAATTTAACGCGCCATGAATATCAG	
	hrpN-3	CTGATATTCATGGCGCGTTAAATTTCGTTTCCTCATTCAT	Downstream fragment of *hrpN*
	hrpN-4	ggactatagactataTGGTGGAGAATGCCATGCGC	
ΔhrpS	*hrpS-F*	CAGGGATATCAACACTACGC	*hrpS* mutant detection
	hrpS-R	TAGACGAGAGTCTGGAAGTG	
	hrpS-1	ccctgcaggtcgacgTCAGTCCTTGAAGCTCATTG	Upstream fragment of *hrpS*
	hrpS-2	ACACTATCGCCCCTCTTCATTTTATCGGGCTGTATCCTGT	
	hrpS-3	ACAGGATACAGCCCGATAAAATGAAGAGGGGCGATAGTGT	Downstream fragment of *hrpS*
	hrpS-4	ggactatagactataCAGGTGCTCTCGCAACTGCG	
ΔhrpL	*hrpL-F*	GATTCACGCTGACTTCTACC	*hrpL* mutant detection
	hrpL-R	CCGTTTTTGAGATGGATGGC	
	hrpL-1	ccctgcaggtcgacgTTTTGTGCTGGGTTTCAAAC	Upstream fragment of *hrpL*
	hrpL-2	GATGGAGAGTGAATGAAATGGCATAAGCTCTAACCCTGCG	
	hrpL-3	CGCAGGGTTAGAGCTTATGCCATTTCATTCACTCTCCATC	Downstream fragment of *hrpL*
	hrpL-4	ggactatagactataGCTGAGGGTCAAAACGTTCC	
ΔhrpX	*hrpX-F*	GCATATCGGCAATCGCATTC	*hrpX* mutant detection
	hrpX-R	TGAAGCTGCCTCAGCGTTAG	

	hrpX-1	ccctgcaggtcgacgAGCATGACCATGATCTGATG	Upstream fragment of *hrpX*
	hrpX-2	GCGCTAGCAAGGAGATGGCACACAGATTTCTCTAAAGGGG	
	hrpX-3	CCCCTTTAGAGAAATCTGTGTGCCATCTCCTTGCTAGCGC	Downstream fragment of *hrpX*
	hrpX-4	ggactatagactataCTGTTATGTGGCTGGATCTG	
ΔhrpY	*hrpY-F*	GAGTGAAGAGAGTGGACCG	*hrpY* mutant detection
	hrpY-R	TTGAGGGCGAACTGGAAGA	
	hrpY-1	ccctgcaggtcgacgACAATGACGTGACTGCTCGA	Upstream fragment of *hrpY*
	hrpY-2	GGGCTACACAGTAGCTTACCGCTAAAGGGGACGAAAACGCGA	
	hrpY-3	TCGCGTTTTCGTCCCCTTTAGCGGTAAGCTACTGTGTAGCCC	Downstream fragment of *hrpY*
	hrpY-4	ggactatagactataCGACGCGCCATCGGGTTAT	
Δdsp	*dsp-F*	CTGGTAGTGTTTTCGCAAATCG	*dsp* and *dsp/hrp/hrc* mutant detection
	dsp-R	GGTATCCATCAACGTATGCAGG	
	dsp-1	ccctgcaggtcgacgAGGGAGAGGTTTCCGGCAC	Upstream fragment of *dsp* gene cluster
	dsp-2	TGAGGGGAGTCGACATTCGAGGTCTGTACTAAAGAGATCT	
	dsp-3	TTTAGTACAGACCTCGAATGTCGACTCCCCTCAGAATATG	Downstream fragment of *dsp* gene cluster
	dsp-4	ggactatagactataGCCACGGCTTCCTTCGCACTGG	
Δ140	140-155-F	AGCCTGATGGTGTTGTTGTCCTC	140-155mutant detection
	140-155-R	CGTACTGGTGGAGAATTTTGC	
	140-155-1	ccctgcaggtcgacgAACCGTGGTGATAGGGAAAAATG	Upstream fragment of *140-155* gene cluster
	140-155-2	CTGCTCCCATTACGCATCGCATTGCCTCCGTGTTTACCGT	
	140-155-3	ACGGTAAACACGGAGGCAATGCGATGCGTAATGGGAGCAG	Downstream fragment of *140-155* gene cluster
	140-155-4	ggactatagactataCGCTGGATCGCATGGGTCTGAC	
Δ1370	1370-1390-F	ACGTAACGATGAAGAACGCGAG	1370-1390 mutant detection
	1370-1390-R	GGCCAAAAAGGTTAACCGCAAG	

	1370-1390-1	ccctgcaggtcgacgATACAACCTGCAGCAAGAGTATG	Upstream fragment of *1370-1390* gene cluster
	1370-1390-2	GGTATCACTGTAGCCTGCGGCAGAGAAAAAACAGCGAACA	
	1370-1390-3	TGTTCGCTGTTTTTTCTCTGCCGCAGGCTACAGTGATACC	Downstream fragment of *1370-1390* gene cluster
	1370-1390-4	ggactatagactataGTTGCAGTATCCAGCGTTGGTC	
Δ1505	1505-F	AGAAAAAACGAGAGTCGGCACG	*1505* mutant detection
	1505-R	TGTCTTTGGGATTACTGACGCC	
	1505-1	ccctgcaggtcgacgCGAAGAAATAGAACAATCCGG	Upstream fragment of 1*505* gene
	1505-2	CGCTGTAATCGCTTCCCGATGCTCGCTTAGCCGAACTCAC	
	1505-3	GTGAGTTCGGCTAAGCGAGCATCGGGAAGCGATTACAGCG	Downstream fragment of 1*505* gene
	1505-4	ggactatagactataACGTCGATACAACCCCAGTGCC	
Δ2130	2130-F	GTGACACAGACAGGGTTGGAAC	*2130* mutant detection
	2130-R	AGACACCAGGTGGTTTTCCTGC	
	2130-1	ccctgcaggtcgacgTCAAGAAGACCGGTCAACCTTG	Upstream fragment of *2130* gene
	2130-2	CATGGATTCATGCTTATACCACCATTCCCTTTTCGCATTC	
	2130-3	GAATGCGAAAAGGGAATGGTGGTATAAGCATGAATCCATG	Downstream fragment of *2130* gene
	2130-4	ggactatagactataGGCGTAAACGCCTCTTTAACCG	
Δ2370	2370-F	CGACAAGGTGGAAAACCTGG	*2370* mutant detection
	2370-R	GACACGATCTCAGCCCACAAG	
	2370-1	ccctgcaggtcgacgCAGGAGTGCATGAAATCAGATC	Upstream fragment of *2370* gene
	2370-2	ACACACCTGCAACTTGAAGTTGAGTCGCCACTGGCTCACA	
	2370-3	TGTGAGCCAGTGGCGACTCAACTTCAAGTTGCAGGTGTGT	Downstream fragment of *2370* gene
	2370-4	ggactatagactataCCCACATTGCTCGCCAGACGG	
Δ2465	2465-F	CAATGTGTCTGGCATTTGCACC	*2465* mutant detection
	2465-R	GATCCAGTTCCGGGTTATCCAG	
	2465-1	ccctgcaggtcgacgTCATGATCCGTCAACCATT	Upstream

		GCAG	fragment of *2465* gene
	2465-2	ACCGATTTAAAGGAGAGAGCGCTACAGCACAATCAGGGAC	
	2465-3	GTCCCTGATTGTGCTGTAGCGCTCTCTCCTTTAAATCGGT	Downstream fragment of *2465*gene
	2465-4	ggactatagactataCCCTTTGACGATACGAACGGC	
Δ2510	2510-F	GCCTGCGAGCAAAAACGGAAAT	*2510* mutant detection
	2510-R	AAGCGGATCCAAATGGTTGGTC	
	2510-1	ccctgcaggtcgacgTGAGCCTTATATTGTTCATCCG	Upstream fragment of *2510* gene
	2510-2	TATTGGCCGTTAACCGTTAGCGCTCCAGCACGCTATGCAA	
	2510-3	TTGCATAGCGTGCTGGAGCGCTAACGGTTAACGGCCAATA	Downstream fragment of *2510* gene
	2510-4	ggactatagactataCTCATCCAGCAGCAGAACCTCCG	
Δ3460	3460-F	CTGATGAGGAGAATCTGATGCG	*3460* mutant detection
	3460-R	TAACTACGATCGATCCCATGCC	
	3460-1	ccctgcaggtcgacgATCTTCTGTTGCGACCATTACAG	Upstream fragment of *3460* gene
	3460-2	ACATCAATTAAAAGAAGAGGACCAAGGCGATTCCATCAGG	
	3460-3	CCTGATGGAATCGCCTTGGTCCTCTTCTTTTAATTGATGT	Downstream fragment of *3460* gene
	3460-4	ggactatagactataCTTCTGACACCCTTCGGCATGG	
Δ3475	3475-F	TCACCACACTGACGCATACGAC	*3475* mutant detection
	3475-R	TTCCAGGCATTGGATTGCCAGG	
	3475-1	ccctgcaggtcgacgATTGAGTATTACTAGTCATCGG	Upstream fragment of *3475* gene
	3475-2	TACGTCGATGACTGGTTACCTCCGAAAAAACGATTGCGGA	
	3475-3	TCCGCAATCGTTTTTTCGGAGGTAACCAGTCATCGACGTA	Downstream fragment of *3475*gene
	3475-4	ggactatagactataCAACGAGGTCAACACCCTGCCC	
Δ4230	4230-F	TTGTTGCGCATCAGCTTTTGGC	*4230* mutant detection
	4230-R	CCGACTGGTACGCAAAATATAG	
	4230-1	ccctgcaggtcgacgCAATTAAGGTATTGGTTTG	Upstream

		CGG	fragment of *4230* gene
	4230-2	TACCGCAATCCTTGCTAGATCGCTGATGCATCTATCATAA	
	4230-3	TTATGATAGATGCATCAGCGATCTAGCAAGGATTGCGGTA	Downstream fragment of *4230* gene
	4230-4	ggactatagactataCACCAGCATGACACCAATCACCG	
Δ4475	4475-F	GATTGGTGGTTGTTGTAATGCG	*4475* mutant detection
	4475-R	GTACATCAATCAGGGCAACGAC	
	4475-1	ccctgcaggtcgacgTTGACTTGTCTGTTTTTATTGC	Upstream fragment of *4475* gene
	4475-2	AAATACCAGAGTACCAGCCTATAGACTCAGCAGGCAGGCC	
	4475-3	GGCCTGCCTGCTGAGTCTATAGGCTGGTACTCTGGTATTT	Downstream fragment of *4475* gene
	4475-4	ggactatagactataGGAACTGGTAGCGGATGTGAGC	
Δ5105	5105-F	GAAAACCCTCAATGGCTCTGCG	*5105* mutant detection
	5105-R	AAATGGCGTCGAGCAGAAAACC	
	5105-1	ccctgcaggtcgacgTTTAGGTAATGCAGTGTGCTGG	Upstream fragment of *5105* gene
	5105-2	ACGCTACTCAGAGGATGTCCCTTTGCAGGTTGTGAATTAG	
	5105-3	CTAATTCACAACCTGCAAAGGGACATCCTCTGAGTAGCGT	Downstream fragment of *5105* gene
	5105-4	ggactatagactataACTGGCTCCTTTGGTCAACGGC	
Δ6290	6290-F	AGACGAGAGAGCAGGGTGTAAC	*6290* mutant detection
	6290-R	GCTAAACACCAACAGGTGGCG	
	6290-1	ccctgcaggtcgacgGCAGTGGTTAAAAAGTTAACC	Upstream fragment of *6290* gene
	6290-2	AGAAATAGCCCTAATGGGACGGTATCTATCCCCTTGATTA	
	6290-3	TAATCAAGGGGATAGATACCGTCCCATTAGGGCTATTTCT	Downstream fragment of *6290* gene
	6290-4	ggactatagactataCTCACGCCATACCAGCTCGTTG	
Δ6880	6880-F	CTCGGTTTCTATTCCCGATTCG	*6290* mutant detection
	6880-R	GGGATCACATTGATAGCTTCGC	
	6880-1	ccctgcaggtcgacgTTCGTTTCTGAGTCAGATGTTG	Upstream fragment of

	6880-2	GAGAAACCAGGAGAGTATCAGTGCATCCATGACACTTTTG	*6880* gene
	6880-3	CAAAAGTGTCATGGATGCACTGATACTCTCCTGGTTTCTC	Downstream fragment of *6880* gene
	6880-4	ggactatagactataTCTGCGTGATGCTGATCACCGC	
Δ7840	7840-F	GGCGAAGATAAGAAAATCGTGG	*7840* mutant detection
	7840-R	GTTGCCAATAATATGCGACCC	
	7840-1	ccctgcaggtcgacgATAAAGATATGTCTTCACGCGT	Upstream fragment of *7840* gene
	7840-2	TGCATTGTCCACCGGTATTTGCGCTGACTAATACCCTCTC	
	7840-3	GAGAGGGTATTAGTCAGCGCAAATACCGGTGGACAATGCA	Downstream fragment of *7840* gene
	7840-4	ggactatagactataGTCAGTGACACCATGCGCACCC	
Δ8725	8725-F	CCAAATACTTTGTCCTGCCAGC	*8725* mutant detection
	8725-R	AACACGATGTCGTGGTGGATTG	
	8725-1	ccctgcaggtcgacgGTAAACCAAATTTTGGAGCTGG	Upstream fragment of *8725* gene
	8725-2	GGCGAACTCTTTTCCTCTGGGTCTCGACACAGATACCCCT	
	8725-3	AGGGGTATCTGTGTCGAGACCCAGAGGAAAAGAGTTCGCC	Downstream fragment of *8725* gene
	8725-4	ggactatagactataCAATGTGTCCATTCGCGAGCGG	
Δ9650	9650-F	CCGATATTGCCATTGTTGCCC	*9650* mutant detection
	9650-R	CAACTCGGCTTGCCTTGATGC	
	9650-1	ccctgcaggtcgacgTTACGATGAGCGGTTATCGCTTG	Upstream fragment of *9650* gene
	9650-2	TCATCAGAAAAGCACACAGGAGTATTAAACAGACACTACA	
	9650-3	TGTAGTGTCTGTTTAATACTCCTGTGTGCTTTTCTGATGA	Downstream fragment of *9650* gene
	9650-4	ggactatagactataGGTTTGCGATCTGTGATCGCAG	
Δ12525	12525-F	AACGCATTGCCTGATCCCATTG	*12525* mutant detection
	12525-R	TACTTCGTCGTACACCAGCTTG	
	12525-1	ccctgcaggtcgacgCTTAATCCATCATGCTATCCAC	Upstream fragment of

	12525-2	GGGAGGCTGGTGGATTATTCGTCAGCGTTTTGCAATCCCT	*12525* gene
	12525-3	AGGGATTGCAAAACGCTGACGAATAATCCACCAGCCTCCC	Downstream fragment of *12525* gene
	12525-4	ggactatagactataGAAGTGAATCTGGCTCAGTGCG	
Δ13675	13675-F	CATACACGGTCTTAAGCGAGTG	*13675* mutant detection
	13675-R	CCATTAGTACCAGTAATGCAGG	
	13675-1	ccctgcaggtcgacgCCTTTTCTGCAGTAAACATGCC	Upstream fragment of *13675* gene
	13675-2	TCGACGGATCAGGGCTGATGCGACATGAATTCCTTTCTCC	
	13675-3	GGAGAAAGGAATTCATGTCGCATCAGCCCTGATCCGTCGA	Downstream fragment of *13675* gene
	13675-4	ggactatagactataACAGGTCAGAGGTAGTGCCGAC	
Δ14540	14540-F	ACGGTCATTGTGTATGCAGACC	*14540* mutant detection
	14540-R	TCACGCCATTGCCCAGTTTAAG	
	14540-1	ccctgcaggtcgacgATTGTTAATGGTTGCTAACACC	Upstream fragment of *14540* gene
	14540-2	AGGCTGTTTCGACTGCCAGGTAATCACGACCCTAATATTG	
	14540-3	CAATATTAGGGTCGTGATTACCTGGCAGTCGAAACAGCCT	Downstream fragment of *14540* gene
	14540-4	ggactatagactataCGGTTTGCTGATTGGTATCGCC	
Δ18380	18380-F	AATCAAGGCGTCATAGGGTGCAC	*18380* mutant detection
	18380-R	CCTCATCCCAGCCAAACAATTC	
	18380-1	ccctgcaggtcgacgCGACAATCAAATCCACATCATC	Upstream fragment of *18380* gene
	18380-2	ATGACTCTGGGAGGAACGGTGTTGTTGTCGTTTTCGGTAT	
	18380-3	ATACCGAAAACGACAACAACACCGTTCCTCCCAGAGTCAT	Downstream fragment of *18380* gene
	18380-4	ggactatagactataCAGTAACTCCGGTGCGTGCAAC	
Δ18985	18965-18985-F	GGAAAACGCCTATCGCTATAGC	18965-*18985* mutant detection
	18965-18985-R	CACGCAACGTTATAGTAGCTGG	
	18965-18985	ccctgcaggtcgacgACACAGCACGATTCGCATC	Upstream

	-1	AAAC	fragment of 18965-*18985* gene
	18965-18985-2	GTTATCCTGTACTTCCGACCTGTCGTAAACCCTTCACTTT	
	18965-18985-3	AAAGTGAAGGGTTTACGACAGGTCGGAAGTACAGGATAAC	Downstream fragment of 18965-*18985* gene
	18965-18985-4	ggactatagactataGGCATGGTGCTCCGAATGTTGG	
Δ20195	20195-F	ACCACCAGCACAATATTTACCG	*20195* mutant detection
	20195-R	CCGCTGCAAACCAAATTGGTGA	
	20195-1	ccctgcaggtcgacgACCACCAGCACAATATTTACCG	Upstream fragment of *20195* gene
	20195-2	ACGATATCAACGGACCAGGTATGATTTCCCGATGTCTATC	
	20195-3	GATAGACATCGGGAAATCATACCTGGTCCGTTGATATCGT	Downstream fragment of *20195* gene
	20195-4	ggactatagactataGCGACGTGGGGTAATCAACTCGC	
Δ20370	20370-F	GAACATCATCAGCCACATGAGC	*20370* mutant detection
	20370-R	GGACTTCGAATGACTTCTCCAC	
	20370-1	ccctgcaggtcgacgTGACGTCACACTAACCTTGCAAC	Upstream fragment of *20370* gene
	20370-2	TGCCGTGGTCATCGTGACGCCTGCATCGTGTGCCTTATTC	
	20370-3	GAATAAGGCACACGATGCAGGCGTCACGATGACCACGGCA	Downstream fragment of *20370* gene
	20370-4	ggactatagactataCGTTCTGCCACAGGTACAGCCG	
Δ20725	20725-F	GCGACAACGCCTCAAAATAGAG	*20725* mutant detection
	20725-R	TCATTGTAGAAGAGCCTGACGC	
	20725-1	ccctgcaggtcgacgGAGATAATCCTCAAAGCTCAAC	Upstream fragment of *20725* gene
	20725-2	GTGTAAGCGTTATCCTGAGCGATAAGAACTCCCCTGTGTG	
	20725-3	CACACAGGGGAGTTCTTATCGCTCAGGATAACGCTTACAC	Downstream fragment of *20725* gene
	20725-4	ggactatagactataCATACCTTGCGCAAGGTTCCTG	
Reference gene	infB-F	AATATCGAAGCCAATCACGC	*infB* detection
	infB-R	GATTTCATAAAGCACCAGCG	
**RT-PCR primers**	pelN-F	CCTATACCGATGGCGCGAAT	*pelN* detection
	pelN-R	TATCTGTTGCCAGACGCAGG	

**for detection of CWDEs genes**	pelL-F	CTGGTGGCGTGACGGTAATA	*pelL* detection
	pelL-R	TACCGTAGCCAGCGAGGTAT	
	pelI-F	GGCAACTGCACCATCGAAAAC	*pelI* detection
	pelI-R	CAGTCACCGCAGGAGCGCC	
	pelA-F	CGGCAGCGTGTTATCGGAAG	*pelA* detection
	pelA-R	TGCGGCAGTCATTGGTTGA	
	pelE -F	GCAGCCTGCTGTCTGAATCC	*pelE* detection
	pelE -R	AAGCGCTGAAACCAAAACCG	
	pelD -F	CTGTACAGCTTCGGCCTGG	*pelD* detection
	pelD -R	GTACGCCGTCAGACCACAA	
	pelC -F	GGCACCTGGGTGCTGAAAAA	*pelC* detection
	pelC -R	TTTTACCCACACCGGCGTAAT	
	pelB -F	GTTGCAACGCGGTGGTAATG	*pelB* detection
	pelB -R	CGGCGTTAACGTAAGCCTTG	
	pelZ -F	TGGATGAGCGACAATCTGGC	*pelZ* detection
	pelZ -R	GGTGGGATAACCACCGACTT	
	pelW -F	CCACGGTTTATTTGTGCGCT	*pelW* detection
	pelW -R	TCTGGGTAAATAAACTCCGTGTCA	
	pelX -F	TATTTCACTGCGCACTCACC	*pelX* detection
	pelX -R	TCAGGAAATCACCCAACTGC	
	paeX -F	GTACATGCGATTGATGAC	*paeX* detection
	paeX -R	TATCTGGTTGATTCCCGGTC	
	paeY -F	TGATACCGGTGTTGTTGACCC	*peaY* detection
	paeY -R	TCGCGGTAATAAGGGTAGCG	
	pemA -F	GCCATTGGCCAGACGGTATT	*pemA* detection
	pemA -R	TTGCTTTGGGTGTATTCCGCT	
	pehN-F	TATCTGGTTGATTCCCGGTC	*pehN* detection
	pehN-F	TGTCGGTGGCGCGGTAAGC	
	pehK -F	AAGGTCTGTACGACGTTCGC	*pehK* detection
	pehK -R	CATGTTGTGCCATGTCAGCC	
	pehX -F	GTACCACCGATTACACCCCC	*pehX* detection
	pehX -R	GAAGATCCGCTACGCAGGTT	
	rhiE1-F	GTTGTTGTACGAAGAACT	*rhiE1* detection
	rhiE1-R	GATAATCGCATCCTGATG	
	rhiE2-F	GCTTATCGCAATCAGTATA	*rhiE2* detection
	rhiE2-R	CGCCTGAATAATAATCCA	
	xynA-F	GTCTGGTGGTATATCCGTCG	xynA detection
	xynA-R	GTTACTGATATTCAGCGACAG	
	celZ -F	TATCAGCAATGCGAACTGGG	*celZ* detection
	celZ -R	CAGCCCAATCTTTACTGACCC	
	celY -F	TGGCCATCGCGTTTCAGTTA	*celY* detection
	celY -R	GGTCACGTACCGCCAGTAAA	
	bglA -F	GGTATGGGAAGATGGCGTCG	*bglA* detection

	bglA -R	TCACCCGCTGATACCAGTAGA	
	bgaX-F	CCATCATGAAGGGTCGCTGG	*bgaX* detection
	bgaX-R	GCGGTTTTGCGCTGTCATAA	
	bglB -F	GGTCTGGGGGCGAAAGATAA	*bglB* detection
	bglB -R	GACATTTCCGCTTTTGAGGC	
	nagZ -F	TTTTCTCGGATGACCTGTCG	*nagZ* detection
	nagZ -R	CCGGTACTTTGACCACCGA	
	celH –F	CTAAAAGCACGCGAAGACGG	*celH* detection
	celH -R	ATCGTCCAGTTCGAACGCC	
	ifaA -F	GCGATGGCTGGTACGACTTT	*ifaA* detection
	ifaA -R	CTTCAAACAACAGGCCCTGAC	
	prtX -F	TGGCGGTTCAGGCAATGATC	*petX* detection
	prtX -R	GATTGCTGACATTGGATGACG	
	prtC -F	CAGGCAACGACGTGTTGTAC	*prtC* detection
	prtC -R	CCAGAGAACTGATCCTGCACAA	
	prtB -F	CATTTTCGTCTACGGCAGCG	*prtB* detection
	prtB -R	TGATGCTATCAGCCGCATCC	
	prtG -F	AATGACGTGCTGTACGGTGA	*prtG* detection
	prtG -R	TCTGGTTCGCTTGACCATCC	
	prtF -F	AATGACGTGCTGTACGGTGA	*prtF* detection
	prtF -R	TTGCCGGTGGTGAACGTATT	
	prtE -F	CGCGTTTAGCCAGAGTACGA	*prtE* detection
	prtE -R	TGATAAACGAACGCTCCCCG	
	prtD-F	CCAGCCTTGATAGTGACGGT	*prtD* detection
	prtD-R	CTGATTGGCAGCGCTACGTT	
**RT-PCR primers for detection of regulation genes**	fis-F	CTCATACAGGTCATTCAC	*fis* detection
	fis-R	TTTCCACTGTAAACTCTC	
	slyA-F	GGGAGAGATTAGCAATAAC	*slyA* detection
	slyA-R	AAAAGGTGGAGTCTATCG	
	vfmE-F	TTACCAAGACGTACAATATAG	*vfmE* detection
	vfmE-R	TTTGCCGAAAATGATGTC	
	pecS-F	TTACCAAAGACCTTATCC	*pecS* detection
	pecS-R	AAGTCTCAGATATTGTTCA	
	kdgR-F	AATCAAGTCCACATTCTG	*kdgR* detection
	kdgR-R	TCACTGAACTATCACAAC	
	pecT-F	GATTACGGTTCGTTCAGT	*pecT* detection
	pecT-R	GTTCGTCAGCTCATTGTC	
Reference gene	atpD-F	TACCACGAAATGACCGACTCC	qRT-PCR analysis
	atpD-R	CATTTCTTCTGCCAGTGTCG	
**qPCR primers**	00560-F	TTGTTGTTACCCTGGTTTG	qRT-PCR analysis
	00560-R	TGATACTCATAGAGCAAGACA	
	02835-F	ACGGTAATGACGATATTC	qRT-PCR analysis
	02835-R	TTGCTAATGGTGATGTAA	

	00675-F	AAGAATCTAATGCCCAACA	qRT-PCR analysis
	00675-R	GGTAGTGATCCAGAATCG	
	00680-F	GTATCTTCCAGCACCTATC	qRT-PCR analysis
	00680-R	GGATCAGTGTCATCAGTT	
	00895-F	GCCTACTTTAACTACCTGAA	qRT-PCR analysis
	00895-R	TTGTTGTTGCTGTTGATC	
	01895-F	GGAGAATGGTCACAAGAA	qRT-PCR analysis
	01895-R	TTATGGGTGTCGTTATGC	
	02135-F	GGAGTAATGCCGTAACAT	qRT-PCR analysis
	02135-R	AGTGACAACAAGGGAAAG	
	02140-F	TCTGACAACCATTATCTGTT	qRT-PCR analysis
	02140-R	TTGCCGACATATCCTTAC	
	03200-F	CGAACTTATCCGTCACAT	qRT-PCR analysis
	03200-R	CATGCCGATATAGCTCTTA	
	03400-F	ACAATGATAGACGAGCAAT	qRT-PCR analysis
	03400-R	GCAGGACCTCATCAATAG	
	04855-F	TGCTGCCGATTATTCTTA	qRT-PCR analysis
	04855-R	CGTTAATGATGATGGAGTAAT	
	05230-F	ATTACATCGTCTTCCTATCC	qRT-PCR analysis
	05230-R	ATCCTGAAACTGATTATTTAGC	
	08240-F	ATTCGGCGTCGTTATCTA	qRT-PCR analysis
	08240-R	GCATCAATCAGTCCATCC	
	08785-F	CGCCATATTCAGTGTGAT	qRT-PCR analysis
	08785-R	TTAGCCAGTTCGGTATTC	
	11855-F	CTTATGTGCGAGATTATGATT	qRT-PCR analysis
	11855-R	TGTATTCCTGTGAGTTGAC	
	12170-F	ATAACATCACCATTACCGTA	qRT-PCR analysis
	12170-R	GTCATTGAGTTGTAGGAATAG	
	12625-F	CAGATTGGCGATTGTAAC	qRT-PCR analysis
	12625-R	CCGATGTTCAGTACGATT	
	13325-F	TGCTCTTATTAACGCTACT	qRT-PCR analysis
	13325-R	CTCAGGTTGTCAATGGTA	
	13375-F	CAATGCCAACTACTACCA	qRT-PCR analysis
	13375-R	TTCCTGATCGCTAATGAC	
	13415-F	CGAATACGGCTTCTATCA	qRT-PCR analysis
	13415-R	TGCGATCAGTACCAATAC	
	14125-R	CAGGATTTCACCCGTAAT	qRT-PCR analysis
	14125-R	GTAGCGTCTTGTATCTGTT	
	14425-F	CAAACAGGCTACCGTATT	qRT-PCR analysis
	14425-R	ATTGATGGCGAGTGAATG	
	15345-F	CGCTAATACCACCGTTAT	qRT-PCR analysis
	15345-R	CATCACCTGCTTCATAGT	
	15350-F	AACAACTTATCTATGCCTTCT	qRT-PCR

	15350-R	GATACCATCAGCGTAGTG	analysis
	16815-F	ATCAGCAGATTAACCAGTT	qRT-PCR analysis
	16815-R	CGCCTGAATCATCTTTTG	
	18405-F	GTTGATTATCTACGATGACAG	qRT-PCR analysis
	18405-R	CAGGCAATGAACGGTAAT	
	19255-F	GTCATCGTCCATGTCTTC	qRT-PCR analysis
	19255-R	TGCTGGTTGTTATTCTGT	
	19500-F	ATGACTAGCATTTCTCAACT	qRT-PCR analysis
	19500-R	TGATATTATATCCGCCTGTT	
	19635-F	GATACCGATACCGATGAAG	qRT-PCR analysis
	19635-R	GAAATCCGACAGGAAATTG	
	20330-F	CCAGTTGTTATCCGACTC	qRT-PCR analysis
	20330-R	CTCCATCACACGATAAGG	
	20375-F	ATAAAGAAGCCCAGAAACA	qRT-PCR analysis
	20375-R	ACATCTATCGAGACATTACC	
	20935-F	TCCTCTGGTCAAGGTAAA	qRT-PCR analysis
	20935-R	TTTCTCTTTCGGCATAGTT	
	00105-F	ATAAGCAGTTAGTGGCATTG	qRT-PCR analysis
	00105-R	ACTTGTCATCCAGGTAATCA	
	02185-F	TTGTTGTTACCCTGGTTTG	qRT-PCR analysis
	02185-R	GAGGTTGAAGAAGATGAGATG	
	02465-F	GGATATAGCCAACCTCAGT	qRT-PCR analysis
	02465-R	ATCTTCCTTGCTCAGTGT	
	02835-F	ACGGTAATGACGATATTC	qRT-PCR analysis
	02835-R	TTGCTAATGGTGATGTAA	
	04030-F	ACGGTCATAGGTGATACG	qRT-PCR analysis
	04030-R	AACCAGACTCCACTATCG	
	04130-F	GTCATTTTCGCCTCATTC	qRT-PCR analysis
	04130-R	ACTGTAGTTGTTTCTGGAA	
	08565-F	GTATTGCTGTATTCTCCTTGAT	qRT-PCR analysis
	08565-R	ATATTGTTCTGTCTCATTCACTT	
	08570-F	GCGATATGTGCGATAAGC	qRT-PCR analysis
	08570-R	TCTGATTCTCATTAGCGGTTA	
	08580-F	AGCAAGGTTGGATTCTCA	qRT-PCR analysis
	08580-R	ATGGCTGTTAATGGTATTGTT	
	15015-F	CCGTAGTGTTGACAATATCTC	qRT-PCR analysis
	15015-R	TGTTATGCTGATGACAATAGAAT	
	qdspE-F	AGAAATGCAGTCGCTGACCA	qRT-PCR analysis
	qdspE-R	GGCTATCCTGGTTCTGACCG	
	qhrpZ-F	CAGAAAGGTCAGTTCGGCCA	qRT-PCR analysis
	qhrpZ-R	AGCTCGCTGGCGTTATTACT	
	qhrpW-F	CGGTAAACCGGCTAACGTCT	qRT-PCR analysis
	qhrpW-R	ACGTTCAGCCCTTCACTGTC	

	qhrpK-F	TTACCAGCGGCATCCATTGT	qRT-PCR analysis
	qhrpK-R	AAACAAAAGGGCGTGCTGTC	
	qhrpN-F	GCGAAAGCGCTGAGTAAACC	qRT-PCR analysis
	qhrpN-R	TATCTTGTCACCGACGACGC	
	qhrpJ-F	GCCTTGTCTCAGGAAGTGGAA	qRT-PCR analysis
	qhrpJ-R	GCCACTGTAATTGCGTCATGG	
	qhrpP-F	TGCAAACGACACCCGTCAT	qRT-PCR analysis
	qhrpP-R	AACGTGCTTGCCAGCGAAC	
	qhrpB-F	CGCCGACAACAAAGCCTG	qRT-PCR analysis
	qhrpB-R	GCAGGTTGGTCAGTTTTTCCA	
	qhrpA-F	ACTGTCTAACGCTGCTGCTA	qRT-PCR analysis
	qhrpA-R	CAGAACTGGATGGCTTTGGC	
	qhrpS-F	CATCAACTTTCCGGTTGCCC	qRT-PCR analysis
	qhrpS-R	GTTTGGGTGGCGACAATCAC	
	qhrpY-F	CGCGCTATATTGACCACAGC	qRT-PCR analysis
	qhrpY-R	TCAGACGATTAAACGCTGGCT	
	qhrpX-F	GAGTGGCTGCGTGATGATTTT	qRT-PCR analysis
	qhrpX-R	AATCATGCCGTCGCGATTTT	
	qhrpL-F	GCACTTGCCGGAGGACA	qRT-PCR analysis
	qhrpL-R	GGAAAGACGCGAACGAACAG	
**Protein expressio n primers**	pET32a-Bam HI-hrpL-F	gccatggctgatatcggatccCGATGGAGAGTGAAT GAA	*hrpL* protein expression
	pET32a-Hin dIII-hrpL-R	ctcgagtgcggccgcaagcttTTATGCATCAACGGC CTGG	
	pET32a-F	TAATACGACTCACTATAGGG	Protein detection
	pET32a-R	GCTAGTTATTGCTCAGCGG	
**EMSA probe primers**	pSsp-F	ATGATGGCAAGGTATGCCCC	*Ssp* probe
	pSsp-R	ATGACTCATCCTGGCAATGA	
	phrpA-F	TTTATCGGGCTGTATCCTGT	*hrpA* probe
	phrpA-R	GATAGATATCTCCAGTTAACAT	
	nmphrpA-F	TCCATGAACACGATGACT	*hrpA* probe without *hrp-box*
	nmphrpA-R	CTGAGCCTTAGAGGTCAT	
	phrpN-F	AATTTCGTTTCCTCATTCA	*hrpN* probe
	phrpN-R	TTTTACGTTAAACCAGCA	
	phrpX-F	GCTCTAACCCTGCGCGGTAC	*hrpX* probe
	phrpX-R	GGTACATCGTCCGGCTGCTG	
	pdspE-F	GTTTTGTATATCCGACAGCG	*dspE* probe
	pdspE-R	AGTTCGCCTCTTTTCATCAG	
	phrpK-F	GCAGGACGGCGCGCAGGCGT	*hrpK* probe
	phrpK-R	ACAGGCCGATACGATTATCA	
	phrpZ-F	GGTACATCGTCCGGCTGCTG	*hrpZ* probe
	phrpZ-R	GCGTGATGTTGATATCAGCCAT	
	p12170-F	GACGTGATGGGACTGGAACT	*12170* probe

	p12170-R	AGTCTTGTCCACTTCGTTGT	
	nmp12170-F	GCCTCACGGTTGACGACCTG	*12170* probe without *hrp-box*
	nmp12170-R	AGTCTTGTCCACTTCGTTGT	
	p16815-F	AGGGACAGTCCCCTGCTAAG	*16815* probe
	p16815-R	CCTGCATCACAACGATCTGG	
	p19500-F	GGACAACACGCTCGCGTGTT	*19500* probe
	p19500-R	ATATCCGCCTGTTTCTTCAG	

Table S4. Specific genes in JZL7

Gene ID	Function
JZL7_000390, JZL7_002519,JZL7_002989	hypothetical protein
JZL7_000113, JZL7_001961	nucleotidyl transferase AbiEii/AbiGii toxin family protein
JZL7_000114, JZL7_001960	hypothetical protein
JZL7_000863, JZL7_002866	hypothetical protein
JZL7_001663, JZL7_001670	helix-turn-helix transcriptional regulator
JZL7_001664, JZL7_001671	toprim domain-containing protein
JZL7_001677, JZL7_002834	hypothetical protein
JZL7_002013, JZL7_002026	alpha/beta hydrolase
JZL7_001578, JZL7_002786	glycoside hydrolase family 19 protein
JZL7_002873, JZL7_004219	ISNCY/IS481 family transposase
JZL7_000121	hypothetical protein
JZL7_000124	AlpA family phage regulatory protein
JZL7_000219	hypothetical protein
JZL7_000233	elongation factor tu
JZL7_000424	hypothetical protein
JZL7_000425	hypothetical protein
JZL7_000432	hypothetical protein
JZL7_000434	DUF1778 domain-containing protein
JZL7_000436	hypothetical protein
JZL7_000439	hypothetical protein
JZL7_000747	hypothetical protein
JZL7_000855	hypothetical protein
JZL7_000857	hypothetical protein
JZL7_000858	DUF3644 domain-containing protein
JZL7_000859	hypothetical protein
JZL7_001091	hypothetical protein
JZL7_001281	restriction endonuclease
JZL7_001318	hypothetical protein
JZL7_001319	hypothetical protein
JZL7_001408	nuclear transport factor 2 family protein
JZL7_001410	acyl carrier protein
JZL7_001457	AAA family ATPase
JZL7_001458	AAA family ATPase
JZL7_001459	hypothetical protein
JZL7_001491	hypothetical protein
JZL7_001543	hypothetical protein
JZL7_001561	hypothetical protein
JZL7_001565	AAA family ATPase
JZL7_001566	hypothetical protein

JZL7_001567	hypothetical protein
JZL7_001568	DUF3164 family protein
JZL7_001569	hypothetical protein
JZL7_001570	DUF2786 domain-containing protein
JZL7_001571	hypothetical protein
JZL7_001572	hypothetical protein
JZL7_001574	hypothetical protein
JZL7_001576	hypothetical protein
JZL7_001579	DUF2644 domain-containing protein
JZL7_001580	hypothetical protein
JZL7_001581	hypothetical protein
JZL7_001582	hypothetical protein
JZL7_001583	DUF1804 family protein
JZL7_001584	hypothetical protein
JZL7_001585	phage terminase large subunit
JZL7_001589	hypothetical protein
JZL7_001590	hypothetical protein
JZL7_001591	head protein
JZL7_001592	hypothetical protein
JZL7_001593	DUF1320 domain-containing protein
JZL7_001595	hypothetical protein
JZL7_001596	phage tail protein
JZL7_001597	phage tail protein
JZL7_001598	hypothetical protein
JZL7_001599	hypothetical protein
JZL7_001601	multidrug DMT transporter permease
JZL7_001602	phage tail protein
JZL7_001603	phage baseplate assembly protein
JZL7_001604	hypothetical protein
JZL7_001605	baseplate J/gp47 family protein
JZL7_001606	DUF2313 domain-containing protein
JZL7_001609	protein mom
JZL7_001635	hypothetical protein
JZL7_001636	hypothetical protein
JZL7_001643	hypothetical protein
JZL7_001649	hypothetical protein
JZL7_001650	hypothetical protein
JZL7_001967	hypothetical protein
JZL7_001968	DUF2326 domain-containing protein
JZL7_001969	hypothetical protein
JZL7_001971	type II toxin-antitoxin system RelB/DinJ family antitoxin
JZL7_001973	ATPase
JZL7_001974	hypothetical protein

JZL7_001976	chromosome partitioning protein ParB
JZL7_001977	hypothetical protein
JZL7_001981	DUF2958 domain-containing protein
JZL7_001982	hypothetical protein
JZL7_001983	DUF2285 domain-containing protein
JZL7_001984	helix-turn-helix domain-containing protein
JZL7_001985	replication initiator protein A
JZL7_001986	hypothetical protein
JZL7_001988	chromosome partitioning protein ParB
JZL7_001989	DUF2840 domain-containing protein
JZL7_001990	S26 family signal peptidase
JZL7_001991	hypothetical protein
JZL7_001992	relaxase/mobilization nuclease and DUF3363 domain-containing protein
JZL7_001998	EexN family lipoprotein
JZL7_001999	conjugal transfer protein TraG
JZL7_002000	ribbon-helix-helix protein%2C CopG family
JZL7_002002	conjugal transfer protein TrbC
JZL7_002003	conjugal transfer protein TrbD
JZL7_002006	P-type conjugative transfer protein TrbL
JZL7_002007	conjugal transfer protein TrbF
JZL7_002008	P-type conjugative transfer protein TrbG
JZL7_002009	TrbI/VirB10 family protein
JZL7_002011	hypothetical protein
JZL7_002019	TfoX/Sxy family protein
JZL7_002025	hypothetical protein
JZL7_002118	hypothetical protein
JZL7_002161	hypothetical protein
JZL7_002190	NAD-dependent epimerase/dehydratase family protein
JZL7_002344	hypothetical protein
JZL7_002375	hypothetical protein
JZL7_002682	hypothetical protein
JZL7_002754	hypothetical protein
JZL7_002756	DUF4376 domain-containing protein
JZL7_002758	DUF2612 domain-containing protein
JZL7_002759	phage baseplate protein
JZL7_002760	hypothetical protein
JZL7_002762	hypothetical protein
JZL7_002763	hypothetical protein
JZL7_002764	hypothetical protein
JZL7_002767	ORF6N domain-containing protein
JZL7_002768	hypothetical protein
JZL7_002769	hypothetical protein

JZL7_002770	hypothetical protein
JZL7_002771	DUF3383 domain-containing protein
JZL7_002772	hypothetical protein
JZL7_002773	hypothetical protein
JZL7_002774	hypothetical protein
JZL7_002775	DUF4054 domain-containing protein
JZL7_002776	hypothetical protein
JZL7_002777	DUF2184 domain-containing protein
JZL7_002778	hypothetical protein
JZL7_002779	DUF2213 domain-containing protein
JZL7_002781	DUF1073 domain-containing protein
JZL7_002782	phage terminase large subunit
JZL7_002783	terminase small subunit
JZL7_002784	hypothetical protein
JZL7_002785	lysis protein
JZL7_002787	phage holin family protein
JZL7_002788	hypothetical protein
JZL7_002789	DUF1456 family protein
JZL7_002790	hypothetical protein
JZL7_002791	antiterminator
JZL7_002792	YlcG family protein
JZL7_002793	RusA family crossover junction endodeoxyribonuclease
JZL7_002794	DUF1364 family protein
JZL7_002796	DUF1367 family protein
JZL7_002798	hypothetical protein
JZL7_002800	ASCH domain-containing protein
JZL7_002801	hypothetical protein
JZL7_002802	hypothetical protein
JZL7_002803	hypothetical protein
JZL7_002806	GntR family transcriptional regulator
JZL7_002807	hypothetical protein
JZL7_002808	tRNA-(guanine-N1)-methyltransferase
JZL7_002809	hypothetical protein
JZL7_002810	helix-turn-helix domain-containing protein
JZL7_002811	hypothetical protein
JZL7_002812	hypothetical protein
JZL7_002813	siphovirus Gp157 family protein
JZL7_002814	hypothetical protein
JZL7_002815	DUF4224 domain-containing protein
JZL7_002816	tyrosine-type recombinase/integrase
JZL7_002850	hypothetical protein
JZL7_002851	hypothetical protein
JZL7_002865	hypothetical protein

JZL7_002869	hypothetical protein
JZL7_002871	hypothetical protein
JZL7_002872	hypothetical protein
JZL7_002874	hypothetical protein
JZL7_002882	hypothetical protein
JZL7_002883	IS110 family transposase
JZL7_002884	hypothetical protein
JZL7_002885	hypothetical protein
JZL7_002886	hypothetical protein
JZL7_002975	hypothetical protein
JZL7_003055	hypothetical protein
JZL7_003057	transposase
JZL7_003058	nucleotide-binding protein
JZL7_003059	hypothetical protein
JZL7_003161	hypothetical protein
JZL7_003210	hypothetical protein
JZL7_003213	DUF4238 domain-containing protein
JZL7_003416	hypothetical protein
JZL7_003593	hypothetical protein
JZL7_003759	hypothetical protein
JZL7_003919	hypothetical protein
JZL7_004020	hypothetical protein
JZL7_004063	hypothetical protein
JZL7_004226	SMI1/KNR4 family protein
JZL7_004235	hypothetical protein
JZL7_004317	hypothetical protein
JZL7_000265	hypothetical protein
JZL7_001278	hypothetical protein
JZL7_001412	3-deoxy-7-phosphoheptulonate synthase
JZL7_001418	transposase
JZL7_001575	regulatory protein GemA
JZL7_001594	DUF1834 family protein
JZL7_001674	hypothetical protein
JZL7_001908	his operon leader peptide
JZL7_001965	restriction endonuclease
JZL7_002511	SMP-30/gluconolactonase/LRE family protein
JZL7_002761	hypothetical protein
JZL7_002766	hypothetical protein
JZL7_002780	phage head morphogenesis protein
JZL7_002795	phosphoadenosine phosphosulfate reductase family protein
JZL7_002870	hypothetical protein
JZL7_004209	SMI1/KNR4 family protein

Table S5. Genes encoding for the Type IV secreation systems in genomes of Ech586, EC1, MS2, EC2, CE1 and JZL7

System	Gene name	Gene ID in every sample						Gene function
		Ech586	EC1	MS2	EC2	CE1	JZL7	
T4SS-1	*traR*	/					JZL7_001997	WP_058982533.1; putative transcriptional regulator, LysR family [*Stenotrophomonas maltophilia*]
	RSc2587	/					JZL7_001998	WP_024302289.1; hypothetical protein [*Pseudogulbenkiania* sp. MAI-1]
	trbG	/					JZL7_001999	WP_024302290.1; conjugal transfer protein TraG [*Pseudogulbenkiania* sp. MAI-1]
	RSc2585	/					JZL7_002000	WP_047748482.1; MULTISPECIES: ribbon-helix-helix protein, CopG family [Enterobacteriaceae]
	trbB	/					JZL7_002001	WP_020831651.1; P-type conjugative transfer ATPase TrbB [Ralstonia solanacearum]
	trbC	/					JZL7_002002	WP_024302293.1; conjugal transfer protein TrbC [*Pseudogulbenkiania* sp. MAI-1]
	trbD	/					JZL7_002003	WP_024110176.1; MULTISPECIES: conjugal transfer protein TrbD [*Proteobacteria*]
	trbE	/					JZL7_002004	WP_020831654.1; Conjugative transfer protein TrbE [*Ralstonia solanacearum* FQY_4]
	trbJ	/					JZL7_002005	WP_070155718.1; P-type conjugative transfer protein TrbJ [*Sphingobium phenoxybenzoativorans*]
	trbL	/					JZL7_002006	WP_024302297.1; P-type conjugative transfer protein TrbL [*Pseudogulbenkiania* sp. MAI-1]
	trbF	/					JZL7_002007	WP_070158053.1; conjugal transfer protein TrbF [*Sphingobium phenoxybenzoativorans*]
	trbG	/					JZL7_002008	WP_020831658.1; P-type conjugative transfer protein TrbG [*Ralstonia solanacearum*]

	trbI	/			DWV07_03760 (incomplete, 25% coverage)	DWG24_11540	JZL7_002009	WP_024302300.1; conjugal transfer protein TrbI [*Pseudogulbenkiania* sp. MAI-1]
	RSc2574	/	/	/	/	DWG24_11535	JZL7_002010	CUV57490.1; Conjugal transfer protein [Ralstonia solanacearum]
T4SS-2	*virB1*	Dd586_1461	W909_12990	C1O30_RS07565	/	/	JZL7_002854	WP_029456300.1; transglycosylase [*Dickeya zeae*]
	virB2	Dd586_1460	W909_12995	C1O30_RS07560	/	/	JZL7_002855	WP_012769221.1; MULTISPECIES: conjugal transfer protein [*Dickeya solani*]
	virB4	Dd586_1459	W909_13000	/	/	/	JZL7_002856	WP_012769220.1; CagE TrbE VirB component of type IV transporter system [*Dickeya chrysanthemi* Ech1591]
	virB5	Dd586_1458	W909_13005	/	/	/	JZL7_002857	WP_024106434.1; TriD protein [Dickeya dianthicola]
	virB6	Dd586_1456	W909_13015	/	/	/	JZL7_002859	WP_038925980.1; conjugal transfer protein TrbL [*Dickeya zeae*]
	virB8	Dd586_1455	W909_13025	/	/	/	JZL7_002860	WP_042860390.1; type IV secretion system protein [*Dickeya* sp. NCPPB 3274]
	virB9	Dd586_1454	W909_13030	/	/	/	JZL7_002861	WP_012884156.1; P-type conjugative transfer protein VirB9 [*Dickeya zeae*]
	virB10	Dd586_1453	W909_13035	/	/	/	JZL7_002862	WP_038925983.1; membrane protein [Dickeya zeae]
	virB11	Dd586_1452	W909_13040	/	/	/	JZL7_002863	WP_029729589.1; P-type DNA transfer ATPase VirB11 [*Dickeya dianthicola*]

Table S6 The annotation results of the 10 areas in the JZL7 genome

Area ID	Gene ID	Gene similarity				Gene function
		JZL7	MS2	Ech586	EC1	
**Area A**	JZL7_000113	1	0	0	0	WP_017050811.1; nucleotidyl transferase AbiEii/AbiGii toxin family protein [*Vibrio ordalii*]
	JZL7_000114	1	0	0	0	WP_038924272.1; hypothetical protein [*Dickeya dadantii*]
	JZL7_000115	1	0	0	0.96	WP_016943502.1; hypothetical protein [*Dickeya zeae* EC1, W909_00515]
	JZL7_000116	1	0	0	0.96	WP_038902959.1; hypothetical protein [*Dickeya zeae*]
	JZL7_000117	1	0	0	0.97	WP_016943500.1; hypothetical protein [*Dickeya zeae*]
	JZL7_000118	1	0	0	0.909010989	WP_016943499.1; hypothetical protein [*Dickeya zeae*]
	JZL7_000119	1	0	0	0	WP_015848306.1; abortive infection protein [*Dickeya chrysanthemi*]
	JZL7_000120	1	0.142786885	0.273770492	0	OOB84387.1; transposase [*Leclercia adecarboxylata*]
	JZL7_000120	1	0	0	0.214285714	AHA68739.1; Transposase [*Shigella dysenteriae* 1617]
	JZL7_000122	1	0	0	0	WP_012770265.1; transposase IS3/IS911 family protein [*Dickeya chrysanthemi* Ech1591]
	JZL7_000122	1	0.156462585	0.213979592	0	WP_012770264.1; Integrase catalytic region [*Dickeya chrysanthemi*]
	JZL7_000123	1	0	0.56	0	WP_063158240.1; Uncharacterised protein [*Enterobacter cloacae*]
	JZL7_000124	1	0	0	0	WP_032742728.1; MULTISPECIES: AlpA family phage regulatory protein [*Klebsiella pneumoniae*]
	JZL7_000125	1	0	0	0	KMH87827.1; Uncharacterised protein [*Klebsiella pneumoniae*]
	JZL7_000126	1	0.260334129	0.315823389	0.265966587	WP_015848293.1; integrase family protein [*Dickeya chrysanthemi*]
**Area B**	JZL7_001560	1	0	0.548264463	0	WP_038900891.1; alpha/beta hydrolase [*Dickeya dadantii*]
	JZL7_001561	1	0	0	0	WP_060435877.1; Uncharacterised protein [*Serratia marcescens*]
	JZL7_001562	1	0.267318841	0.267318841	0.267318841	OWF83056.1; hypothetical protein [*Yersinia frederiksenii*, B4903_03450]
	JZL7_001563	1	0.492142857	0.499714286	0.509142857	WP_023311928.1; MULTISPECIES: transcriptional regulator [Enterobacteriaceae]
	JZL7_001564	1	0	0	0	WP_026595081.1; transposase [*Dickeya dianthicola*]
	JZL7_001565	1	0	0	0	WP_067486496.1; XRE family transcriptional regulator [*Dickeya* sp. CSL RW240]
	JZL7_001566	1	0	0	0	WP_024108006.1; hypothetical protein [*Dickeya dianthicola*]
	JZL7_001567	1	0	0	0	WP_024110372.1; MULTISPECIES: hypothetical protein [*Dickeya*]
	JZL7_001568	1	0	0	0	WP_024108004.1; DUF3164 domain-containing protein [*Dickeya dianthicola*]
	JZL7_001569	1	0	0	0	WP_038914730.1; hypothetical protein [*Dickeya zeae*]

	JZL7_001570	1	0	0	0	WP_067487254.1; DUF2786 domain-containing protein [*Dickeya* sp. CSL RW240]
	JZL7_001571	1	0	0	0	WP_024110368.1; hypothetical protein [*Dickeya dianthicola*]
	JZL7_001572	1	0	0	0	WP_050570627.1; hypothetical protein [*Dickeya zeae*]
	JZL7_001573	1	0	0	0.718983051	WP_024107998.1; hypothetical protein [*Dickeya dianthicola*]
	JZL7_001574	1	0	0	0	WP_029729409.1; hypothetical protein [*Dickeya dianthicola*]
	JZL7_001575	1	0	0	0	WP_067487516.1; regulatory protein GemA [*Dickeya* sp. CSL RW240]
	JZL7_001576	1	0	0	0	WP_024110363.1; MULTISPECIES: hypothetical protein [*Dickeya*]
	JZL7_001577	1	0.196571429	0.196571429	0.191657143	WP_024110362.1; hypothetical protein [*Dickeya dianthicola*]
	JZL7_001578	1	0.417830189	0.417830189	0.396933962	WP_038914736.1; endolysin [*Dickeya zeae*]
	JZL7_001579	1	0	0	0	WP_038914144.1; DUF2644 domain-containing protein [*Dickeya zeae*]
	JZL7_001580	1	0	0	0	WP_050570965.1; hypothetical protein [*Dickeya zeae*]
	JZL7_001581	1	0	0	0	WP_024107989.1; MULTISPECIES: hypothetical protein [*Dickeya*]
	JZL7_001582	1	0	0	0	WP_024107988.1; hypothetical protein [*Dickeya dianthicola*]
	JZL7_001583	1	0	0	0	WP_067486422.1; DUF1804 domain-containing protein [*Dickeya* sp. CSL RW240]
	JZL7_001584	1	0	0	0	WP_024107985.1; MULTISPECIES: hypothetical protein [*Dickeya*]
	JZL7_001585	1	0	0	0	NA
	JZL7_001585	1	0	0	0	WP_035346448.1; phage protein, partial [*Dickeya* sp. DW 0440]
	JZL7_001586	1	0	0	0.177428571	WP_024107983.1; DUF935 domain-containing protein [*Dickeya dianthicola*]
	JZL7_001587	1	0	0	0.211015801	WP_038906872.1; phage head morphogenesis protein [*Dickeya zeae*]
	JZL7_001588	1	0	0	0.271823204	WP_038906873.1; phage virion morphogenesis protein [*Dickeya zeae*]
	JZL7_001589	1	0	0	0	WP_035345435.1; hypothetical protein [*Dickeya* sp. DW 0440]
	JZL7_001590	1	0	0	0	WP_039493617.1; hypothetical protein [*Pectobacterium carotovorum*]
	JZL7_001591	1	0	0	0	WP_009112852.1; Mu-like major head subunit gpT, prophage protein [*Brenneria* sp. EniD312]
	JZL7_001592	1	0	0	0	WP_029729402.1; hypothetical protein [*Dickeya dianthicola*]
	JZL7_001593	1	0	0	0.285944056	WP_024107976.1; DUF1320 domain-containing protein [*Dickeya dianthicola*]
	JZL7_001594	1	0	0	0	WP_038909451.1; DUF1834 domain-containing protein [*Dickeya zeae*]
	JZL7_001595	1	0	0	0	WP_038914148.1; hypothetical protein [*Dickeya zeae*]
	JZL7_001596	1	0	0	0	WP_038914150.1; tail sheath protein [*Dickeya zeae*]
	JZL7_001597	1	0	0	0	WP_024107972.1; MULTISPECIES: hypothetical protein [*Dickeya*]
	JZL7_001598	1	0	0	0	WP_038914151.1; hypothetical protein [*Dickeya zeae*]
	JZL7_001599	1	0	0	0	NA
	JZL7_001600	1	0	0	0.103179973	WP_038914152.1; phage tail length tape measure protein [*Dickeya zeae*]

	JZL7_001601	1	0	0	0	WP_038906887.1; multidrug DMT transporter permease [*Dickeya zeae*]
	JZL7_001602	1	0	0	0	WP_038906888.1; tail protein [*Dickeya zeae*]
	JZL7_001603	1	0	0	0	WP_038906889.1; phage baseplate assembly protein V [*Dickeya zeae*]
	JZL7_001604	1	0	0	0	WP_024107965.1; hypothetical protein [*Dickeya dianthicola*]
	JZL7_001605	1	0	0	0.173711048	WP_038906892.1; tail protein [*Dickeya zeae*]
	JZL7_001606	1	0	0	0	WP_038914158.1; DUF2313 domain-containing protein [*Dickeya zeae*]
	JZL7_001607	1	0.232909091	0.243818182	0.276831169	WP_015848323.1; Tail Collar domain-containing protein [*Dickeya chrysanthemi*]
	JZL7_001608	1	0.520772947	0.487922705	0.673333333	WP_038907879.1; tail fiber assembly protein [*Dickeya zeae*]
	JZL7_001609	1	0	0	0	WP_038909453.1; hypothetical protein [*Dickeya zeae*]
**Area C**	JZL7_001661	1	0	0	0	WP_039297187.1; hypothetical protein [*Pectobacterium atrosepticum*]
	JZL7_001662	1	0.347837838	0.360540541	0	WP_033569057.1; type I toxin-antitoxin system SymE family toxin [*Dickeya* sp. 2B12]
	JZL7_001663	1	0.220902256	0	0	WP_033569058.1; XRE family transcriptional regulator [*Dickeya* sp. 2B12]
	JZL7_001664	1	0.09308642	0.099444444	0.099444444	WP_011095751.1; DnaG primase-like protein [*Pectobacterium atrosepticum*]
	JZL7_001665	1	0.294157303	0.294157303	0.294157303	WP_033569060.1; recombinase XerD [*Dickeya* sp. 2B12]
	/	1	0	0	0	NA
	JZL7_001667	1	0	0	0	AIU90251.1; hypothetical protein [*Pectobacterium carotovorum* subsp. *odoriferum*, BCS7_20965]
	JZL7_001668	1	0	0	0	WP_039297187.1; hypothetical protein [*Pectobacterium atrosepticum*]
	JZL7_001669	1	0.347837838	0.360540541	0	WP_033569057.1; type I toxin-antitoxin system SymE family toxin [*Dickeya* sp. 2B12]
	JZL7_001670	1	0.220902256	0	0	WP_033569058.1; XRE family transcriptional regulator [*Dickeya* sp. 2B12]
	JZL7_001671	1	0.09308642	0.099444444	0.099444444	WP_011095751.1; DnaG primase-like protein [*Pectobacterium atrosepticum*]
	JZL7_001672	1	0.27875	0.27875	0.27875	WP_033569060.1; recombinase XerD [*Dickeya* sp. 2B12]
	/	1	0	0	0	NA
	JZL7_001674	1	0	0	0	
	JZL7_001675	1	0.124615385	0	0	WP_023638980.1; SMI1/KNR4 family protein [*Dickeya zeae*]
**Area D**	JZL7_001960	1	0	0	0	WP_024106831.1; hypothetical protein [*Dickeya dianthicola*]
	JZL7_001961	1	0	0	0	WP_024106830.1; nucleotidyl transferase AbiEii/AbiGii toxin family protein [*Dickeya dianthicola*]
	JZL7_001962	1	0.153134328	0	0.208432836	ABF08351.1; Phage-related protein (modular protein) [*Cupriavidus metallidurans* CH34]
	JZL7_001963	1	0	0	0	WP_027483871.1; DUF2971 domain-containing protein [*Rhodanobacter* sp. OR87]

	JZL7_001964	1	0	0	0	WP_005922487.1; hypothetical protein [*Xanthomonas axonopodis*|*X. citri*]
	JZL7_001965	1	0	0	0	EGH05167.1; restriction endonuclease-like protein [*Pseudomonas amygdali* pv. *aesculi* str. 0893_23]
	JZL7_001967	1	0	0	0	BAQ38027.1; hypothetical protein [*Pseudomonas aeruginosa*, PA257_1409]
	JZL7_001968	1	0	0	0	WP_087824425.1; DUF2326 domain-containing protein [*Klebsiella quasipneumoniae*]
	JZL7_001969	1	0	0	0	WP_042852620.1; hypothetical protein [*Pseudomonas aeruginosa*]
	JZL7_001970	1	0	0	0	WP_087824427.1; hypothetical protein [*Klebsiella quasipneumoniae*]
	JZL7_001971	1	0	0	0	WP_087824428.1; type II toxin-antitoxin system antitoxin, RelB/DinJ family [*Klebsiella quasipneumoniae*]
	JZL7_001972	1	0.459642857	0.394345238	0.394345238	WP_020831622.1; DNA repair protein RadC [*Ralstonia solanacearum*]
	JZL7_001973	1	0	0	0	WP_066695051.1; ATPase [Comamonadaceae bacterium CCH4-C5]
	JZL7_001974	1	0	0	0	WP_033944595.1; MULTISPECIES: hypothetical protein [*Pseudomonas aeruginosa*|*Pseudomonas* sp.]
	JZL7_001975	1	0	0	0	WP_029729674.1; hypothetical protein [*Dickeya dianthicola*]
	JZL7_001975	1	0.518436364	0.528218182	0.518436364	WP_004355968.1; DUF945 domain-containing protein [*Thauera phenylacetica*]
	JZL7_001976	1	0	0	0	WP_075258233.1; chromosome partitioning protein ParB [*Herbaspirillum* sp. WT00C]
	JZL7_001978	1	0.07729927	0	0	WP_075259884.1; DNA helicase [*Herbaspirillum* sp. WT00C]
	JZL7_001979	1	0	0.651452991	0.651452991	WP_020831628.1; DUF736 domain-containing protein [*Ralstonia solanacearum*]
	JZL7_001980	1	0	0	0.2875	WP_020831629.1; XRE family transcriptional regulator [*Ralstonia solanacearum*]
	JZL7_001981	1	0	0	0	WP_024110199.1; DUF2958 domain-containing protein [*Dickeya dianthicola*]
	JZL7_001983	1	0	0	0	WP_024110198.1; DUF2285 domain-containing protein [*Dickeya dianthicola*]
	JZL7_001984	1	0	0	0	WP_020831632.1; hypothetical protein [*Ralstonia solanacearum*]
	JZL7_001985	1	0	0	0	WP_083276845.1; RepA replication protein [*Sphingobium phenoxybenzoativorans*]
	JZL7_001986	1	0	0	0	NA
	JZL7_001987	1	0	0	0	WP_020831634.1; Chromosome (plasmid) partitioning protein ParA [*Ralstonia solanacearum*]
	JZL7_001988	1	0	0	0	WP_020831635.1; hypothetical protein [*Ralstonia solanacearum*]
	JZL7_001989	1	0	0	0	WP_070155751.1; DUF2840 domain-containing protein [*Sphingobium phenoxybenzoativorans*]
	JZL7_001990	1	0	0	0	WP_070155749.1; peptidase [*Sphingobium phenoxybenzoativorans*]
	JZL7_001991	1	0	0	0	WP_081282095.1; hypothetical protein [*Ralstonia solanacearum*]

	JZL7_001992	1	0	0	0	WP_020831638.1; Type IV secretory pathway, VirD2 components (relaxase) [*Ralstonia solanacearum*]
	JZL7_001993	1	0.344615385	0.336	0.249230769	WP_082604188.1; transcriptional regulator [*Acidovorax* sp. Root217]
	JZL7_001994	1	0	0	0	WP_071076923.1; hypothetical protein [*Janthinobacterium lividum*]
	JZL7_001995	1	0.314529915	0.314529915	0.304700855	WP_061953114.1; NAD(P)-dependent oxidoreductase [*Cupriavidus pauculus*]
	JZL7_001996	1	0	0	0	WP_018607136.1; hypothetical protein [*Uliginosibacterium gangwonense*]
	JZL7_001997	1	0.265286624	0.265286624	0.265286624	WP_058982533.1; putative transcriptional regulator, LysR family [*Stenotrophomonas maltophilia*]
	JZL7_001998	1	0	0	0	WP_024302289.1; hypothetical protein [*Pseudogulbenkiania* sp. MAI-1]
	JZL7_001999	1	0	0	0	WP_024302290.1; conjugal transfer protein TraG [*Pseudogulbenkiania* sp. MAI-1]
	JZL7_002000	1	0	0	0	WP_047748482.1; MULTISPECIES: ribbon-helix-helix protein, CopG family [Enterobacteriaceae]
	JZL7_002001	1	0.106666667	0.198248588	0.176129944	WP_020831651.1; P-type conjugative transfer ATPase TrbB [*Ralstonia solanacearum*]
	JZL7_002002	1	0	0	0	WP_024302293.1; conjugal transfer protein TrbC [*Pseudogulbenkiania* sp. MAI-1]
	JZL7_002003	1	0	0	0	WP_024110176.1; MULTISPECIES: conjugal transfer protein TrbD [*Proteobacteria*]
	JZL7_002004	1	0	0.156151961	0.18629902	WP_020831654.1; Conjugative transfer protein TrbE [*Ralstonia solanacearum* FQY_4]
	JZL7_002005	1	0	0	0.197479339	WP_070155718.1; P-type conjugative transfer protein TrbJ [*Sphingobium phenoxybenzoativorans*]
	JZL7_002006	1	0	0	0	WP_024302297.1; P-type conjugative transfer protein TrbL [*Pseudogulbenkiania* sp. MAI-1]
	JZL7_002007	1	0	0	0	WP_070158053.1; conjugal transfer protein TrbF [*Sphingobium phenoxybenzoativorans*]
	JZL7_002008	1	0	0	0	WP_020831658.1; P-type conjugative transfer protein TrbG [*Ralstonia solanacearum*]
	JZL7_002009	1	0	0.125	0.134511628	WP_024302300.1; conjugal transfer protein TrbI [*Pseudogulbenkiania* sp. MAI-1]
	JZL7_002010	1	0	0	0	CUV57490.1; Conjugal transfer protein [*Ralstonia solanacearum*]
	JZL7_002011	1	0	0	0	WP_019939521.1; hypothetical protein [*Bordetella* sp. FB-8]
**Area E**	JZL7_002753	1	0	0	0.5425	WP_038669502.1; MULTISPECIES: hypothetical protein [*Dickeya*|*Dickeya chrysanthemi*|*Dickeya solani*]
	JZL7_002754	1	0	0	0	WP_033576172.1; hypothetical protein [*Dickeya chrysanthemi*]

	/	1	0	0	0	NA
	JZL7_002755	1	0.062645503	0.074391534	0.069497354	WP_050516096.1; hypothetical protein [*Dickeya chrysanthemi*]
	JZL7_002756	1	0	0	0	WP_033576171.1; DUF4376 domain-containing protein [*Dickeya chrysanthemi*]
	JZL7_002757	1	0.369736842	0.279356725	0.304005848	WP_050516095.1; hypothetical protein [*Dickeya chrysanthemi*]
	JZL7_002758	1	0	0	0	WP_033576170.1; DUF2612 domain-containing protein [*Dickeya chrysanthemi*]
	JZL7_002759	1	0	0	0	WP_033576169.1; phage baseplate protein [*Dickeya chrysanthemi*]
	JZL7_002760	1	0	0	0	WP_033576168.1; hypothetical protein [*Dickeya chrysanthemi*]
	JZL7_002761	1	0	0	0	WP_033576167.1; hypothetical protein [*Dickeya chrysanthemi*]
	JZL7_002762	1	0	0	0	WP_033576166.1; hypothetical protein [*Dickeya chrysanthemi*]
	JZL7_002763	1	0	0	0	WP_033576165.1; hypothetical protein [*Dickeya chrysanthemi*]
	JZL7_002764	1	0	0	0	WP_033576164.1; hypothetical protein [*Dickeya chrysanthemi*]
	JZL7_002765	1	0.085152057	0.778604651	0.090017889	WP_033576163.1; lysozyme [*Dickeya chrysanthemi*]
	JZL7_002766	1	0	0	0	YP_007006965.1; hypothetical protein [*Pectobacterium* phage ZF40, ZF40_0056]
	JZL7_002767	1	0	0	0	WP_023640358.1; hypothetical protein [*Dickeya zeae*]
	JZL7_002768	1	0	0	0	WP_082170758.1; hypothetical protein [*Yersinia pekkanenii*]
	JZL7_002769	1	0	0	0	WP_033576161.1; hypothetical protein [*Dickeya chrysanthemi*]
	JZL7_002770	1	0	0	0	WP_033576160.1; hypothetical protein [*Dickeya chrysanthemi*]
	JZL7_002771	1	0	0	0	WP_033576159.1; DUF3383 domain-containing protein [*Dickeya chrysanthemi*]
	JZL7_002772	1	0	0	0	WP_033576158.1; hypothetical protein [*Dickeya chrysanthemi*]
	JZL7_002773	1	0	0	0	WP_033576157.1; hypothetical protein [*Dickeya chrysanthemi*]
	JZL7_002774	1	0	0	0	WP_009113084.1; hypothetical protein [*Brenneria* sp.]
	JZL7_002775	1	0	0	0	WP_050516094.1; DUF4054 domain-containing protein [*Dickeya chrysanthemi*]
	JZL7_002776	1	0	0	0	WP_033576155.1; hypothetical protein [*Dickeya chrysanthemi*]
	JZL7_002777	1	0	0	0	WP_033576154.1; DUF2184 domain-containing protein [*Dickeya chrysanthemi*]
	JZL7_002778	1	0	0	0	WP_033576153.1; hypothetical protein [*Dickeya chrysanthemi*]
	JZL7_002779	1	0	0	0	WP_033576152.1; DUF2213 domain-containing protein [*Dickeya chrysanthemi*]
	JZL7_002780	1	0	0	0	WP_033576217.1; phage head morphogenesis protein [*Dickeya chrysanthemi*]
	JZL7_002781	1	0	0	0	WP_033576151.1; phage-associated protein [*Dickeya chrysanthemi*]
	JZL7_002782	1	0	0	0	WP_033576150.1; terminase [*Dickeya chrysanthemi*]
	JZL7_002783	1	0	0	0	*AIU88322.1; terminase small subunit [Pectobacterium carotovorum* subsp. *odoriferum*]
	JZL7_002784	1	0	0	0	WP_019705274.1; MULTISPECIES: hypothetical protein [*Klebsiella*|*Klebsiella pneumoniae*|*Klebsiella quasipneumoniae*]

	JZL7_002785	1	0	0	0	WP_005967173.1; lysis protein [*Pectobacterium wasabiae*]
	JZL7_002786	1	0.376923077	0.376923077	0.356971154	WP_033576147.1; endolysin [*Dickeya chrysanthemi*]
	JZL7_002787	1	0	0	0	WP_033576146.1; phage holin family protein [*Dickeya chrysanthemi*]
	JZL7_002788	1	0	0	0	WP_038667760.1; MULTISPECIES: membrane protein [*Dickeya*|*Dickeya chrysanthemi*|*Dickeya solani*]
	JZL7_002789	1	0	0	0	WP_080638422.1; DUF1456 domain-containing protein [*Dickeya zeae*]
	JZL7_002790	1	0	0	0	WP_023640387.1; hypothetical protein [*Dickeya zeae*]
	JZL7_002791	1	0	0	0	WP_023640388.1; late gene antiterminator protein [*Dickeya zeae*]
	JZL7_002793	1	0	0	0	WP_023640389.1; RusA family crossover junction endodeoxyribonuclease [*Dickeya zeae*]
	JZL7_002794	1	0	0	0	WP_038910922.1; hypothetical protein [*Dickeya dadantii*]
	JZL7_002795	1	0	0	0	WP_023640391.1; phosphoadenosine phosphosulfate reductase [*Dickeya zeae*]
	JZL7_002796	1	0	0	0	WP_023640392.1; DUF1367 domain-containing protein [*Dickeya zeae*]
	JZL7_002797	1	0	0.349206349	0	WP_072033796.1; hypothetical protein [*Dickeya chrysanthemi*]
	JZL7_002798	1	0	0	0	WP_033576131.1; hypothetical protein [*Dickeya chrysanthemi*]
	JZL7_002799	1	0	0	0	NA
	JZL7_002800	1	0	0	0	WP_023640395.1; ASCH domain-containing protein [*Dickeya zeae*]
	JZL7_002801	1	0	0	0	SAF75342.1; Uncharacterised protein [*Enterobacter cloacae*]
	JZL7_002802	1	0	0	0	WP_038667736.1; MULTISPECIES: hypothetical protein [*Dickeya*|*Dickeya chrysanthemi*|*Dickeya solani*]
	JZL7_002803	1	0	0	0	WP_038910918.1; hypothetical protein [*Dickeya dadantii*]
	JZL7_002803	1	0	0	0	NA
	JZL7_002804	1	0.332921109	0.332921109	0.341918977	WP_038667730.1; MULTISPECIES: helicase DnaB [*Dickeya*|*Dickeya chrysanthemi*|*Dickeya solani*]
	JZL7_002805	1	0	0	0.211940299	WP_050570237.1; hypothetical protein [*Dickeya dadantii*]
	JZL7_002806	1	0	0	0	WP_081943124.1; MULTISPECIES: helix-turn-helix domain-containing protein [*Dickeya*]
	JZL7_002806	1	0	0	0	NP_755075 pathogenicity island hypothetical protein c3193 [*Escherichia coli* CFT073] - unknown
	JZL7_002807	1	0	0	0	WP_033576127.1; hypothetical protein [*Dickeya chrysanthemi*]
	JZL7_002808	1	0	0	0	WP_033576126.1; tRNA-(guanine-N1)-methyltransferase [*Dickeya chrysanthemi]*
	JZL7_002809	1	0	0	0	WP_033576211.1; MULTISPECIES: hypothetical protein [*Dickeya*]
	JZL7_002810	1	0	0	0	WP_033576125.1; transcriptional regulator [*Dickeya chrysanthemi*]

	JZL7_002811	1	0	0	0	WP_038910912.1; hypothetical protein [*Dickeya dadantii*]
	JZL7_002812	1	0	0	0	WP_038667707.1; MULTISPECIES: hypothetical protein [*Dickeya*|*Dickeya chrysanthemi*|*Dickeya solani*]
	JZL7_002813	1	0	0	0	WP_033576123.1; siphovirus Gp157 family protein [*Dickeya chrysanthemi*]
	JZL7_002815	1	0	0	0	WP_071842535.1; MULTISPECIES: DUF4224 domain-containing protein [*Dickeya*]
	JZL7_002816	1	0	0	0.122546125	WP_038667701.1; MULTISPECIES: integrase [*Dickeya*|*Dickeya chrysanthemi*|*Dickeya solani*]
**Area F**	JZL7_003049	1	0	0	0	WP_057083755.1; hypothetical protein [*Dickeya solani*]
	JZL7_003050	1	0	0	0	CQH41474.1; Mu P family protein [*Yersinia enterocolitica*]
	JZL7_003051	1	0	0	0.97	NA
	JZL7_003052	1	0	0.693740458	0.85	WP_033576632.1; hypothetical protein [*Dickeya chrysanthemi]*
	JZL7_003053	1	0	0	0	WP_039532449.1; nucleotidyl transferase AbiEii/AbiGii toxin family protein [*Pectobacterium carotovorum*]
	/	1	0	0	0	NA
	/	1	0	0	0	
	JZL7_003054	1	0.385517241	0.75	0.395402299	WP_033071089.1; integrase [*Pectobacterium parmentieri*]
	JZL7_003054	1	0.40625	0.5625	0	NA
	JZL7_003055	1	0	0	0	WP_085539450.1; hypothetical protein [*Burkholderia pseudomallei*]
	JZL7_003056	1	0	0	0.225	OAT75038.1; transposase [*Mangrovibacter phragmitis*]
	JZL7_003056	1	0.142786885	0.273770492	0	OOB84387.1; transposase [*Leclercia adecarboxylata*]
	JZL7_003058	1	0	0	0	WP_083563544.1; hypothetical protein [*Oceanococcus atlanticus*]
	JZL7_003059	1	0	0	0	ESA94891.1; hypothetical protein [*Klebsiella pneumoniae* 909957, HMPREF1619_06102]
	JZL7_003060	1	0.272213115	0.258934426	0.272213115	WP_061333370.1; putative iron-sulfur binding protein [*Marinobacter excellens* LAMA 842]
	JZL7_003061	1	0	0	0	WP_083484274.1; hypothetical protein [*Loktanella* sp. 3ANDIMAR09]
	JZL7_003063	1	0.173104925	0.173104925	0.173104925	SDU36698.1; diguanylate cyclase with PAS/PAC and GAF sensors [*Pseudomonas salegens*]
	JZL7_003065	1	0	0	0	NA
**Area G**	/	1	0	0	0	
	JZL7_003864	1	0	0	0	WP_039999444.1; iron-molybdenum cofactor biosynthesis protein NifQ [*Dickeya chrysanthemi*]

	JZL7_003865	1	0	0	0	WP_040003311.1; nitrogenase cofactor biosynthesis protein NifB [*Dickeya chrysanthemi*]
	JZL7_003866	1	0.288183556	0.288183556	0.288183556	WP_039999442.1; nif-specific transcriptional activator NifA [*Dickeya chrysanthemi*]
	JZL7_003867	1	0.07913215	0.076804734	0.068994083	WP_039999440.1; nitrogen fixation negative regulator NifL [*Dickeya chrysanthemi*]
	JZL7_003868	1	0.407670455	0.417613636	0.397727273	WP_022635015.1; flavodoxin [*Dickeya solani*]
	JZL7_003869	1	0.095703704	0.095703704	0.095703704	WP_050583247.1; nitrogen fixation protein NifM [*Dickeya chrysanthemi*]
	JZL7_003870	1	0	0	0	WP_039999427.1; nitrogen fixation protein NifZ [*Dickeya chrysanthemi*]
	JZL7_003871	1	0	0	0	WP_050583246.1; nitrogen fixation protein NifW [*Dickeya chrysanthemi*]
	JZL7_003872	1	0.296396867	0.296396867	0.296396867	WP_039999425.1; homocitrate synthase [*Dickeya chrysanthemi*]
	JZL7_003873	1	0.388615023	0.388615023	0.397652582	WP_050583245.1; cysteine desulfurase NifS [*Dickeya chrysanthemi*]
	JZL7_003874	1	0.2068	0.2068	0.2068	WP_059110782.1; Fe-S cluster assembly protein NifU [*Dickeya solani*]
	JZL7_003875	1	0	0	0	WP_039999416.1; nitrogen fixation protein NifX [*Dickeya chrysanthemi*]
	JZL7_003876	1	0	0	0	WP_039999413.1; nitrogenase iron-molybdenum cofactor biosynthesis protein NifN [*Dickeya chrysanthemi*]
	JZL7_003877	1	0	0	0	WP_039999412.1; nitrogenase iron-molybdenum cofactor biosynthesis protein NifE [*Dickeya chrysanthemi*]
	JZL7_003878	1	0	0	0	WP_039999411.1; protein NifY [*Dickeya chrysanthemi*]
	JZL7_003879	1	0	0	0	WP_039999409.1; protein NifT [*Dickeya chrysanthemi*]
	JZL7_003880	1	0	0	0	WP_039999407.1; nitrogenase molybdenum-iron protein subunit beta [*Dickeya chrysanthemi*]
	JZL7_003881	1	0	0	0	WP_038923999.1; nitrogenase molybdenum-iron protein alpha chain [*Dickeya dadantii*]
	JZL7_003882	1	0	0	0	WP_039999404.1; nitrogenase iron protein [*Dickeya chrysanthemi*]
**Area H**	JZL7_001272	1	0.140231284	0.890444309	0.882927572	WP_080650183.1; hypothetical protein [*Dickeya zeae*]
	JZL7_001273	1	0.89	0	0	WP_038926039.1; hypothetical protein [*Dickeya zeae*]
	JZL7_001274	1	0.902155172	0	0.864827586	WP_033570960.1; type I addiction module toxin, SymE family [*Dickeya* sp. 2B12]
	JZL7_001275	1	0.92	0.299449541	0.413944954	WP_038913510.1; type I toxin-antitoxin system SymE family toxin [*Dickeya zeae*]
	JZL7_001276	1	0.892622951	0	0	WP_019846697.1; MULTISPECIES: XRE family transcriptional regulator [*Dickeya*]
	JZL7_001277	1	0.345268817	0.851096774	0.849010753	WP_038926038.1; type IV secretion protein Rhs [*Dickeya zeae*]
	JZL7_001278	1	0	0	0	WP_081639065.1; hypothetical protein [*Dickeya chrysanthemi*]
	JZL7_001279	1	0.94	0	0	WP_023639307.1; hypothetical protein [*Dickeya zeae*]

	JZL7_001280	1	0.943448276	0	0.96	WP_080646396.1; type I addiction module toxin, SymE family [*Dickeya zeae*]
	JZL7_001281	1	0	0	0	WP_038903846.1; restriction endonuclease [*Dickeya zeae*]
	JZL7_001282	1	0.570494418	0.793891547	0.779330144	P24211; Putative protein RhsE *Escherichia coli* (strain K12) rhsE
	JZL7_001283	1	0.933974359	0	0	WP_033569739.1; hypothetical protein [*Dickeya* sp. 2B12]
	JZL7_001284	1	0	0.96	0	WP_012884003.1; hypothetical protein [*Dickeya zeae*]
	JZL7_001285	1	0.912215909	0.808465909	0	WP_038913515.1; cell wall assembly protein [*Dickeya zeae*]
	JZL7_001286	1	0.95	0	0.88	WP_080646396.1; type I addiction module toxin, SymE family [*Dickeya zeae*]
	JZL7_001287	1	0.848325359	0	0	WP_038926039.1; hypothetical protein [*Dickeya zeae*]
	JZL7_001288	1	0.93	0	0	WP_038915847.1; SMI1/KNR4 family protein [*Dickeya zeae*]
	JZL7_001289	1	0.93	0.94	0.522580645	WP_027712634.1; hypothetical protein [*Dickeya chrysanthemi*]
	JZL7_001290	1	0.98	0.98	0.97	WP_019846559.1; amidohydrolase [*Dickeya zeae*]
	/	1	0	0	0	NA
	/	1	0	0	0	
**Area I**	/	1	0	0	0	
	JZL7_001405	1	0.98	0.677957958	0.327027027	WP_038913604.1; NADPH: quinone oxidoreductase [*Dickeya zeae*]
	JZL7_001407	1	0.195655738	0.195655738	0.195655738	WP_039302885.1; MarR family transcriptional regulator [*Pectobacterium betavasculorum*]
	JZL7_001408	1	0	0	0	WP_038193414.1; nuclear transport factor 2 family protein [*Xenorhabdus bovienii*]
	JZL7_001409	1	0.170812365	0.21372394	0.19666427	WP_049100446.1; thioester reductase [*Klebsiella oxytoca*]
	JZL7_001409	1	0	0	0	NA
	JZL7_001410	1	0.227272727	0.315844156	0.315844156	WP_047723767.1; iron aquisition yersiniabactin synthesis enzyme (Irp2) [*Klebsiella oxytoca*]
	JZL7_001411	1	0.302924901	0.302924901	0.306719368	WP_038193409.1; Amino acid adenylation domain-containing protein [*Xenorhabdus bovienii* str. *kraussei* Becker Underwood]
	JZL7_001412	1	0	0	0	WP_074023628.1; 3-deoxy-D-arabinose-heptulosonic-7-phosphate synthase [*Xenorhabdus eapokensis*]
	JZL7_001413	1	0.410229885	0.410229885	0.410229885	WP_038193407.1; 2, 3-dihydro-2, 3-dihydroxybenzoate dehydrogenase [*Xenorhabdus bovienii*]
	JZL7_001414	1	0.485849057	0.485849057	0.485849057	WP_038193404.1; 2, 3-dihydro-2, 3-dihydroxybenzoate synthetase [*Xenorhabdus bovienii*]
	JZL7_001415	1	0.127771084	0.127771084	0.127771084	WP_038193401.1; conserved hypothetical protein [*Xenorhabdus bovienii* str. *kraussei* Becker Underwood]
	JZL7_001416	1	0.285939553	0.285939553	0.285939553	WP_039322017.1; daunorubicin resistance protein DrrC [*Pectobacterium*

						betavasculorum]
	JZL7_001417	1	0.261349398	0.261349398	0.261349398	CDH03349.1; Drug resistance efflux protein [*Xenorhabdus bovienii* str. *feltiae* Moldova]
	JZL7_001418	1	0	0	0	NA
	JZL7_001420	1	0.091046931	0.96	0.98	WP_038903933.1; cyclic peptide transporter [*Dickeya zeae*]
	JZL7_001421	1	0.067626604	0.91	0.085307082	WP_050567087.1; non-ribosomal peptide synthetase [*Dickeya zeae*]
	JZL7_001422	1	0	0	0	WP_086642470.1; hypothetical protein [*Klebsiella pneumoniae*]
	JZL7_001423	1	0	0.621639344	0.621639344	WP_022633134.1; DUF736 domain-containing protein [*Dickeya solani*]
**Area J**	JZL7_004211	1	0.545217391	0	0	WP_038916971.1; hypothetical protein [*Dickeya zeae*]
	JZL7_004212	1	0.93	0.94	0	WP_012886556.1; conserved hypothetical protein [*Dickeya zeae*]
	JZL7_004213	1	0.86	0.55026087	0	WP_083068904.1; hypothetical protein [*Pantoea vagans*]
	JZL7_004214	1	0.224806202	0.312015504	0	WP_050088235.1; MULTISPECIES: hypothetical protein [*Yersinia* sp.]
	JZL7_004215	1	0.947641509	0.947641509	0	OOC13896.1; hypothetical protein BM451_09035, partial [*Dickeya dadantii*]
	JZL7_004216	1	0.99	0.98	0.259636364	WP_038926790.1; XRE family transcriptional regulator [*Dickeya zeae*]
	JZL7_004217	1	1	0.97	0.693294118	WP_027713493.1; MULTISPECIES: transcriptional regulator [*Dickeya*]
	JZL7_004218	1	0.962242991	0.962242991	0.298317757	WP_012886568.1; transcriptional regulator, XRE family [*Dickeya zeae* Ech586]
	JZL7_004219	1	0	0	0	WP_038921214.1; IS481 family transposase [*Dickeya dadantii*]
	JZL7_004221	1	0	0.85	0	WP_038916979.1; hypothetical protein [*Dickeya zeae*]
	JZL7_004221	1	0.54	0.81	0	WP_050568713.1; hypothetical protein [*Dickeya zeae*]
	JZL7_004222	1	0.6356676	0.859439776	0	WP_012886572.1; protein of unknown function DUF638 hemagglutinin/hemolysin putative [*Dickeya zeae* Ech586]
	JZL7_004223	1	0.432478632	0	0	WP_065824670.1; XRE family transcriptional regulator [*Photorhabdus asymbiotica*]
	JZL7_004224	1	0.416351351	0.551351351	0	WP_024106086.1; type I toxin-antitoxin system SymE family toxin [*Dickeya dianthicola*]
	JZL7_004225	1	0	0.81	0	WP_038902682.1; RNA 2'-phosphotransferase [*Dickeya dadantii*]
	JZL7_004226	1	0	0	0	WP_038901320.1; hypothetical protein [*Dickeya dadantii*]
	JZL7_004227	1	0.879495741	0.869199319	0	WP_038916980.1; hypothetical protein [*Dickeya zeae*]
	JZL7_004228	1	0.99	0.97	0	WP_038905980.1; RTX toxin-activating lysine-acyltransferase [*Dickeya zeae*]
	JZL7_004229	1	0.978185185	0.968203704	0.267	WP_038916981.1; ShlB/FhaC/HecB family hemolysin secretion/activation protein [*Dickeya zeae*]

Table S7 The prophages predicted in JZL7 genome

Phage ID	Length	Complete	Score	Start (nt)	End (nt)	GC (%)	Refence
1	34157	intact	150	1759168	1793324	53.37	PHAGE_Mannhe_vB_MhM_3927AP2_NC_028766(16),PHAGE_Haemop_SuMu_NC_019455(15),PHAGE_Pseudo_JBD25_NC_027992(9),PHAGE_Escher_D108_NC_013594(8),PHAGE_Entero_SfMu_NC_027382(8),PHAGE_Ralsto_RS138_NC_029107(7),PHAGE_Entero_Mu_NC_000929(5),PHAGE_Vibrio_12B12_NC_021070(5),PHAGE_Pseudo_D3112_NC_005178(5),PHAGE_Pseudo_JD024_NC_024330(4),PHAGE_Pseudo_JBD24_NC_020203(4),PHAGE_Pseudo_vB_PaeS_PAO1_Ab30_NC_026601(3),PHAGE_Pseudo_DMS3_NC_008717(3),PHAGE_Pseudo_JBD30_NC_020198(3),PHAGE_Pseudo_MP29_NC_011613(3),PHAGE_Pseudo_LPB1_NC_027298(3),PHAGE_Burkho_BcepMu_NC_005882(3),PHAGE_Pseudo_H70_NC_027384(2),PHAGE_Aeromo_vB_AsaM_56_NC_019527(2),PHAGE_Entero_HK106_NC_019768(2),PHAGE_Burkho_KS10_NC_011216(2),PHAGE_Pseudo_PA1phi_NC_023700(2),PHAGE_Pseudo_vB_PaeS_PM105_NC_028667(2),PHAGE_Pseudo_JBD5_NC_020202(2),PHAGE_Entero_phiP27_NC_003356(2),PHAGE_Salmon_118970_sal3_NC_031940(2),PHAGE_Entero_HK97_NC_002167(2),PHAGE_Pseudo_MP38_NC_011611(2),PHAGE_Pseudo_B3_NC_006548(2),PHAGE_Yersin_phiR1_37_NC_016163(2),PHAGE_Salmon_ST64B_NC_004313(1),PHAGE_Shigel_SfIV_NC_022749(1),PHAGE_Klebsi_phiKO2_NC_005857(1),PHAGE_Escher_TL_2011b_NC_019445(1),PHAGE_Entero_SfV_NC_003444(1),PHAGE_Stenot_vB_SmaS_DLP_2_NC_029019(1),PHAGE_Pseudo_MP42_NC_018274(1),PHAGE_Entero_HK225_NC_019717(1),PHAGE_Vibrio_1_NC_028799(1),PHAGE_Entero_N15_NC_001901(1),PHAGE_Entero_c_1_NC_019706(1),PHAGE_Pseudo_MP22_NC_009818(1),PHAGE_Vibri

							o_JA_1_NC_021540(1),PHAGE_Rhizob_vB_RleS_L338C_NC_023502(1),PHAGE_Pseudo_MP48_NC_024782(1),PHAGE_Escher_phiV10_NC_007804(1),PHAGE_Entero_SfI_NC_027339(1),PHAGE_Shigel_SfII_NC_021857(1),PHAGE_Burkho_phiE255_NC_009237(1),PHAGE_Cronob_ENT47670_NC_019927(1),PHAGE_Aeromo_PX29_NC_023688(1),PHAGE_Salmon_SPN1S_NC_016761(1),PHAGE_Salmon_epsilon15_NC_004775(1),PHAGE_Escher_pro483_NC_028943(1)
2	38275	incomplete	50	1817979	1856253	53.81	PHAGE_Mycoba_Xeno_NC_031243(2),PHAGE_Ralsto_RSY1_NC_025115(2),PHAGE_Vibrio_CTX_NC_015209(2),PHAGE_Paenib_Fern_NC_028851(1),PHAGE_Entero_P4_NC_001609(1),PHAGE_Escher_D108_NC_013594(1),PHAGE_Helico_1961P_NC_019512(1),PHAGE_Thermu_P2345_NC_009803(1),PHAGE_Entero_Mu_NC_000929(1),PHAGE_Yersin_L_413C_NC_004745(1),PHAGE_Mycoba_Redi_NC_023730(1),PHAGE_Thermu_P7426_NC_009804(1),PHAGE_Bacill_IEBH_NC_011167(1),PHAGE_Pseudo_phi297_NC_016762(1),PHAGE_Bacill_G_NC_023719(1),PHAGE_Ralsto_RS138_NC_029107(1),PHAGE_Helico_KHP40_NC_019931(1),PHAGE_Haemop_SuMu_NC_019455(1),PHAGE_Burkho_KL3_NC_015266(1),PHAGE_Mannhe_vB_MhM_587AP1_NC_028898(1),PHAGE_Cyanop_PSS2_NC_013021(1),PHAGE_Stenot_S1_NC_011589(1),PHAGE_Caulob_Cr30_NC_025422(1),PHAGE_Brevib_Jenst_NC_028805(1),PHAGE_Mannhe_phiMHaA1_NC_008201(1),PHAGE_Clostr_phiCT9441A_NC_029022(1),PHAGE_Bacill_250_NC_029024(1),PHAGE_Entero_phiFL4A_NC_013644(1),PHAGE_Helico_KHP30_NC_019928(1),PHAGE_Helico_phiHP33_NC_016568(1),PHAGE_Entero_SfMu_NC_027382(1),PHAGE_Clostr_PhiS63_NC_017978(1)

3	25134	intact	150	2690216	2715349	53.02	PHAGE_Escher_pro483_NC_028943(13),PHAGE_Entero_WPhi_NC_005056(10),PHAGE_Entero_PsP3_NC_005340(9),PHAGE_Entero_fiAA91_ss_NC_022750(9),PHAGE_Salmon_SP_004_NC_021774(8),PHAGE_Yersin_L_413C_NC_004745(7),PHAGE_Entero_186_NC_001317(7),PHAGE_Salmon_SEN1_NC_029003(6),PHAGE_Entero_P2_NC_001895(5),PHAGE_Escher_pro147_NC_028896(4),PHAGE_Erwini_ENT90_NC_019932(3),PHAGE_Salmon_Fels_2_NC_010463(3),PHAGE_Entero_HK97_NC_002167(2),PHAGE_Salmon_SEN5_NC_028701(2),PHAGE_Entero_HK106_NC_019768(2),PHAGE_Salmon_SEN4_NC_029015(2),PHAGE_Bacill_SP_15_NC_031245(1),PHAGE_Escher_D108_NC_013594(1),PHAGE_Entero_P1_NC_005856(1),PHAGE_Salmon_RE_2010_NC_019488(1),PHAGE_Entero_Mu_NC_000929(1),PHAGE_Entero_lambda_NC_001416(1),PHAGE_Pseudo_YuA_NC_010116(1),PHAGE_Entero_HK629_NC_019711(1),PHAGE_Pseudo_MP1412_NC_018282(1),PHAGE_Yersin_phiR1_37_NC_016163(1),PHAGE_Bacill_G_NC_023719(1),PHAGE_Entero_HK630_NC_019723(1)

4	59700	intact	150	3026641	3086340	50.93	PHAGE_Pectob_ZF40_NC_019522(44),PHAGE_Iodoba_phiPLPE_NC_011142(15),PHAGE_Edward_MSW_3_NC_020082(12),PHAGE_Edward_PEi21_NC_021342(12),PHAGE_Klebsi_JD001_NC_020204(11),PHAGE_Salmon_118970_sal3_NC_031940(9),PHAGE_Vibrio_vB_VchM_138_NC_019518(8),PHAGE_Salmon_118970_sal3_NC_031940(5),PHAGE_Escher_HK639_NC_016158(5),PHAGE_Vibrio_CP_T1_NC_019457(5),PHAGE_Entero_mEp390_NC_019721(5),PHAGE_Entero_phiP27_NC_003356(4),PHAGE_Entero_HK225_NC_019717(3),PHAGE_Escher_TL_2011b_NC_019445(2),PHAGE_Entero_mEpX1_NC_019709(2),PHAGE_Entero_WPhi_NC_005056(2),PHAGE_Salmon_SPN3UB_NC_019545(2),PHAGE_Salmon_SPN1S_NC_016761(2),PHAGE_Entero_ES18_NC_006949(2),PHAGE_Salmon_SP_004_NC_021774(2),PHAGE_Pseudo_PMG1_NC_016765(2),PHAGE_Entero_HK022_NC_002166(2),PHAGE_Entero_fiAA91_ss_NC_022750(2),PHAGE_Salmon_SEN34_NC_028699(2),PHAGE_Pseudo_F116_NC_006552(2),PHAGE_Escher_phiV10_NC_007804(2),PHAGE_Rhizob_RHEph06_NC_027296(2),PHAGE_Salmon_epsilon15_NC_004775(2),PHAGE_Mycoba_32HC_NC_023602(1),PHAGE_Entero_P22_NC_002371(1),PHAGE_Psychr_pOW20_A_NC_020841(1),PHAGE_Synech_S_CBS4_NC_016766(1),PHAGE_Burkho_Bcep22_NC_005262(1),PHAGE_Salmon_ST64B_NC_004313(1),PHAGE_Salmon_SP_076_NC_021782(1),PHAGE_Burkho_BcepC6B_NC_005887(1),PHAGE_Escher_bV_EcoS_AHS24_NC_024784(1),PHAGE_Haemop_Aaphi23_NC_004827(1),PHAGE_Pectob_phiTE_NC_020201(1),PHAGE_Yersin_L_413C_NC_004745(1),PHAGE_Entero_phi80_NC_021190(1),PHAGE_Burkho_BcepIL02_NC_012743(1),PHAGE_Entero_HK629_NC_019711(1),PHAGE_Cronob_CR8_NC_024354(1),PHAGE_Phage_Gifsy_1_NC_010392(1),PHAGE_Brevib_Sundance_NC_028749(1),PHAGE_Cronob_phiES15_NC_018454(1),PHAGE_Bacill_PM1_NC_020883(1),PHAGE_Entero_PsP3_NC_005340(1),PHAGE_Stap

							hy_37_NC_007055(1),PHAGE_Shigel_POCJ13_NC_025434(1),PHAGE_Acidia_virus_NC_029316(1),PHAGE_Phage_Gifsy_2_NC_010393(1),PHAGE_Erwini_PEp14_NC_016767(1),PHAGE_Staphy_EW_NC_007056(1),PHAGE_Burkho_phi6442_NC_009235(1),PHAGE_Sulfol_SMV2_NC_029020(1),PHAGE_Stenot_S1_NC_011589(1),PHAGE_Stx2_II_NC_004914(1),PHAGE_Escher_Pollock_NC_027381(1),PHAGE_Entero_186_NC_001317(1),PHAGE_Lister_2389_NC_003291(1),PHAGE_Cronob_ENT39118_NC_019934(1),PHAGE_Burkho_DC1_NC_018452(1),PHAGE_Shigel_SfII_NC_021857(1),PHAGE_Cronob_ENT47670_NC_019927(1),PHAGE_Burkho_BcepMigl_NC_019917(1),PHAGE_Burkho_phiE125_NC_003309(1),PHAGE_Entero_VT2_Sakai_NC_000902(1),PHAGE_Vibrio_X29_NC_024369(1),PHAGE_Escher_phage_NC_004913(1),PHAGE_Escher_pro483_NC_028943(1),PHAGE_Mycoba_Che9c_NC_004683(1),PHAGE_Erwini_phiEt88_NC_015295(1),PHAGE_Shigel_SfIV_NC_022749(1),PHAGE_Azospi_Cd_NC_010355(1),PHAGE_Shewan_Spp001_NC_023594(1),PHAGE_Shigel_Ss_VASD_NC_028685(1),PHAGE_Pseudo_KPP12_NC_019935(1),PHAGE_Vibrio_VBM1_NC_020850(1),PHAGE_Vibrio_VvAW1_NC_020488(1),PHAGE_Salmon_vB_SosS_Oslo_NC_018279(1),PHAGE_Salmon_SP_058_NC_021772(1),PHAGE_Bacill_G_NC_023719(1),PHAGE_Stx2_c_1717_NC_011357(1),PHAGE_Pseudo_phiCTX_NC_003278(1),PHAGE_Xantho_vB_XveM_DIBBI_NC_017981(1),PHAGE_Bacill_vB_BanS_Tsamsa_NC_023007(1),PHAGE_Salmon_SEN1_NC_029003(1),PHAGE_Rhizob_RR1_A_NC_021560(1),PHAGE_Salmon_RE_2010_NC_019488(1),PHAGE_Entero_SfI_NC_027339(1),PHAGE_Edward_GF_2_NC_026611(1),PHAGE_Cronob_02_NC_028672(1),PHAGE_Lister_LP_101_NC_024387(1),PHAGE_Salmon_SEN22_NC_028696(1),PHAGE_Salmon_Fels_2_NC_010463(1),PHAGE_Entero_P2_NC_001895(1),PHAGE_Cronob_CR3_NC_017974(1),PHAGE_Shigel_Stx_NC_029120(1)

5	26427	incomplete	20	3081974	3108400	48.21	PHAGE_Klebsi_vB_KpnM_KpV477_NC_031087(2),PHAGE_Ralsto_RSY1_NC_025115(2),PHAGE_Entero_P4_NC_001609(1),PHAGE_Paenib_Fern_NC_028851(1),PHAGE_Salmon_SEN1_NC_029003(1),PHAGE_Erwini_ENT90_NC_019932(1),PHAGE_Escher_D108_NC_013594(1),PHAGE_Mannhe_vB_MhM_587AP1_NC_028898(1),PHAGE_Brevib_Jimmer1_NC_029104(1),PHAGE_Entero_Mu_NC_000929(1),PHAGE_Stenot_S1_NC_011589(1),PHAGE_Bacill_BM5_NC_029069(1),PHAGE_Strept_VWB_NC_005345(1),PHAGE_Mannhe_phiMHaA1_NC_008201(1),PHAGE_Brevib_Davies_NC_022980(1),PHAGE_Brevib_Osiris_NC_028969(1),PHAGE_Campyl_NCTC12673_NC_015464(1),PHAGE_Pseudo_phi297_NC_016762(1),PHAGE_Entero_phiFL4A_NC_013644(1),PHAGE_Bacill_G_NC_023719(1),PHAGE_Entero_PsP3_NC_005340(1),PHAGE_Ralsto_RS138_NC_029107(1),PHAGE_Klebsi_JD18_NC_028686(1),PHAGE_Campyl_CP30A_NC_018861(1),PHAGE_Campyl_CPX_NC_016562(1),PHAGE_Klebsi_PKO111_NC_031095(1),PHAGE_Haemop_SuMu_NC_019455(1),PHAGE_Entero_SfMu_NC_027382(1),PHAGE_Clostr_phiCD211_NC_029048(1),PHAGE_Burkho_KL3_NC_015266(1),PHAGE_Clostr_PhiS63_NC_017978(1)
6	6493	questionable	70	3347529	3354021	47.65	PHAGE_Shigel_SfII_NC_021857(3),PHAGE_Shigel_Sf6_NC_005344(2),PHAGE_Shigel_SfIV_NC_022749(2),PHAGE_Entero_SfV_NC_003444(2),PHAGE_Entero_SfI_NC_027339(2),PHAGE_Salmon_118970_sal3_NC_031940(1),PHAGE_Salmon_g341c_NC_013059(1),PHAGE_Salmon_vB_SemP_Emek_NC_018275(1),PHAGE_Erwini_phiEt88_NC_015295(1),PHAGE_Salmon_118970_sal3_NC_031940(1),PHAGE_Entero_VT2phi_272_NC_028656(1)

Table S8 Gene families present in genomes of EC1 and MS2 but absent in the JZL7 genome

ID	Gene number in every sample			Gene list in every sample			Gene name	Best hit in NR database	
	MS2	EC1	JZL7	MS2	EC1	JZL7		Protein id	Description
1	1	1	0	C1O30_RS00140	W909_00075	/		WP_050568641.1	4Fe-4S cluster-binding domain-containing protein
2	1	1	0	C1O30_RS00145	W909_00080	/		WP_102800761.1	ATP-grasp domain-containing protein
3	1	1	0	C1O30_RS00150	W909_00085	/		WP_102800762.1	2OG-Fe dioxygenase family protein
4	1	1	0	C1O30_RS00155	W909_00090	/		WP_038915280.1	MFS transporter
5	11	6	0	C1O30_RS01290 C1O30_RS01670 C1O30_RS02885 C1O30_RS05375 C1O30_RS09640 C1O30_RS10910 C1O30_RS10965 C1O30_RS14335 C1O30_RS15570 C1O30_RS18205 C1O30_RS18320	W909_00510 W909_01215 W909_02895 W909_03125 W909_08545 W909_11730	/		WP_102800868.1	IS4 family transposase
6	1	1	0	C1O30_RS01370	W909_01295	/	*dmsC*	WP_102800873.1	dimethyl sulfoxide reductase anchor subunit
7	1	1	0	C1O30_RS01375	W909_01300	/	*dmsB*	WP_016943134.1	4Fe-4S dicluster domain-containing protein
8	1	1	0	C1O30_RS01380	W909_01305	/	*dmsA*	WP_038915404.1	molybdopterin-dependent oxidoreductase
9	1	1	0	C1O30_RS01385	W909_01310	/	*dmsD*	WP_019843541.1	hypothetical protein
10	1	1	0	C1O30_RS01390	W909_01315	/		WP_038912905.1	SDR family oxidoreductase
11	1	1	0	C1O30_RS01505	W909_01430	/	*aaeB*	WP_102800883.1	p-hydroxybenzoic acid efflux pump subunit AaeB
12	1	1	0	C1O30_RS02130	W909_02175	/		WP_102800931.1	hypothetical protein
13	1	1	0	C1O30_RS02370	W909_02455	/		WP_102800953.1	hypothetical protein
14	1	1	0	C1O30_RS02465	W909_02565	/		WP_102800962.1	hypothetical protein
15	1	1	0	C1O30_RS02510	W909_02610	/		WP_102800967.1	GNAT family N-acetyltransferase
16	1	1	0	C1O30_RS02660	W909_02700	/	*ccdB*	WP_102800987.1	type II toxin-antitoxin system toxin CcdB
17	1	1	0	C1O30_RS02665	W909_02705	/	*ccdA*	WP_102800988.1	type II toxin-antitoxin system antitoxin CcdA
18	1	1	0	C1O30_RS02835	W909_02890	/	*pnl*	WP_038915541.1	pectate lyase
19	1	1	0	C1O30_RS03460	W909_03680	/		WP_038907102.1	LysE family transporter
20	1	1	0	C1O30_RS03475	W909_03695	/		WP_080638945.1	GNAT family N-acetyltransferase
21	1	1	0	C1O30_RS04230	W909_03955	/		WP_019844445.1	PAS domain-containing protein
22	1	1	0	C1O30_RS04475	W909_04165	/		WP_023639033.1	SDR family oxidoreductase

23	1	1	0	C1O30_RS05105	W909_15495	/		WP_102801219.1	HAMP domain-containing protein
24	1	1	0	C1O30_RS06290	W909_05970	/		WP_102801310.1	DUF1722 domain-containing protein
25	1	1	0	C1O30_RS06880	W909_06560	/		WP_102801362.1	hypothetical protein
26	1	1	0	C1O30_RS07140	W909_06860	/		WP_102801396.1	hypothetical protein
27	1	1	0	C1O30_RS07335	W909_06975	/	*lysR*	WP_026357913.1	LysR family transcriptional regulator
28	1	1	0	C1O30_RS07340	W909_06980	/		WP_102802686.1	NADP-dependent oxidoreductase
29	1	1	0	C1O30_RS07345	W909_06985	/		WP_019844756.1	antibiotic biosynthesis monooxygenase
30	1	1	0	C1O30_RS07575	W909_12980	/		WP_057085392.1	hypothetical protein
31	1	1	0	C1O30_RS07580	W909_12975	/		WP_102801447.1	AAA family ATPase
32	1	1	0	C1O30_RS07840	W909_07260	/		WP_102801472.1	FMN-binding negative transcriptional regulator
33	1	1	0	C1O30_RS08725	W909_07805	/		WP_102801592.1	glycosyltransferase
34	1	1	0	C1O30_RS09650	W909_08555	/		WP_102801673.1	glutathione S-transferase
35	1	1	0	C1O30_RS11095	W909_10035	/	*dspF*	WP_102801795.1	DspFAvrF family protein
36	1	1	0	C1O30_RS11100	W909_10040	/	*dspE*	WP_102801796.1	AvrE-family type 3 secretion system effector
37	1	1	0	C1O30_RS11105	W909_10045	/	*hrpZ*	WP_102801797.1	type III effector protein
38	1	1	0	C1O30_RS11115	W909_10050	/	*hrpW*	WP_102801799.1	DNA-binding protein
39	1	2	0	C1O30_RS11550	W909_RS10365 W909_RS20605	/		WP_102801827.1	filamentous hemagglutinin N-terminal domain-containing protein
40	1	1	0	C1O30_RS11560	W909_10690	/	*hrpN*	WP_023639571.1	Harpin hrpN
41	1	1	0	C1O30_RS11570	W909_10700	/	*hrpT*	WP_102801829.1	type III secretion protein HrpT
42	1	1	0	C1O30_RS11575	W909_10705	/	*hrcC*	WP_102801830.1	EscC/YscC/HrcC family type III secretion system outer membrane ring protein
43	1	1	0	C1O30_RS11580	W909_10710	/	*hrpG*	WP_102801831.1	serine kinase
44	1	1	0	C1O30_RS11585	W909_10715	/	*hrpF*	WP_033568581.1	serine kinase
45	1	1	0	C1O30_RS11620	W909_10730	/	*hrpD*	WP_050567521.1	hypothetical protein
46	1	1	0	C1O30_RS11625	W909_10735	/	*hrcJ*	WP_102801837.1	EscJ/YscJ/HrcJ family type III secretion inner membrane ring protein
47	1	1	0	C1O30_RS11630	W909_10740	/	*hrpB*	WP_102801838.1	serine kinase
48	1	1	0	C1O30_RS11635	W909_10745	/	*hrpA*	WP_023639560.1	hypothetical protein
49	1	1	0	C1O30_RS11640	W909_10750	/	*hrpS*	WP_102801839.1	sigma-54-dependent Fis family transcriptional regulator
50	1	1	0	C1O30_RS11645	W909_10755	/	*hrpY*	WP_012884573.1	response regulator transcription factor
51	1	1	0	C1O30_RS11650	W909_10760	/	*hrpX*	WP_102801840.1	PAS domain S-box protein
52	1	1	0	C1O30_RS11655	W909_10765	/	*hrpL*	WP_023639557.1	RNA polymerase sigma factor

53	1	1	0	C1O30_RS11660	W909_10770	/	*hrpJ*	WP_102801841.1	YopN family type III secretion system gatekeeper subunit
54	1	1	0	C1O30_RS11665	W909_10775	/	*hrcV*	WP_102801842.1	EscV/YscV/HrcV family type III secretion system export apparatus protein
55	1	1	0	C1O30_RS11670	W909_10780	/	*hrpQ*	WP_102801843.1	EscD/YscD/HrpQ family type III secretion system inner membrane ring protein
56	1	1	0	C1O30_RS11675	W909_10785	/	*hrcN*	WP_102801844.1	EscN/YscN/HrcN family type III secretion system ATPase
57	1	1	0	C1O30_RS11680	W909_10790	/	*hrpO*	WP_102801845.1	type III secretion protein
58	1	1	0	C1O30_RS11685	W909_10795	/	*hrpP*	WP_102801846.1	type III secretion protein HrpP
59	1	1	0	C1O30_RS11690	W909_10800	/	*hrcQ*	WP_102801847.1	YscQ/HrcQ family type III secretion apparatus protein
60	1	1	0	C1O30_RS11695	W909_10805	/	*hrcR*	WP_102801848.1	EscR/YscR/HrcR family type III secretion system export apparatus protein
61	1	1	0	C1O30_RS11700	W909_10810	/	*hrcS*	WP_102801849.1	EscS/YscS/HrcS family type III secretion system export apparatus protein
62	1	1	0	C1O30_RS11705	W909_10815	/	*hrcT*	WP_102801850.1	EscT/YscT/HrcT family type III secretion system export apparatus protein
63	1	1	0	C1O30_RS11710	W909_10820	/	*hrcU*	WP_102801851.1	EscU/YscU/HrcU family type III secretion system export apparatus switch protein
64	1	1	0	C1O30_RS12525	W909_11520	/		WP_019845792.1	DUF2335 domain-containing protein
65	1	1	0	C1O30_RS13675	W909_13155	/		WP_102802003.1	LD-carboxypeptidase
66	1	1	0	C1O30_RS14540	W909_RS13560	/		WP_102802079.1	diguanylate cyclase
67	1	1	0	C1O30_RS14575	W909_14085	/		WP_102802732.1	hypothetical protein
68	1	1	0	C1O30_RS16170	W909_02100	/		WP_102802207.1	DUF4942 domain-containing protein
69	1	1	0	C1O30_RS16175	W909_02095	/		WP_102802208.1	toxin
70	1	1	0	C1O30_RS16185	W909_02090	/		WP_012768688.1	DNA repair protein RadC
71	1	1	0	C1O30_RS16190	W909_02085	/		WP_102802210.1	DUF945 domain-containing protein
72	1	1	0	C1O30_RS16195	W909_02080	/		WP_102802211.1	GTPase family protein
73	1	2	0	C1O30_RS16250	W909_RS20490 W909_RS20640	/		WP_102802220.1	IS5 family transposase
74	1	1	0	C1O30_RS18380	W909_17580	/		WP_102802407.1	hypothetical protein
75	1	1	0	C1O30_RS18965	W909_18250	/		WP_019845195.1	GntR family transcriptional regulator
76	1	1	0	C1O30_RS18970	W909_18255	/		WP_102802443.1	ABC transporter substrate-binding protein
77	1	1	0	C1O30_RS18975	W909_18260	/		WP_019845193.1	amino acid ABC transporter permease
78	1	1	0	C1O30_RS18980	W909_18265	/		WP_102802444.1	amino acid ABC transporter ATP-binding protein

79	1	1	0	C1O30_RS18985	W909_18270	/		WP_102802445.1	gamma-glutamyltransferase family protein
80	1	1	0	C1O30_RS20195	W909_19330	/		WP_102802551.1	NUDIX domain-containing protein
81	1	1	0	C1O30_RS20370	W909_19490	/		WP_102802573.1	methyl-accepting chemotaxis protein
82	1	1	0	C1O30_RS20725	W909_19815	/		WP_102802606.1	methyl-accepting chemotaxis protein
